# CellProfiler: image analysis software for identifying and quantifying cell phenotypes

**DOI:** 10.1186/gb-2006-7-10-r100

**Published:** 2006-10-31

**Authors:** Anne E Carpenter, Thouis R Jones, Michael R Lamprecht, Colin Clarke, In Han Kang, Ola Friman, David A Guertin, Joo Han Chang, Robert A Lindquist, Jason Moffat, Polina Golland, David M Sabatini

**Affiliations:** 1 Whitehead Institute for Biomedical Research, Cambridge, MA 02142, USA; 2 Computer Sciences and Artificial Intelligence Laboratory, Massachusetts Institute of Technology, Cambridge, MA 02142, USA; 3 Department of Radiology, Brigham and Women's Hospital, Boston, MA 02115, USA; 4 Department of Biology, Massachusetts Institute of Technology, Cambridge, MA 02142, USA

## Abstract

Biologists can now prepare and image thousands of samples per day using automation, enabling chemical screens and functional genomics (for example, using RNA interference). Here we describe the first free, open-source system designed for flexible, high-throughput cell image analysis, CellProfiler. CellProfiler can address a variety of biological questions quantitatively, including standard assays (for example, cell count, size, per-cell protein levels) and complex morphological assays (for example, cell/organelle shape or subcellular patterns of DNA or protein staining).

## Abstract 2

CellProfiler, the first free, open-source system for flexible and high-throughput cell image analysis is described.

## Rationale

Examining cells by microscopy has long been a primary method for studying cellular function. When cells are stained appropriately, visual analysis can reveal biological mechanisms. Advanced microscopes can now, in a single day, easily collect thousands of high resolution images of cells from time-lapse experiments and from large-scale screens using chemical compounds, RNA interference (RNAi) reagents, or expression plasmids [1-5]. However, a bottleneck exists at the image analysis stage. Several pioneering large screens have been scored through visual inspection by expert biologists [6,7], whose interpretive ability will not soon be replicated by a computer. Still, for most applications, image cytometry (automated cell image analysis) is strongly preferable to analysis by eye. In fact, in some cases image cytometry is absolutely required to extract the full spectrum of information present in biological images, for reasons we discuss here.

First, while human observers typically score one or at most a few cellular features, image cytometry simultaneously yields many informative measures of cells, including the intensity and localization of each fluorescently labeled cellular component (for example, DNA or protein) within each subcellular compartment, as well as the number, size, and shape of those subcellular compartments. Image-based analysis is thus versatile, inherently multiplexed, and high in information content. Like flow cytometry, image cytometry measures the per-cell amount of protein and DNA, but can more conveniently handle hundreds of thousands of distinct samples and is also compatible with adherent cell types, time-lapse samples, and intact tissues. In addition, image cytometry can accurately measure protein texture and localization as well as cell shape and size.

Second, human-scored image analysis is qualitative, usually categorizing samples as 'hits' (where normal physiology is grossly disturbed) or 'non-hits'. By contrast, automated analysis rapidly produces consistent, quantitative measures for every image. In addition to uncovering subtle samples of interest that would otherwise be missed, systems-level conclusions can be drawn directly from the quantitative measures for every image. Measuring a large number of features, even features undetectable by eye, has proven useful for screening as well as cytological/cytometric profiling, which can group similar genes or reveal a drug's mechanism of action [3,8-14].

Third, image cytometry individually measures each cell rather than producing a score for the entire image. Because individual cells' responses are inhomogeneous [15], multiparametric single cell data from several types of instruments have proven much more powerful than whole-population data (for example, western blots or mRNA expression chips) for clustering genes, deriving causal networks, classifying protein localization, and diagnosing disease [10,16-18]. In addition, individual cell measurements can reveal samples that differ in only a subpopulation of cells, which would otherwise be masked in whole-population measures.

Fourth, quantitative image analysis is able to detect some features that are not readily detectable by a human observer. For example, the two-fold difference in DNA staining intensity that reveals whether a cell is in G1 or G2 phase of the cell cycle are measurable by computer but are difficult for the human eye to observe in cell images. Furthermore, small but biologically significant differences, for example, a 10% increase in nucleus size, are not noticeable by eye. Other features, for example, the texture (smoothness) of protein or DNA staining, are observable but not quantifiable by eye. Pathologists have known for years that changes in DNA or protein texture can correlate to profound and otherwise undetectable changes in cell physiology, a fact used in diagnosis of disease [17,19]. Even changes not visible to the human eye can reveal disease state [20].

Fifth, image cytometry is much less labor-intensive and higher-throughput. Appropriate software produces reliable results from a large-scale experiment in hours, versus months of tedious visual inspection. This improvement is more than an incremental technical advance, because it relieves the one remaining bottleneck to routinely conducting such experiments.

Prior to the work presented here, the only flexible, open-source biological image analysis package was ImageJ/NIH Image [21]. This package has been successfully used by many laboratories. Its design, however, is geared more towards the analysis of individual images (comparable to Adobe Photoshop) rather than flexible, high-throughput work. Macros can be written in ImageJ for high-throughput work but adapting macros to new projects requires that biologists learn a programming language.

While not creating a general, flexible software tool, many groups have benefited from automated cell image analysis by developing their own scripts, macros, and plug-ins to accomplish specific image analysis tasks. Custom programs written in commercial software (for example, MetaMorph, ImagePro Plus, MATLAB) or Java have been used to identify, measure, and track cells in images and time lapse movies [10,22,23]. Such studies clearly show the power of automated image analysis for biological discovery. However, most of these custom programs are not modular, so combining several steps and changing settings requires interacting directly with the code and is simply not practical for routinely processing hundreds of thousands of images or sending jobs to a cluster. The effort expended by laboratories in creating an analysis solution with a particular software package is often lost after the initial experiment is completed; other laboratories rarely use the methods because they are customized for a particular cell type, assay or even image set. Furthermore, although developing a routine for a new cell type or assay usually requires testing multiple algorithms, it is impractical to implement and test several published methods for a particular project.

Commercial software has also been developed, mainly for the pharmaceutical screening market, by companies including Cellomics, TTP LabTech, Evotec, Molecular Devices, and GE Healthcare [24]. Development of these packages has been guided mainly by mammalian cell types and cellular features of pharmaceutical interest, including protein translocation, micronucleus formation, neurite outgrowth, and cell count [25]. The high cost and the bundling of commercial software with hardware makes it impractical to test several programs for a new project. The proprietary nature of the code prevents researchers from knowing the strategy of a given algorithm and it cannot be modified if desired. As is the case with many laboratories, we have found commercial packages useful for some screens in mammalian cells, but in other cases limiting [1,5,26,27].

Furthermore, key challenges remain in image analysis algorithm development itself [28]. Cell image analysis has been described as one of the greatest remaining challenges in screening [5,29], and as a field is "very much in its infancy" [30] and "lag [s] behind the adoption of high-throughput imaging technologies" [10]. Accurate cell identification is required to extract meaningful measures from images, but even for mammalian cell types, existing software often fails on crowded cell samples, which has severely limited screens thus far. Screens in most non-mammalian organisms have been limited to visual inspection.

In summary, while existing software enables particular assays for particular cell types, high throughput image analysis has, to this point, been impractical unless an image analysis expert develops a customized solution, or unless commercial packages are used with their built-in algorithms for a limited set of cellular features and for a limited set of cell types. There exists a clear need for a powerful, flexible, open-source platform for high-throughput cell image analysis.

Here we describe the open-source CellProfiler project, our effort to develop such a software system for the scientific community. CellProfiler simultaneously measures the size, shape, intensity and texture of a variety of cell types in a high throughput manner. Note that we focus in this paper not on the technical details of the software (which are described in the manual), nor computational validation of the mostly published algorithms, nor on a mechanistic study of any particular biological finding. Rather, we describe the system, validate the software for a variety of real-world biological problems, demonstrate the breadth of its utility (including on various cell types and assays), and hope to stimulate ideas within the biological community for future applications of the software.

## Overview of the software system

The following can be freely downloaded from the CellProfiler website [31]: CellProfiler for Windows, Mac, and Unix (compiled, not requiring MATLAB); CellProfiler's MATLAB source code; a full technical description of CellProfiler's algorithms and measurements in an extensive PDF formatted manual (Additional data file 1), identical to the information found in help buttons within CellProfiler; and pipelines to identify the various cell types in this paper (see Additional data file 2 for a list of the modules in each pipeline).

CellProfiler is freely available modular image analysis software that is capable of handling hundreds of thousands of images. The software contains already-developed methods for many cell types and assays and is also an open-source, flexible platform for the sharing, testing, and development of new methods by image analysis experts. CellProfiler meets the needs discussed in the introduction, in that it contains: advanced algorithms for image analysis that are able to accurately identify crowded cells and non-mammalian cell types; a modular, flexible design allowing analysis of new assays and phenotypes; open-source code so the underlying methodology is known and can be modified or improved by others; a user-friendly interface; the capability to make use of clusters of computers when available; and a design that eliminates the tedium of the many steps typically involved in image analysis, many of which are not easily transferable from one project to another (for example, image formatting, combining several image analysis steps, or repeating the analysis with slightly different parameters). CellProfiler was designed and optimized for the most common high-content screening image format, that is, two-dimensional images. It has very limited support for time-lapse and three-dimensional image stack analysis, although researchers interested in these areas could build compatible modules.

**Figure 1:**
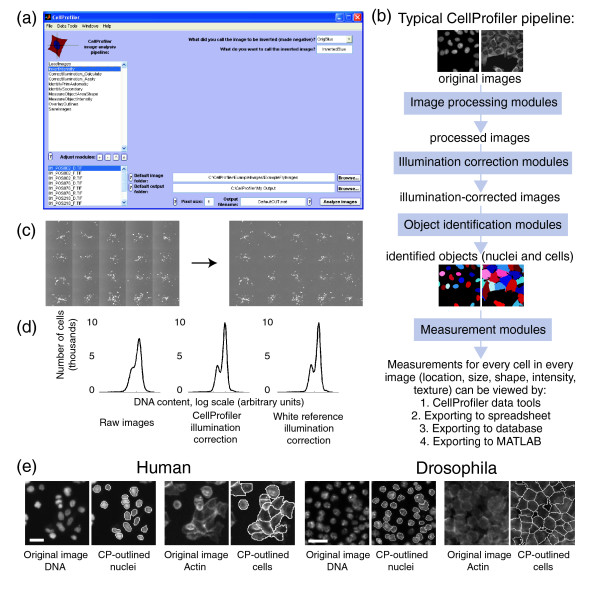
CellProfiler overview and features. **(a) **Main CellProfiler interface, with an analysis pipeline displayed. **(b) **Schematic of a typical CellProfiler pipeline. **(c) **Image processing example: uneven illumination from the left to the right within each field of view is noticeable in this three row by five column tiled image (left). CellProfiler's illumination correction modules correct these anomalies (right). Images were contrast-enhanced to display this effect. **(d) **These corrections reduce noise in quantitative measurements, demonstrated here in DNA content measures (middle) from images of *Drosophila *Kc167 cells that are improved over the raw images (left). The results are comparable to those produced by white referenced images (right), but they do not require the error prone and often omitted step of collecting a white reference image immediately before image acquisition. **(e) **Outlines show the identification of nuclei and identification of cell edges made by CellProfiler in human HT29 (left) and *Drosophila *Kc167 (right) cells. Cells touching the border are intentionally excluded from analysis and images were contrast stretched for display. Scale bars = 15 μm.

Most image analysis projects, even for new cell types or assays, can be accomplished simply by pointing and clicking using CellProfiler's graphical user interface (Figure 1a). The software uses the concept of a 'pipeline' of individual modules (Figure 1b; Additional data file 2). Each module processes the images in some manner, and the modules are placed in sequential order to create a pipeline: usually image processing, then object identification, then measurement. Over 50 CellProfiler modules are currently available (Additional data file 3). Most modules are automatic, but the software also allows interactive modules (for example, the user clicks to outline a region of interest in each image). Modules are mixed and matched for a specific project and each module's settings are adjusted appropriately. Upon starting the analysis, each image (or group of images if multiple wavelengths are available) travels through the pipeline and is processed by each module in order.

The pipeline's modules and their settings are saved and can be used to reproduce the analysis or share with colleagues. Many example pipelines are provided at the CellProfiler website [31] to provide a starting point for new analyses. To explain some features of CellProfiler, we describe in the subsequent sections the general steps in a typical pipeline.

## Image processing, including illumination correction

One of the most critical steps in image analysis is illumination correction. Illumination often varies more than 1.5-fold across the field of view, even when using fiber optic light sources, and occasionally even when images are thought to be already illumination-corrected by commercial image analysis software packages (TRJ, AEC, DMS, and PG, unpublished data). This adds an unacceptable level of noise, obscures real quantitative differences, and prevents many types of biological experiments that rely on accurate fluorescence intensity measurements (for example, DNA content of a nucleus, which only varies by two-fold during the cell cycle). CellProfiler contains standard methods plus our new methods [32] to address illumination variation, allowing various methods to be compared side by side and, ultimately, providing less noisy quantitative measures (Figure 1c,d). We use these illumination correction methods for every high-throughput image set we process, because using raw images degrades intensity measurements and, less obviously, can preclude accurate cell identification. This adversely affects all types of measurements, from intensity based measures (for example, DNA content histograms [1]) to area and shape measurements (TRJ, AEC, DMS, and PG, unpublished data). CellProfiler's other image processing modules perform other needed adjustments prior to identifying cells in images, for example, aligning or cropping (Additional data file 3).

## Cell identification

Object identification (also called segmentation) is the most challenging step in image analysis and its accuracy determines the accuracy of the resulting cell measurements. CellProfiler's object identification modules contain a variety of published and tested algorithms for identifying cells based on fluorescence, including work from our own group and others (Figure 1e). In most biological images, cells touch each other, causing the simple, fast algorithms used in some commercial software packages to fail. The first objects identified in an image (called primary objects) are often nuclei identified from DNA-stained images, although primary objects can also be whole cells, beads, speckles, tumors, and so on. Several simple algorithms are built into CellProfiler for cases where primary objects are well-dispersed, non-confluent, and bright relative to the background. More importantly, to effectively identify clumped objects, CellProfiler contains a modular three-step strategy based on previously published algorithms [33-37]. First, clumped objects are recognized and separated; second, the dividing lines between objects are found; and third, some of the resulting objects are either removed or merged together based on their measurements, for example, their size or shape.

After primary objects (often nuclei) are identified, the edges of secondary objects that surround each primary object (often cell edges) can be found more easily. Measuring cell size in *Drosophila *was not previously feasible because the commonly used watershed method [37] often fails to find the borders between clumped cells. We have, therefore, implemented in CellProfiler an improved Propagate algorithm [38], in addition to several standard methods of secondary object identification. Other subcellular compartments can also be identified, including the cytoplasm (the part of each cell excluding the nucleus) and the cell or nuclear membrane (the edge of the cell or nucleus).

The technical description of these algorithms is omitted here but is available in the online help and manual, in addition to previously published references cited therein (Additional data file 1). The identification modules include a 'test mode' for comparing several algorithms side by side in order to choose the best approach. We have found that these cell identification methods are flexible to various cell morphologies. This flexibility is convenient but, more importantly, often allows accurate identification of cells with unusual morphologies within a population of normal cells.

## Measurements and data analysis

CellProfiler measures a large number of features for each identified cell or subcellular compartment, including area, shape, intensity, and texture (each feature is described in Additional data file 4). This includes many standard features [39,40], but also complex measurements like Zernike shape features [41], and Haralick and Gabor texture features [42-44], which are described in detail in the online help and manual. There are also modules to measure various features (for example, intensity, texture, saturation, blur, area occupied by a stain) of an image in its entirety. A severe limitation of most commercial software is the inability to adapt to new biological questions by calculating new features from identified cells [5]. By contrast, CellProfiler's modular design and open-source code allows quickly measuring new cellular phenotypes as needed.

Measurements are accessible in several ways: using CellProfiler's built-in viewing and plotting data tools (Additional data file 5); exporting in a tab-delimited spreadsheet format that can be opened in programs like Microsoft Excel or OpenOffice Calc; exporting in a format that can be imported into a database like Oracle or MySQL; or directly in MATLAB.

## Usability

Like most new software in the laboratory, the process of setting up a CellProfiler analysis may take several days if the user is learning the software for the first time. Several resources help at this stage: the built-in help, the manual (Additional data file 1), the online discussion forum [31], the 'test mode' for the Identify modules that show results from various options side by side, and built-in image and data tools to interact with processed images and cell measurements (Additional data file 5). The flexible, modular design and point-and-click interface make setting up an analysis feasible for non-programmers. Over time, experienced users typically require less than a day to set up an entirely new experiment (for example, a new cell type or unusual measurement scheme). When performing the same analysis on different image sets where sample preparation is the only variable, we test the analysis on a few sample images and sometimes change one or two settings in the Identify modules. This takes less than an hour and is essentially a quality control step. Once a pipeline is satisfactory, analysis can be performed on the local computer or automatically divided into smaller batches to be sent to a cluster of computers, described in more detail in later sections.

CellProfiler's code is open-source under the GNU public license. Its image handling is flexible: there is no requirement for images to have a certain naming structure and many standard image formats plus some movie formats are supported. Its modular structure allows experts to expand the software to new file formats or add new algorithms. The source code was written in MATLAB because it is a powerful, easy to learn language, commonly used for scientific applications, including prototyping image analysis routines. Because the source code is well-documented, it is understandable even by non-programmers. Computationally intensive tasks use either MATLAB's native compiled functions or our own compiled C++ implementations to improve the speed. Analysis times vary widely depending on the image size, the number of objects found per image, and the number of features measured, but typical pipelines require from 20 seconds to five minutes per image on standard desktop computers.

## Validation of CellProfiler for many phenotypes

**Figure 2:**
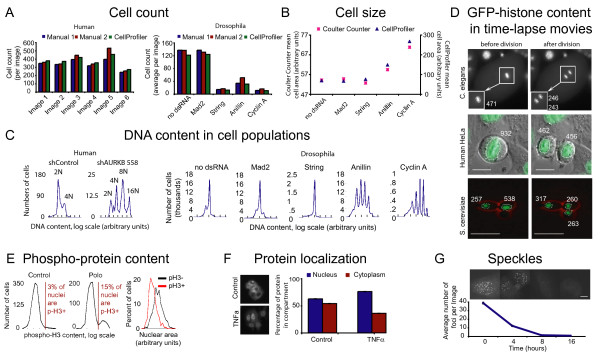
Validation of CellProfiler for many cellular phenotypes. **(a) **Cell count: for a set of 6 images of wild-type human HT29 cells (left), two researchers' counts varied by 11%, and CellProfiler's counts were within 6% of their average. For images of *Drosophila *Kc167 cells with various genes knocked down by RNAi (right), the two researchers' counts varied by 16% and CellProfiler's counts were within 17% of their average. Example images and CellProfiler outlines for these cell types are shown in Figure 1e. **(b) **Cell size: CellProfiler's cell area measurements are comparable to those of a Coulter particle counter for *Drosophila *Kc167 cells, for wild-type (no dsRNA) and RNA-interference induced samples. The SEM is too small to show error bars. **(c) **DNA content in cell populations: measurements are shown for human HT29 cell populations (1 image for each RNAi condition, left) and for *Drosophila *Kc167 cell populations (1,750 images for each RNAi condition were combined, right). The cell cycle distributions are as expected, with the 2N peak being predominant in the wild-type human sample, whereas most wild-type *Drosophila *nuclei are known to have 4N DNA content [62]. RNAi-targeted samples were also as expected for Aurora kinase B (polyploid), Mad2 (fairly normal cell cycle distribution), String (4N-enriched), and Anillin and Cyclin A (both polyploid). **(d) **Chromatin content in time lapse movies: GFP-histone H4 (*S. cerevisiae*) or GFP-histone H2B (HeLa and *C. elegans*) content is shown near each nucleus in arbitrary intensity units. The histone content is decreased by roughly half in each daughter nucleus after division. For *C. elegans*, only the boxed region of interest was analyzed. Scale bars: *C. elegans*, unknown; human HeLa = 20 μm; *S. cerevisiae *= 10 μm. **(e) **Phospho-protein content: human HT29 cells treated with RNAi reagent against Polo kinase have an increased percentage of nuclei with high phospho-H3 staining compared to wild-type cells, consistent with a mitosis-stalled phenotype (left). Wild-type human HT29 nuclei that stain positively for phospho-histone H3 tend to be smaller than phospho-H3-negative cells (right). **(f) **Protein localization: the mean intensity of NFκB staining in the cytoplasm and the nucleus is shown in response to TNFα in human MCF7 cells (top). Totals do not equal 100% due to slight overlap between compartments. **(g) **Speckles: fluorescent foci of phospho-Histone2AX induced by 2 Gy of irradiation in human U2OS cells disappear at timepoints as the cells recover. Scale bar = 10 μm. The SEM is too small to show error bars.

**Figure 3:**
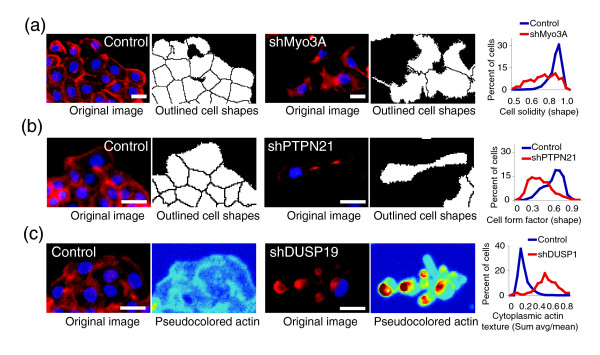
Identifying mutant shapes and textures. In each case, four images of each sample were quantitatively analyzed and images were adjusted using Adobe Photoshop auto levels for display only. Scale bars = 15 μm. **(a) **The unusual cell shape induced by an RNAi reagent against Myo3A in human HT29 cells is quantitatively distinguishable from wild-type control cells. **(b) **The unusual cell shape induced by an RNAi reagent against PTPN21 in human HT29 cells is quantitatively distinguishable from wild-type control cells. **(c) **The unusual actin texture induced by an RNAi reagent against DUSP19 in human HT29 cells is quantitatively distinguishable from wild-type control cells. The images are pseudocolored to show the actin staining texture. The biological basis of these morphological changes and the specificity of the RNAi reagents remain to be determined.

We first demonstrated that CellProfiler's methods could accurately measure many different biologically important features of cells using several cell types, including *Drosophila *Kc167 cells because these cells are particularly challenging to identify by automated image analysis [5,27], and they enable rapid genome-wide screens using living cell microarrays [26]. Using the basic cell-culture methods described previously [26], we prepared *Drosophila *Kc167 cells for experiments shown in Figures 1 and 2 by pretreating the cells with double-stranded RNA (dsRNA) against the noted genes for two days prior to plating on plain glass slides and growing for a further 3 days in the presence of dsRNA. Specifically, 50 μg dsRNA plus 30 μl fugene in 1 ml serum-free medium were transfected into a 10 cm plate containing 20 million cells in 10 ml medium. We prepared human HT29 cells (Figures 1, 2, 3) as previously described [1].

Direct comparison of image analysis software is difficult because results from image analysis can be heavily skewed by how the software is tuned and commercial software packages are numerous and expensive. Furthermore, the algorithms in commercial software are proprietary and so cannot be directly compared apart from the entire software package, including preprocessing methods. The best practical comparison, therefore, is for software developers to release the results of their software on standard image sets or versus gold standards (visual inspection, Coulter particle counters, and so on). In subsequent sections, therefore, we present such comparisons. Note that once validated, any of these experiments could be expanded to a large-scale genome-wide RNAi screen or chemical library screen.

Cell count (used to probe cell proliferation/apoptosis/death) is a straightforward phenotype that has, nonetheless, proved challenging for many cell types due to the poor ability of existing software to separate clumped nuclei. For human cells (Figure 2a, left), CellProfiler's accuracy compared to manual counting is twice that reported for a commercial software package [25]. CellProfiler also counted the more difficult-to-identify *Drosophila *Kc167 cells (Figure 2a, right). Cell size was not previously measurable for many cell types, but CellProfiler's measurements were consistent with the gold standard, a Coulter particle size counter (Figure 2b). While an automated routine has been developed for this cell type [45], this is, to our knowledge, the first report on the quantitative accuracy of any software to count and measure cell size in *Drosophila *Kc167 cells and the results indicate that such screens are now feasible.

Cell count and size can at least be observed by eye even if quantitative high throughput screening is impractical. In contrast, certain phenotypes, like changes in DNA content, are impossible to discern by eye. Unlike whole population-based methods, image cytometry measures individual cell fluorescence intensities so that the DNA content of DNA-stained cells can be determined [46,47]. These measurements are very easily degraded by anomalies in the illumination of the field of view and poor identification algorithms (the most common errors are counting two nearby nuclei as one nucleus with twice the DNA content and incorrectly splitting a nucleus into two half-nuclei). This is, therefore, a very demanding phenotype to measure from images.

Image analysis with CellProfiler produced the expected DNA content distributions for both human and *Drosophila *cell populations (Figure 2c). As another test, we confirmed that the green fluorescent protein (GFP)-histone content per cell decreases by roughly half when a cell divides into two daughter cells during mitosis (Figure 2d). Classification of cells based on 2N, 4N, and 8N DNA content is, therefore, possible based on an image of DNA-stained nuclei (Additional data file 6). This is useful not only in studies of cell cycle *per se*: cell cycle stage is a known cause of variability in biological samples, so analyzing a phenotype of interest with respect to the cell cycle eliminates a confounding variable (for example, the phenotype of interest could be assessed only in G1 phase cells, which have 2N DNA content).

Further, image cytometry adds an additional level of information about cell cycle distribution. Whereas flow cytometry based on a DNA stain alone cannot distinguish cells in G2 and M phase (both having the same 4N DNA content), image cytometry reveals that these two populations differ in that mitotic cells have smaller nuclei on average (Figure 2e, right).

The total amount of a protein or phospho-protein per cell can be measured by analysis of fluorescent antibody staining (Figure 2e, left), amounting to single-cell western blots. Furthermore, image cytometry can determine the localization of staining relative to other labeled cellular compartments. The change in localization of the nuclear factor (NF)κB transcription factor in response to tumor necrosis factor (TNF)α in MCF7 cells can be monitored (Figure 2f). We have previously used the software to confirm the localization of a protein predominantly at the membrane [48]. The software can also identify, count, and measure the shape, size, and intensity of subcellular structures such as nuclear speckles (Figure 2g).

Finally, image analysis can probe other phenotypes that are not otherwise easily measured, such as shape and texture/smoothness. Cell morphology has not often been quantitatively measured, despite its importance in normal cellular physiology and in disease diagnosis [6,17,19,49]. Many of the shape and texture measurements for wild-type cells show non-Gaussian distributions (Additional data file 7). Therefore, independently measuring every cell by image analysis is particularly valuable because the population cannot be accurately described by reduction to a few parameters like mean and standard deviation. We found that changes in cell shape and actin texture induced by gene-specific RNAi were measurable (Figure 3), opening up the possibility for high-throughput screens for these and other morphologies.

## Cytological profiling to reveal pathways targeted by drugs

**Figure 4:**
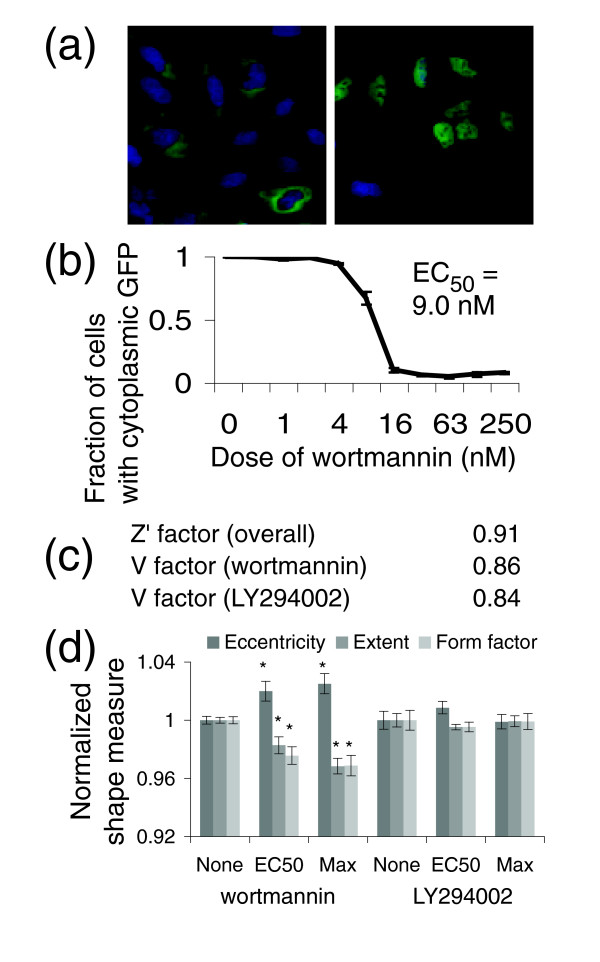
CellProfiler analysis of a Forkhead (FOXO1A) cytoplasm-nucleus translocation assay. **(a) **Example images from the high throughput image set in human U2OS osteosarcoma cells, showing no treatment (left) and 150 nM Wortmannin (right) after 1 hour treatment, scale unknown. **(b) **Translocation scored as the fraction of cells whose ratio of GFP in the cytoplasm versus the nucleus was above a threshold. Error bars = SEM. **(c) **Statistical analysis using Z' and non-logistic-fit V factors, which are standard measures of assay quality (>0.4 is considered screenable and 1 is an ideal assay) [63-65]. **(d) **Nuclei change shape in response to wortmannin but not LY294002, as judged by three shape features. Error bars = SEM; *p < 0.05.

Having demonstrated CellProfiler's ability to measure a large number of relevant phenotypes, we applied it to a publicly available dose-response image set of a Forkhead-EGFP cytoplasm to nucleus translocation assay in human cells grown in multi-well plates (Figure 4a). First, we ran a CellProfiler pipeline (Additional data file 2, part E) to calculate an illumination correction function for each of the five slides and each of the two channels (<10 minutes processing time per slide on a single computer). We then used another CellProfiler pipeline (Additional data file 2, part A) to load each image, correct its illumination using the pre-calculated functions, identify nuclei, identify cell edges, and use the nucleus and cell outlines to define the cytoplasmic region of each cell, thereby defining three compartments for each cell: nucleus, cell, and cytoplasm. For each slide, we tested the pipeline on several random test images and fine-tuned settings in the identification modules as needed. Modules in the pipeline were included to measure: multiple features describing the area and shape of each compartment for each cell; multiple features describing the intensity and texture of each channel within each compartment, including several scales of texture; and the overall intensity, the percent saturation and the amount of blur for the entire image, for quality control purposes. The analysis was run on a desktop computer at a rate of >1 image/minute.

The translocation was easily quantified by many features (Figure 4b; Additional data file 8), the best of which achieved the highest published scores for assay quality yet (Figure 4c), indicating this software's improved ability to identify samples of interest in screens versus algorithms in commercial software [50,51].

Existing commercial software for this assay typically measures translocation only, but we wondered whether the broad spectrum of measurements recorded by CellProfiler (the cytological profiles) could reveal further insights. We noticed that certain features of nuclear shape change in response to increasing doses of wortmannin but, interestingly, not the other positive control drug in this assay, LY294002 (Figure 4d). These subtle changes were seen at all doses at and above the EC50 of wortmannin but at none of the doses of LY294002, even those that clearly are sufficient for translocation of Forkhead, the main readout of this screen. Because wortmannin and LY294002 target an overlapping set of proteins, this result indicates that using this software in a primary screening assay would allow classification of any positively scoring samples as being wortmannin-specific or not, immediately narrowing down the potential pathways involved. While billions of samples have been scored using translocation assays, this is, to our knowledge, the first report of the ability to sub-classify samples based on morphological changes using primary screening data.

## Cluster computing

We routinely run CellProfiler on large image sets (more than 45,000 four-color images) using a cluster of computers. To do this, we add modules to the end of a pipeline to enable processing batches of images on the cluster and exporting data into a database. We then process the first image on a desktop computer, after which CellProfiler automatically divides the remainder of the large image set into groups and creates the files needed to submit each group to a computing cluster. We then use simple commands, outside of CellProfiler, to submit the jobs to our cluster of computers and export the resulting measurements to a database. Each of these steps is described in the CellProfiler help for batch processing, and researchers without a computing cluster can now rent one remotely and inexpensively. Given that a typical image analysis takes approximately two minutes, a single CPU can process 30 images/hour and a 100 CPU cluster processes 3,000 images/hour. This is a much faster rate than existing image acquisition instruments, such that image analysis is not a bottleneck.

## Broad applicability

Here we have shown that CellProfiler is useful for measuring a number of cell features, including cell count, cell size, cell cycle distribution, organelle number and size, cell shape, texture, and the levels and localization of proteins and phospho-proteins. Unlike previously existing software, CellProfiler is effective in a number of cell types and organisms, such that new avenues of research in both standard and high-throughput biology laboratories can now be pursued.

CellProfiler is already being used by laboratories worldwide studying a variety of biological processes in other cell types and organisms, including *Drosophila *(S2R+ cells, epithelial tissue), human (TOV21G, biopsied prostate gland tissue, adult mesenchymal stem cells, H1299 lung carcinoma), mouse (NIH/3T3, neural precursor cells derived from embryos, lung tissue sections, isolated germ cells), and rat (H9c2 cells) [1,26,48,52-54] (KA Hartwell, personal communication). We have also modified CellProfiler to measure yeast colonies, yeast growth patches, wounds in scratch assays, and tumors [55].

Importantly, the only successfully completed *Drosophila *screen using automated image analysis, to date, has been a cell-count/object-count screen in the S2 line whose appearance is comparable to human cell lines [56]. We are currently using CellProfiler to analyze screens using the clumpy *Drosophila *Kc167 cell type (AEC, TRJ, MRL, DB Wheeler, PG, DMS, unpublished data). Given the power of RNA interference and genetic tools in *Drosophila *and the demand for screening in its community [57], this is an area that can now move past tedious visual analysis of thousands of images, accelerating the rate of discovery.

## Future development

We hope that computer vision researchers will contribute new algorithms to the project so that their theoretical work can be applied to practical biological problems. For example, while CellProfiler can currently analyze each slice of a time-lapse movie or three-dimensional image set independently, implementation of algorithms specifically designed to take advantage of the extra context information present in this type of data would be necessary for most experiments using these image types. Furthermore, CellProfiler is currently being integrated with the open-source Open Microscopy Environment project (OME) [58], which would provide a complete open-source infrastructure for organizing and analyzing images from high-throughput experiments.

With the successful application of sophisticated image analysis methods, the bottleneck of image-based genome-wide screens is now moving downstream to data visualization, exploration, and statistical analysis in order to accommodate the number and richness of measurements that result from image-based genome-wide assays [32]. Fully exploiting these rich data sets will reveal cellular signaling networks and lead to the unprecedented rich annotation of hundreds of genes in parallel.

## Additional data files

The following additional data are available with the online version of this paper. Additional data file 1 is the CellProfiler manual. Additional data file 2 shows the CellProfiler pipelines for experiments shown in this paper, listing the modules in the order used. Additional data file 3 is a table listing CellProfiler modules by category, with their descriptions. Additional data file 4 is a table listing the measurements made by CellProfiler modules. Additional data file 5 lists the data and image tools in CellProfiler, with their descriptions. Additional data file 6 is a figure showing an example from CellProfiler analysis of DNA content (cell cycle) in *Drosophila *Kc167 cells. Additional data file 7 is a figure showing histograms of shape and texture features for wild-type cells. Additional data file 8 is a table listing measures for the cytoplasm-nucleus translocation assay (Figure 4) for which the Z' factor is above 0.5.

## Supplementary Material

Additional data file 1 CellProfiler manual Click here for file

Additional data file 2 CellProfiler pipelines for experiments shown in this paper, listing the modules in the order used Click here for file

Additional data file 3 CellProfiler modules by category, with their descriptions Click here for file

Additional data file 4 Measurements made by CellProfiler modules Click here for file

Additional data file 5 Data and image tools in CellProfiler, with their descriptions Click here for file

Additional data file 6 Example from CellProfiler analysis of DNA content (cell cycle) in *Drosophila *Kc167 cells Click here for file

Additional data file 7 Histograms of shape and texture features for wild-type cells Click here for file

Additional data file 8 Measures for the cytoplasm-nucleus translocation assay (Figure 4) for which the Z' factor is above 0.5 Click here for file
