# Supplementary material for: CellProfiler: image analysis software for identifying and quantifying cell phenotypes
Source: Genome Biol. 2006 Oct 31;7(10):R100. doi: 10.1186/gb-2006-7-10-r100 (PMC1794559; doi:10.1186/gb-2006-7-10-r100)
Supplement: Additional data file 1 — CellProfiler manual [file gb-2006-7-10-r100-S1.pdf]

CellProfiler™

cell image analysis software

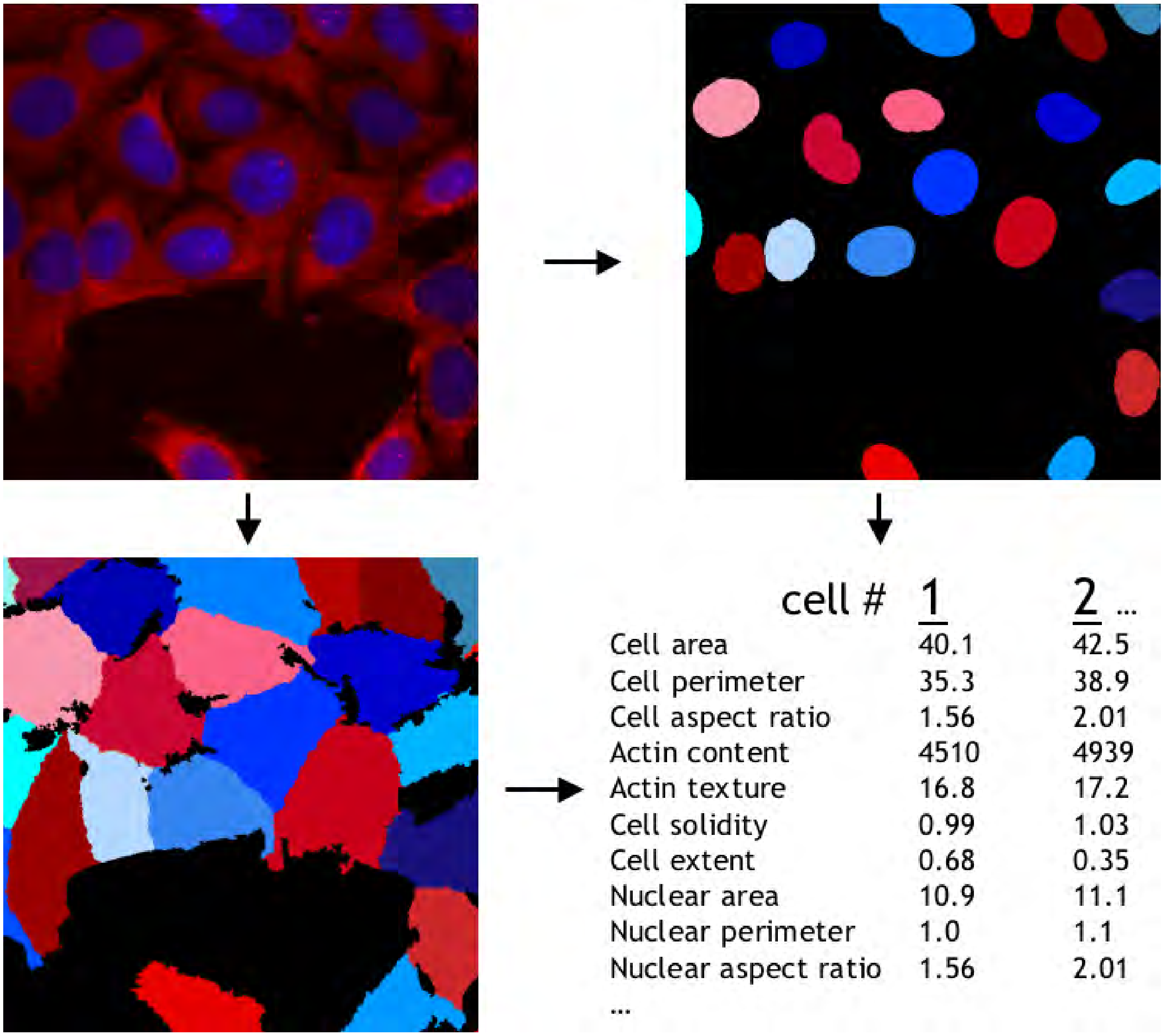

cell #	1	2 …
Cell area	40.1	42.5
Cell perimeter	35.3	38.9
Cell aspect ratio	1.56	2.01
Actin content	4510	4939
Actin texture	16.8	17.2
Cell solidity	0.99	1.03
Cell extent	0.68	0.35
Nuclear area	10.9	11.1
Nuclear perimeter	1.0	1.1
Nuclear aspect ratio	1.56	2.01
…		

CellProfiler™ cell image analysis software

Created by:
Anne E. Carpenter and Thouis Ray Jones

In the laboratories of:
David M. Sabatini and Polina Golland
at:

Whitehead Institute
for Biomedical Research

and

CSAIL
Computer Sciences/ Artificial
Intelligence Laboratory at the
Massachusetts Institute of Technology

Authors (in order of joining the project):
Anne E. Carpenter
Thouis Ray Jones
In Han Kang
Ola Friman
Steve Lowe
Joo Han Chang
Colin Clarke
Mike Lamprecht
Peter Swire
Rodrigo Ipince
Vicky Lay
Jun Liu
Chris Gang

CellProfiler is free and open-source!
Its survival depends on you…

Please credit CellProfiler in publications:

1. cite the website (www.cellprofiler.org)
2. cite the publication (check the website for the citation).
3. post the reference for your publication on the CellProfiler Forum (accessible from the website) so that we are aware of it.

You are also welcome to send donations to the project

Especially if you are working at a company and/or are using this software as an alternative to software costing tens of thousands of dollars…

Contact:

Whitehead Institute Development Office (617-324-7770)
http://wi.mit.edu/support/howtogive.html
We can send your institution an invoice if it is more convenient.

These steps will help us to maintain funding for the project and continue to improve and support it.

Table of Contents

Getting Started:

Introduction . . . 6
Installation . . . 7
Getting Started with CellProfiler . . . 9

Help:

BatchProcessing . . . 11
Colormaps . . . 16
DefaultImageFolder . . . 17
DefaultOutputFolder . . . 18
DeveloperInfo . . . 19
FastMode . . . 28
MatlabCrash . . . 29
OutputFilename . . . 30
PixelSize . . . 31
Preferences . . . 32
SkipErrors . . . 33
TechDiagnosis . . . 34

File Processing modules:

CreateBatchFiles . . . 51
ExportToDatabase . . . 68
ExportToExcel . . . 70
LoadImages . . . 90
LoadSingleImage . . . 94
LoadText . . . 95
RenameOrRenumberFiles . . . 114
Restart . . . 117
SaveImages . . . 119
SplitOrSpliceMovie . . . 124

Image Processing modules:

Align . . . 35
ApplyThreshold . . . 36
Average . . . 37
ColorToGray . . . 43
Combine . . . 44
CorrectIlluminationApply . . . 46
CorrectIlluminationCalculate . . . 47
CorrectIlluminationNew . . . 50
Crop . . . 55
DistinguishPixelLabels . . . 64
FindEdges . . . 72
Flip . . . 74
GrayToColor . . . 75
InvertIntensity . . . 89
MaskImage . . . 96
Morph . . . 110
OverlayOutlines . . . 111
PlaceAdjacent . . . 112
RescaleIntensity . . . 115
Resize . . . 116
Rotate . . . 118
Smooth . . . 122
Subtract . . . 125
SubtractBackground . . . 126
Tile . . . 127

Object Processing modules:

ClassifyObjects . . . 41
ClassifyObjectsByTwoMeasurements . . . 42
ConvertToImage . . . 45
Exclude . . . 65
ExpandOrShrink . . . 66
FilterByObjectMeasurement . . . 71
IdentifyObjectsInGrid . . . 76
IdentifyPrimAutomatic . . . 77
IdentifyPrimManual . . . 83
IdentifySecondary . . . 84
IdentifyTertiarySubregion . . . 88
Relate . . . 113

Measurement modules:

CalculateMath . . . 38
CalculateRatios . . . 39
CalculateStatistics . . . 40
MeasureCorrelation . . . 97
MeasureImageAreaOccupied . . . 98
MeasureImageIntensity . . . 100
MeasureImageSaturationBlur . . . 101
MeasureImageSlope . . . 102
MeasureObjectAreaShape . . . 103
MeasureObjectIntensity . . . 105
MeasureObjectNeighbors . . . 107
MeasureTexture . . . 108

Other modules:

CreateWebPage . . . 53
DefineGrid . . . 58
DisplayDataOnImage . . . 59
DisplayGridInfo . . . 60
DisplayHistogram . . . 61

DisplayImageHistogram 62
DisplayMeasurement 63
SendEmail 121
SpeedUpCellProfiler 123

Image tools:

ImageToolWindow 128
InteractiveZoom 129
OpenNewImageFile 130
ShowHelpForThisMenu 131
ShowOrHidePixelData 132

Data tools:

AddData 133
CalculateRatiosDataTool 134
ClearData 135
ConvertBatchFiles 136
DataLayout 137
ExportData 138
ExportDatabase 139
ExportLocations 140
GenerateHistogramMovie 141
Histogram 142
MeasurementCalculator 145
MergeOutputFiles 146
PlotMeasurement 147
ShowDataOnImage 148
ViewData 149

Introduction

```
CellProfilerTM cell image analysis software

CellProfiler cell image analysis software is designed for biologists
without training in computer vision or programming to quantitatively
measure phenotypes from thousands of images automatically.

CellProfiler Developer's version allows you to write your own modules and
tools for CellProfiler using Matlab.
```

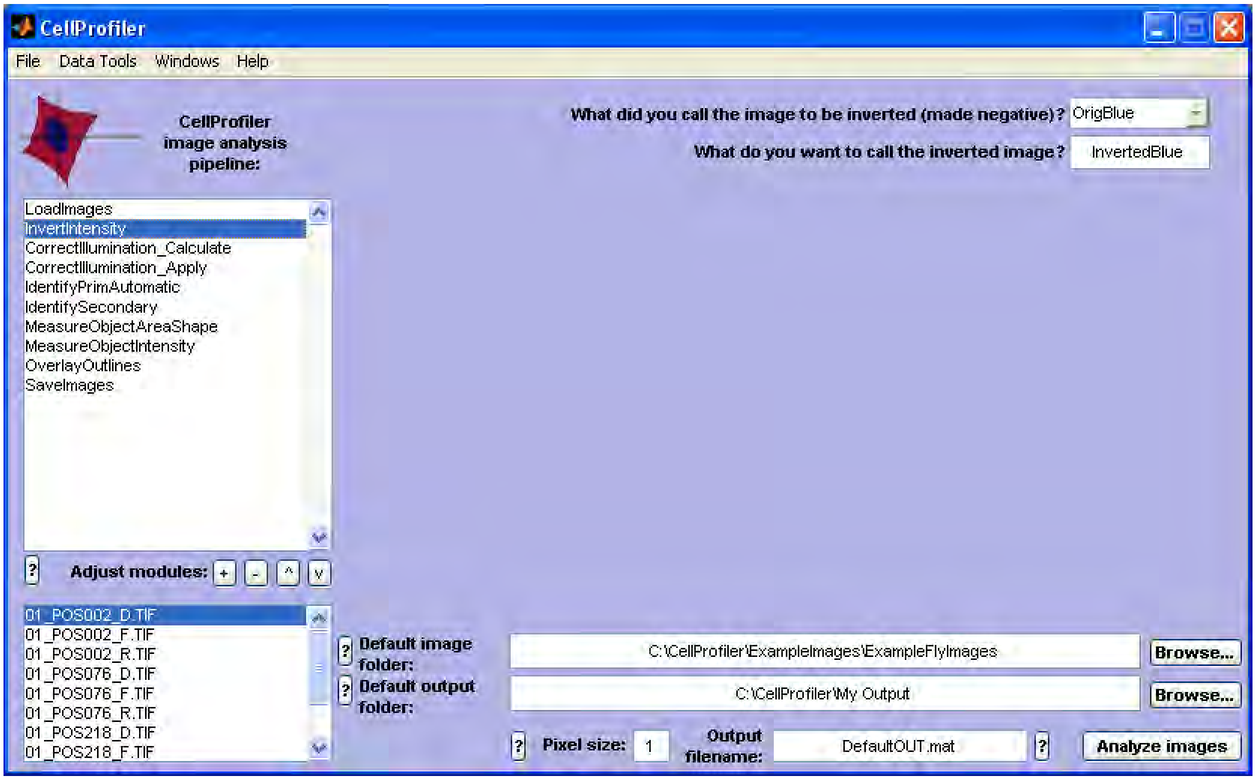

Installation

Get the latest code from www.cellprofiler.org

****

CellProfiler Installation Guide: Macintosh OSX Version

1. Download and unzip CellProfiler to any folder, but all files must remain in the same folder and the folder's name must not have spaces (e.g. /Applications/CellProfiler).

2. Download and install X11.
http://www.apple.com/downloads/macosx/apple/x11formacosx.html

3. To start CellProfiler, double click CellProfiler.command, located in the folder where CellProfiler was un-zipped.

4. (Optional steps) Get up and running quickly with one of the sample pipelines on our Examples page, and check out Help > HelpGettingStarted within CellProfiler. In CellProfiler, set some basic preferences in File > Set preferences, which will make it easier to use CellProfiler. You can drag CellProfiler.command to the dock (on the side near the trash) so the program is easily accessible in the future.

****

CellProfiler Installation Guide: Windows PC Version

1. Download and extract CellProfiler.exe, CellProfiler.ctf, and the Modules directory to any folder, but all files must remain in the same folder (e.g. C:\Program Files\CellProfiler).

2. Download and run MCRInstaller.exe (see link on CellProfiler website).

3. To start CellProfiler, run CellProfiler.exe. The first time the program is run, it will take a bit longer to de-compress the CTF file.

4. (Optional steps) Get up and running quickly with one of the sample pipelines on our Examples page, and check out Help > HelpGettingStarted within CellProfiler. In CellProfiler, set some basic preferences in File > Set preferences, which will make it easier to use CellProfiler. On many PC's, it is beneficial to set the font size to 8 instead of 10.

****

CellProfiler Installation Guide: Developer's version

1. (Mac only) Set X11 setting... Close MATLAB & X11. Open the Mac application called 'Terminal' (Applications > Utilities > Terminal) and type the following to ensure that display windows behave properly:
defaults write com.apple.x11 wm_click_through -bool true

2. Start MATLAB and check licenses... Start MATLAB and type into the main window (command line): license('test','image_toolbox') If the Image Processing Toolbox is installed with a valid license you will see: ans = 1, but if ans = 0, the toolbox and/or license is not installed. CellProfiler was designed for MATLAB Release 14, version 7.1, SP3 although it is possible that many functions will work on other versions. We have noticed lots of MATLAB crashing with Mac OS10.2 but OS10.3 is fine.

3. Copy the CellProfiler files to your computer... Put them in a folder named CellProfiler at any location - suggested: On Mac: /Applications/CellProfiler On PC: C:\Program Files Once the files are copied, do not move or rename this folder or any folders containing it, or you will have to repeat the next step.

4. Start CellProfiler... To start CellProfiler for the first time, you must call it directly. After the first time, you may simply type "CellProfiler" in the main MATLAB window to start CellProfiler. To call CellProfiler directly, type the following in the main MATLAB window (LOCATION is where the CellProfiler folder is located): On Mac: run '/LOCATION/CellProfiler/CellProfiler.m' For example: run '/Applications/CellProfiler/CellProfiler.m'

On PC: run '\LOCATION\CellProfiler\CellProfiler.m' For example: run 'C:\Program Files\CellProfiler\CellProfiler.m'

5. (Optional steps) Get up and running quickly with one of the sample pipelines on our Examples page, and check out Help > HelpGettingStarted within CellProfiler. In CellProfiler, set some basic preferences in File > Set preferences, which will make it easier to use CellProfiler.

Getting Started with CellProfiler

The best way to learn how to use CellProfiler is to load an example pipeline (from www.cellprofiler.org) and try it out. Or, you can build a pipeline from scratch. A pipeline is a sequential set of individual image analysis modules. See also Help (main menu of CellProfiler) and "?" buttons in the main window.
To learn how to program in CellProfiler, see Help > DeveloperInfo.
To learn how to use a cluster of computers to process large batches of images, see Help > BatchProcessing.

************************   LOADING A PIPELINE   ************************

STEP 1: Put the images and pipeline into a folder on your computer.

STEP 2: Set the default image and output folders (lower right of the main window) to be the folder where you put the images.

STEP 3: Load the pipeline using File > Load Pipeline in the main menu of CellProfiler.

STEP 4: Click "Analyze images" to start processing.

STEP 5: Examine the measurements using Data Tools.
Data Tools are accessible in the main menu of CellProfiler and allow you to plot, view, or export your measurements (e.g. to Excel).

STEP 6: If you modify the modules or settings in the pipeline, you can save the pipeline using File > Save Pipeline. See the end of this document for more information on pipeline files.

*****************   BUILDING A PIPELINE FROM SCRATCH   *****************

STEP 1: Place modules in a new pipeline.
Choose image analysis modules to add to your analysis routine (your "pipeline") by clicking '+'. Typically, the first module which must be run is the Load Images module, where you specify the identity of the images that you want to analyze. Modules are added to the end of the pipeline, but their order can be adjusted in the main window by selecting module(s) and using the Move up '^' and Move down 'v' buttons. The '-' button will delete selected module(s) from the pipeline.

Most pipelines depend on a major step: Identifying objects. In CellProfiler, the objects you identify are called Primary, Secondary, or Tertiary. What does this mean? Identify Primary modules identify objects without relying on any information other than a single grayscale input image (e.g. nuclei are typically primary objects). Identify Secondary modules require a grayscale image plus an image where primary objects have already been identified, because the secondary objects' locations are determined in part based on the primary objects (e.g. cells can be secondary objects). Identify Tertiary modules require images where two sets of objects have already been identified (e.g. nuclei and cell regions are used to define the cytoplasm objects, which are tertiary

objects).

Saving images in your pipeline: Due to the typically high number of intermediate images produced during processing, images produced during processing are not saved to the hard drive unless you specifically request it, using a Save Images module.

STEP 2: Adjust the settings in each module.
Back in the main window of CellProfiler, click a module in the pipeline to see its settings in the main workspace. To learn more about the settings for each module, select the module in the pipeline and click the "?" button below the pipeline.

STEP 3: Set the default image folder, default output folder, pixel size, and output filename.
For more help, click their nearby "?" buttons in the main window.

STEP 4: Click "Analyze images" to start processing.
All of the images in the selected folder(s) will be analyzed using the modules and settings you have specified. You will have the option to cancel at any time. At the end of each cycle, the data are saved in the output file.

STEP 5: Examine your measurements using Data Tools.
Data Tools are accessible in the main menu of CellProfiler and allow you to plot, view, or export your measurements (e.g. to Excel).

Note: You can test an analysis on a single image cycle by setting the Load Images module appropriately. For example, if loading by order, you can set the number of images per set to equal the total number of images in the folder (even if it is thousands) so that only the first cycle will be analyzed. Or, if loading by text, you can make the identifying text specific enough that it will recognize only one group of images in the folder. Once the settings look good for a few test images, you can change the Load Images module to recognize all images in your folder.

STEP 6: Save your pipeline.
This step can be done at any time using File > Save Pipeline.

Note about CellProfiler "PIPE" pipeline files: A pipeline can be loaded from a pipeline file or from any output file created using the pipeline. A pipeline file is very small and is therefore more convenient for sharing with colleagues. It also allows you to save your work on a pipeline even if it's not ready to run yet. Loading/Saving Pipeline files will load/save these: the image analysis modules, their settings, and the pixel size. It will not save the default image or output folder.

CellProfiler Help: BatchProcessing

CellProfiler is designed to analyze images in a high-throughput manner. Once a pipeline has been established for a set of images, CellProfiler can export batches of images to be analyzed on a cluster with the pipeline. We often analyze 40,000-130,000 images for one analysis in this manner. This is accomplished by breaking the entire set of images into separate batches, and then submitting each of these batches as individual jobs to a cluster. Each individual batch can be separately analyzed from the rest.

There are two methods of processing these batches on a cluster. The first requires a MATLAB license for every computing node of the cluster. This method produces small MATLAB script files which specify the images to analyze. The other method does not require MATLAB licenses for the entire cluster, but does require a bit more effort to set up. This method produces small MATLAB .mat files which specify the images to analyze.

*************** SETTING UP CLUSTER WITH MATLAB **************************

Step 1: Create a folder on your cluster for CellProfiler (e.g. /home/username/CellProfiler). This folder must be connected to the cluster computers' network and readable by all. If you don't know what this means, please ask your IT department for help.

Step 2: Copy all CellProfiler source code files into this folder, keeping the file structure intact. This version of CellProfiler must be the same as the version used to create the pipeline. Sometimes modules are changed between versions and this can cause errors.

Step 3: Create batchrun.sh file. This file will allow you to rapidly submit jobs to your cluster, rather than you typing out commands one at a time to submit jobs individually. There are different software programs which control how jobs are submitted to a cluster. The example below is for our cluster at the Whitehead Institute which uses LSF software. Contact your IT department for help writing a similar file to work with your own cluster.

Example (LSF): Open any text editor and copy in the code below, then save the file to any directory, usually your home directory is fine. Then change the following lines to fit your cluster:

MATLAB=/SOME_PATH/MATLAB

LICENSE_SERVER="12345@yourservers.edu"

Also, you can specify your e-mail address after the bsub command.

Note that in the example script below, we had to wrap some lines (marked with >>>>>: you should remove the >>> symbols and wrap the line with the previous.

```
#!/bin/sh
if test $# -ne 5; then
  echo "usage: $0 M_fileDir BatchTxtOutputDir mat_fileDir BatchFilePrefix
>>>>>  QueueType" 1>&2 exit 1
fi
```

```
# start to process
BATCHDIR=$1
BATCHTXTOUTPUTDIR=$2
BATCHMATOUTPUTDIR=$3
BATCHFILEPREFIX=$4
QueueType=$5
MATLAB=/SOME_PATH/MATLAB
LICENSE_SERVER="12345@yourservers.edu"
export DISPLAY=""
# loop through each .mat file
for i in $BATCHDIR/$BATCHFILEPREFIX*.m; do
   BATCHFILENAME=`basename $i .m`
    if [ ! -e $BATCHMATOUTPUTDIR/${BATCHFILENAME}_OUT.mat ]; then
      echo Re-running $BATCHDIR/$BATCHFILENAME
      bsub -q $5 -o $BATCHTXTOUTPUTDIR/$BATCHFILENAME.txt -u
      username@wi.mit.edu -R 'rusage [img_kit=1:duration=1]'
>>>>>"$MATLAB/bin/matlab -nodisplay -nojvm -c $LICENSE_SERVER <
>>>>>$BATCHDIR/$BATCHFILENAME.m"
   fi
done
#INSTRUCTIONS
#From the command line, logged into your cluster submit the jobs using
#this script as follows:
#./batchrun.sh /FOLDERWHEREMFILESARE /FOLDERWHERETEXTLOGSSHOULDGO
>>>>>/FOLDERWHEREMATFILESARE BATCHPREFIXNAME QueueType
#Note that FOLDERWHEREMATFILESARE is usually the same as
#FOLDERWHEREMFILESARE. This is mainly if you are trying to re-run failed
#jobs - it only runs m files if there is no corresponding mat file
#located in the FOLDERWHEREMATFILESARE.   For example:
#./batchrun.sh /nfs/sabatini2_ata/PROJECTFOLDER
>>>>>/nfs/sabatini2_ata/PROJECTFOLDER /nfs/sabatini2_ata/
>>>>>PROJECTFOLDER Batch_ normal
# END COPY
---
```

Step 4: Change batchrun.sh to be executable. Open a terminal, navigate to the folder where batchrun.sh is located, and type:

```
chmod a+x batchrun.sh
```

If you don't know what this means, please ask your IT department.

Step 5: Submit files. See SUBMITTING FILES FOR BATCH PROCESSING below.

*********** END OF SETTING UP CLUSTER WITH MATLAB ***********************

************** SETTING UP CLUSTER WITHOUT MATLAB ************************
Step 1: Download and install correct version of CPCluster from www.cellprofiler.org. If the versions there do not work, it means your cluster is running different versions of the operating systems, so you will have to download the CPCluster source code and compile it specifically for your cluster. This requires a single MATLAB license. For more instructions on accomplishing this, see the instructions that are

downloaded with the CPCluster source code.

Step 2: Create batchrun.sh file. This file will allow you to rapidly submit jobs to your cluster, rather than you typing out commands one at a time to submit jobs individually. There are different software programs which control how jobs are submitted to a cluster. The example below is for our cluster at the Whitehead Institute which uses LSF software. Contact your IT department for help writing a similar file to work with your own cluster.

Example (LSF): Open any text editor and copy in the code below, then save the file to any directory, usually your home directory is fine. Then change the following lines to fit your cluster:
CPCluster=/Users/username/CPCluster
Also, you can specify your e-mail address after the qsub command.

Note that in the example script below, we had to wrap some lines (marked with >>>>>: you should remove the >>> symbols and wrap the line with the previous.

```
#!/bin/sh
if test $# -ne 5; then
    echo "usage: $0 M_fileDir BatchTxtOutputDir mat_fileDir
>>>>>BatchFilePrefix QueueType" 1>&2
    exit 1
fi

BATCHDIR=$1
BATCHTXTOUTPUTDIR=$2
BATCHMATOUTPUTDIR=$3
BATCHFILEPREFIX=$4
QueueType=$5

echo $BATCHDIR
echo $BATCHTXTOUTPUTDIR
echo $BATCHMATOUTPUTDIR
echo $BATCHFILEPREFIX
echo $QueueType

CPCluster=/Users/username/CPCluster

for i in $BATCHDIR/$BATCHFILEPREFIX*.mat; do
    BATCHFILENAME=`basename $i .mat`
    if [ $BATCHFILENAME != ${BATCHFILEPREFIX}data ]; then
        if [ ! -e $BATCHMATOUTPUTDIR/${BATCHFILENAME}_OUT.mat ]; then
            echo Running $BATCHDIR/$BATCHFILENAME
            qsub -S /bin/bash -o $BATCHTXTOUTPUTDIR/$BATCHFILENAME.txt -M
>>>>>username@wi.mit.edu $CPCluster/CPCluster.command
>>>>>$BATCHDIR/${BATCHFILEPREFIX}data.mat $BATCHDIR/$BATCHFILENAME.mat
        fi
    fi
done
---
```

Step 3: Change batchrun.sh to be executable. Open a terminal, navigate to the folder where batchrun.sh is located, and type:

```
chmod a+x batchrun.sh
```

If you don't know what this means, please ask your IT department.

Step 4: Submit files. See SUBMITTING FILES FOR BATCH PROCESSING below.

********** END OF SETTING UP CLUSTER WITHOUT MATLAB ********************

************ SUBMITTING FILES FOR BATCH PROCESSING *********************

Note that in several steps below, we had to wrap some lines (marked with >>>>>: you should remove the >>> symbols and wrap the line with the previous.

Step 1:
Create a project folder on your cluster. For high throughput analysis, it is a good idea to create a separate project folder for each run. In general, we like to name our folders with the following convention: 200X_MM_DD_ProjectName. Within this folder, we usually create an "images" folder and an "output" folder. We then transfer all of our images to the images folder. The output folder is where all of your data will be stored. These folders must be connected to the cluster computers network and the output folder must be writeable by everyone (or at least your cluster) because each of the separate cluster computers will write output files in this folder. If you don't know what this means, ask your IT department for help. Within the CellProfiler window, set the appropriate project folders to be the default image and output folders.

Step 2: Create a pipeline for your image set, testing it on a few example images from your image set. In this process, you must be careful to consider the worst case scenarios in your images. For instance, some images may contain no cells. If this happens, the automatic thresholding algorithms will incorrectly choose a very low threshold, and therefore 'find' objects which don't exist. This can be overcome by setting a 'minimum' threshold in IdentifyPrimAutomatic module.

Step 3: Add the module CreateBatchFiles to the end of your pipeline. Please refer to the help for this module to choose the correct settings. If you are processing large batches of images, you may also consider adding ExportToDatabase to your pipeline, before the CreateBatchFiles module. This module will export your data into comma separated values format (CSV), and also create a script to import your data into Oracle or MySQL databases.

Step 4: Click the Analyze images button. The analysis will begin locally processing the first image set. Do not be surprised if processing the first image set takes much longer than usual. The first step is to check all the images that are present in the images folder - they are not opened or loaded, but just checking the presence of all the files takes a while. At the end of processing the first cycle locally, the CreateBatchFiles module causes local processing to stop and it then

creates the proper batch files and saves them in the default output folder (Step 1). It will also save the necessary data file, which is called XXX_data.mat. You are now ready to submit these batch files to the cluster to run each of the batches of images on different computers on the cluster.

Step 5: Log on to your cluster, and navigate to the directory where you have saved the batchrun.sh file (See "Setting Up Cluster For CellProfiler"). The usage of batchrun.sh is as follows:

```
 ./batchrun.sh M_fileDir BatchTxtOutputDir mat_fileDir BatchFilePrefix
>>>>>QueueType
```

where M_fileDir is the location of the batch files created by CreateBatchFiles in step 4, BatchTxtOutputDir is where you want to store the txt files which have the output of MATLAB during the analysis (includes information like errors and run times), mat_fileDir is the folder where XXX_data.mat is located (this file is created in Step 4), BatchFilePrefix is the prefix named in CreateBatchFiles (usually Batch_), and QueueType is the queue for your cluster. Usually, the first three arguments are all the same. Here is an example of how you would submit all of your batch files to the cluster:

```
 ./batchrun.sh /Some_Path/200X_XX_XX_ProjectName/output
>>>>>/Some_Path/200X_XX_XX_ProjectName/output
>>>>>/Some_Path/200X_XX_XX_ProjectName/output Batch_ normal
```

In this case, the output folder contains the script files, the XXX_data.mat file, and is the folder where we want the txt files with the MATLAB output to be written. The prefix is Batch_ so XXX_data.mat is actually Batch_data.mat. The queue type is normal, but this is specific to your cluster. Ask your IT department what queues are available for your use.

Once all the jobs are submitted, the cluster will run each script individually and produce a separate output file containing the data for that batch of images in the output directory. Then you can decide how to access your data. In general, data from large analyses will be loaded into a database. Please refer to the ExportToDatabase module for information on how to do this. If you have made a very small number of measurements, you might be able to use the MergeOutputFiles DataTool, see its instructions for further details.

If the batch processing fails for some reason, the handles structure in the output file will have a field BatchError, and the error will also be written to standard out. Check the output from the batch processes to make sure all batches complete. Batches that fail for transient reasons can be resubmitted.

********* END OF SUBMITTING FILES FOR BATCH PROCESSING *****************

CellProfiler Help: Colormaps

Default colormaps can be set in File > Set preferences.

Label colormap - affects how objects are colored. Colorcube (and possibly other colormaps) is not recommended because some intensity values are displayed as black. Jet is the default.

Intensity colormap - affects how grayscale images are displayed. Colorcube (and possibly other colormaps) is not recommended because some intensity values are displayed as black. Gray is recommended.

Choose from these colormaps:
autumn bone colorcube cool copper flag gray hot hsv jet lines pink prism spring summer white winter

CellProfiler Help: DefaultImageFolder

Help for the default image folder, in the main CellProfiler window: Select the main folder containing the images you want to analyze. Use the Browse button to select the folder, or carefully type the full pathname in the box. You can change the folder which is the default image folder upon CellProfiler startup by using File > Set Preferences.

The contents of the folder are shown to the left, which allows you to check file names or look at the order of images from within CellProfiler. Doubleclicking image file names in this list will open them. Doubleclicking on PIPE or OUT files will ask if you want to load a pipeline from the file. To refresh the contents of this window, press enter in the default image directory edit box.

You will have the option within the Load Images module to retrieve images from other folders, but the folder selected here will be the default.

Be careful that files other than your images of interest are not stored within the folder you have selected. The following file extensions are ignored by CellProfiler, so these are the only types which can be left in the folder with the images you want to analyze:

m, m~, frk~, xls, doc, rtf, txt, csv, or any file beginning with a dot.

CellProfiler Developer's version note: If you would like to add a particular file format to this list, first save a copy of the main CellProfiler program (CellProfiler.m) in a separate location as a backup in case you make an error, then go to File > Open and select CellProfiler.m. Find the line that looks like the following and add any extensions:

```
  DiscardsByExtension = regexpi(FileNamesNoDir, '\.(m|mat|m~|frk~|xls|
                                       doc|rtf|txt|csv)$', 'once');
```

Save the file. You do not need to relaunch MATLAB or CellProfiler for this change to take effect.

CellProfiler Help: DefaultOutputFolder

Help for the default output folder, in the main CellProfiler window: Select the main folder where you want CellProfiler's output to be saved. Use the Browse button to select the folder, or carefully type the full pathname in the box. You can change the folder which appears upon CellProfiler startup by using File > Set Preferences.

You will have the option to save output to other locations: for example, the output file can be saved elsewhere by typing a full pathname in the 'Name the output file' box, and many modules (like Save Images) allow you to override the default output folder by entering the pathname in the settings.

CellProfiler Help: DeveloperInfo

Programming Notes for CellProfiler Developer's version

*** INTRODUCTION ***

You can write your own modules, image tools, and data tools for CellProfiler - the easiest way is to modify an existing one. CellProfiler is modular: every module, image tool, and data tool is a single MATLAB m-file (extension = .m). Upon startup, CellProfiler scans its Modules, DataTools, and ImageTools folders looking for files. Simply put your new file in the proper folder and it will appear in the proper place. They are automatically categorized, their help extracted, etc.

If you have never tried computer programming or have not used MATLAB, please do give it a try. Many beginners find this language easy to learn and the code for CellProfiler is heavily documented so that you can understand what each line does. It was designed so that biologists without programming experience could adapt it.

*** HELP SECTIONS AT THE BEGINNING OF EACH MODULE AND TOOL ***

The first unbroken block of lines will be extracted as help by CellProfiler's 'Help for this analysis module' button, Help for image tools and data tools (Help menu in the main CellProfiler window) as well as MATLAB's built in 'help' and 'doc' functions at the command line. It will also be used to automatically generate a pdf manual page for the module. An example image demonstrating the function of the module can also be saved in tif format, using the same name as the module, and it will automatically be included in the pdf manual page as well. Follow the convention of: Help for the XX module, Category (use an exact match of one of the categories so your module appears in the proper place in the "Add module" window), Short description, purpose of the module, description of the settings and acceptable range for each, how it works (technical description), and See also NameOfModule. The license/author information should be separated from the help lines with a blank line so that it does not show up in the help displays.

*** SETTINGS (CALLED 'VARIABLES' IN THE CODE) ***

Variables are automatically extracted from lines in a commented section near the beginning of each module. Even though they look like comments they are critical for the functioning of the code. The syntax here is critical - indenting lines or changing the spaces before and after the equals sign will affect the ability of the variables to be read properly.

* The '%textVAR' lines contain the variable descriptions which are displayed in the CellProfiler main window next to each variable box. This text will wrap appropriately so it can be as long as desired, but it must be kept on a single line in the m-file (do not allow it to wrap).

* Whether the variable is entered into an edit box, chosen from a popup menu, or selected using browse buttons is determined by %inputtypeVAR

lines and the %textVAR lines. The options are:
- edit box (omit any %inputtypeVAR line for that variable number and use a %defaultVAR line to specify what text will appear in the box when the user first loads the module)
- popup menu (use %inputtypeVAR = popupmenu and then use %choiceVAR lines, in the order you want them to appear, for each option that should appear in the popup menu)
- popupmenu custom (this allows the user to choose from choices but also to have the option of typing in a custom entry. Use %inputtypeVAR = popupmenu custom and then use %choiceVAR lines, in the order you want them to appear, for each option that should appear in the popup menu)
- pathname box + browse button (omit the %inputtypeVAR line and instead use %pathnametextVAR - the default shown in the edit box will be a period; this default is currently not alterable)
- filename box + browse button (omit the %inputtypeVAR line and instead use %filenametextVAR - the default shown in the edit box will be the text "NO FILE LOADED"; this default is currently not alterable)

* The %infotypeVAR lines specify the group that a particular entry will belong to. You will notice that many entries that the user types into the main window of CellProfiler are then available in popup menus in other modules. This works by classifying certain types of variable entries as follows:
- imagegroup indep: the user's entry will be added to the imagegroup, and will therefore appear in the list of selectable images for variables whose type is 'imagegroup'. Usually used in combination with an edit box; i.e. no %inputtype line.
- imagegroup: will display the user's image entries. Usually used in combination with a popupmenu.
- objectgroup indep and objectgroup: Same idea as imagegroup, for passing along object names.
- outlinegroup indep and outlinegroup: Same idea as imagegroup, for passing along outline names.
- datagroup indep and datagroup: Same idea as imagegroup, for passing along text/data names.
- gridgroup indep and gridgroup: Same idea as imagegroup, for passing along grid names.

* The line of actual code within each group of variable lines is what actually extracts the value that the user has entered in the main window of CellProfiler (which is stored in the handles structure) and saves it as a variable in the workspace of this module with a meaningful name.

* For CellProfiler to load modules and pipelines correctly, the order of variable information should be as follows:

```
%textVAR01 = Whatever text description you want to appear
%defaultVAR01 = Whatever text you want to appear
(OR, %choiceVAR01 = Whatever text)
%infotypeVAR01 = imagegroup indep
BlaBla = char(handles.Settings.VariableValues{CurrentModuleNum,1});
%inputtypeVAR01 = popupmenu
```

In particular, when the input type is "popupmenu custom", the choiceVAR01 line should be after textVAR01. This order is necessary because the textVAR01 creates a VariableBox associated with a variable

number. Also, the defaultVAR01 value will inadvertently overwrite saved settings when loading a saved pipeline if it is located after infotypeVAR01 or inputtypeVAR01.

* CellProfiler uses VariableRevisionNumbers to help programmers notify users when something significant has changed about the variables. For example, if you have switched the position of two variables, loading a pipeline made with the old version of the module will not behave as expected when using the new version of the module, because the settings (variables) will be mixed up. The line should use this syntax:

```
%%%VariableRevisionNumber = 1
```

If the module does not have this line, the VariableRevisionNumber is assumed to be 0. This number need only be incremented when a change made to the modules will affect a user's previously saved settings. There is a revision number at the end of the license info at the top of the m-file for our source-control revisions - this revision number does not affect the user's previously saved settings files and you can ignore it.

*** STORING AND RETRIEVING DATA: THE HANDLES STRUCTURE ***

In CellProfiler (and MATLAB in general), each independent function (module) has its own workspace and is not able to 'see' variables produced by other modules. For data or images to be shared from one module to the next, they must be saved to what is called the 'handles structure'. This is a variable, whose class is 'structure', and whose name is handles. The contents of the handles structure can be printed out at the command line of MATLAB using the Tech Diagnosis button and typing "handles" (no quotes). The only variables present in the *main* handles structure are handles to figures and GUI elements. Everything else should be saved in one of the following substructures:

handles.Settings:
Everything in handles.Settings is stored when the user uses File > Save pipeline, and these data are loaded into CellProfiler when the user uses File > Load pipeline. This substructure contains all necessary information to re-create a pipeline, including which modules were used (including variable revision numbers), their settings (variables), and the pixel size. Fields currently in handles.Settings: PixelSize, VariableValues, NumbersOfVariables, VariableInfoTypes, VariableRevisionNumbers, ModuleNames, SelectedOption.

*** N.B. handles.Settings.PixelSize is where you should retrieve the PixelSize if needed, not in handles.Preferences!

handles.Pipeline:
This substructure is deleted at the beginning of the analysis run (see 'Which substructures are deleted prior to an analysis run?' below). handles.Pipeline is for storing data which must be retrieved by other modules. This data can be overwritten as each image cycle is processed, or it can be generated once and then retrieved during every subsequent image set's processing, or it can be saved for each image set by saving it according to which image cycle is being analyzed, depending on how it will be used by other modules. Example fields in handles.Pipeline: FileListOrigBlue, PathnameOrigBlue, FilenameOrigBlue, OrigBlue (which

contains the actual image). Whether the handles.Pipeline structure is stored in the output file or not depends on whether you are in Fast Mode (see Help > HelpFastMode or File > SetPreferences).

handles.Current:
This substructure contains information needed for the main CellProfiler window display and for the various modules and help files to function. It does not contain any module-specific data (which is in handles.Pipeline). Example fields in handles.Current: NumberOfModules, StartupDirectory, DefaultOutputDirectory, DefaultImageDirectory, FilenamesInImageDir, CellProfilerPathname, CurrentHandles, ImageToolsFilenames, ImageToolHelp, DataToolsFilenames, DataToolHelp, HelpFilenames, Help, NumberOfImageSets, SetBeingAnalyzed, SaveOutputHowOften, TimeStarted, CurrentModuleNumber, FigureNumberForModuleXX.

handles.Preferences:
Everything in handles.Preferences is stored in the file CellProfilerPreferences.mat when the user uses File > Set Preferences. These preferences are loaded upon launching CellProfiler, or individual preferences files can be loaded using File > Load Preferences. Fields in handles.Preferences: PixelSize, DefaultModuleDirectory, DefaultOutputDirectory, DefaultImageDirectory, IntensityColorMap, LabelColorMap, StripPipeline, SkipErrors, FontSize.

The PixelSize, DefaultImageDirectory, and DefaultOutputDirectory fields can be changed for the current session by the user using edit boxes in the main CellProfiler window, which changes their values in handles.Settings or handles.Current. Therefore:

*** N.B. handles.Settings.PixelSize is where you should retrieve the PixelSize if needed, not in handles.Preferences!

*** N.B. handles.Current.DefaultImageDirectory is where you should retrieve the DefaultImageDirectory if needed, not in handles.Preferences!

*** N.B. handles.Current.DefaultOutputDirectory is where you should retrieve the DefaultOutputDirectory if needed, not in handles.Preferences!

handles.Measurements:
Everything in handles.Measurements contains data specific to each image analyzed and is therefore accessed by the data tools. This substructure is deleted at the beginning of the analysis run (see 'Which substructures are deleted prior to an analysis run?' below).

Note that two types of measurements are typically made: Object and Image measurements. Object measurements have one number for every object in the image (e.g. Object Area) and image measurements have one number for the entire image, which could come from one measurement from the entire image (e.g. Image TotalIntensity), or which could be an aggregate measurement based on individual object measurements (e.g. Image MeanArea). Use the appropriate substructure to ensure that your data will be extracted properly. The relationships between objects can also be defined. For example, a nucleus might be associated with a particular cytoplasm and therefore each nucleus has a cytoplasm's number in the nucleus' measurement field which links the two. Or, for multiple speckles within a nucleus, each speckle will have a nucleus' number indicating which nucleus the speckle belongs to (see the Relate Objects module or Identify Secondary or Tertiary modules). Image measurements include a few

standard fields: ModuleErrorFeatures, ModuleError, TimeElapsed, FileNamesText, FileNames, PathNamesText, PathNames.

The other measurement types have two entries: e.g. handles.Measurements.Nuclei.AreaShapeFeatures and handles.Measurements.Nuclei.AreaShape. The substructure ending in "Features" contains descriptive names of each measurement, e.g. "Area" "Perimeter", "Form Factor". These are essentially column headings. The companion substructure which lacks the "Feature" ending contains the actual numerical measurements themselves. For modules that measure the same objects in different ways (e.g. the Intensity module can measure intensities for Nuclei in two different images, blue and green), the identifying info becomes part of the substructure name, e.g.:
handles.Measurements.Nuclei.Intensity_BlueFeatures
handles.Measurements.Nuclei.Intensity_Blue
handles.Measurements.Nuclei.Intensity_GreenFeatures
handles.Measurements.Nuclei.Intensity_Green

Be sure to consider whether measurements you are storing will overwrite each other if more than one module is placed in the pipeline. You can differentiate measurements by including something specific in the name (e.g. Intensity modules include the image name (e.g. Blue or Green) in the substructure name). There are also several examples of modules where new measures are appended to the end of an existing substructure (i.e. forming a new column). See Calculate Ratios, which calls the CPaddmeasurements subfunction to do this.

Why are file names stored in several places in the handles structure?
The Load Images module creates both handles.Pipeline.FilenameIMAGENAME and handles.Pipeline.FileListIMAGENAME when loading an image or movie. In addition, file names are stored in handles.Measurements.Image.FileNames. They are present in Measurements so that they can be exported properly. For movies, the FileList field has the original name of the movie file and how many frames it contains. The Filenames field has the original movie file name and appends the frame number for every frame in the movie. This allows the names to be used in other modules such as SaveImages, which would otherwise over-write itself on every cycle using the original file name. The FileList location is created at the beginning of the run and contains all the images that will possibly be analyzed, whereas the Filename location is only populated as the images cycle through. We think there are good reasons for having filenames located in different places (especially when dealing with movie files) but we have not documented it thoroughly here!

Which substructures are deleted prior to an analysis run?
Anything stored in handles.Measurements or handles.Pipeline will be deleted at the beginning of the analysis run, whereas anything stored in handles.Settings, handles.Preferences, and handles.Current will be retained from one analysis to the next. It is important to think about which of these data should be deleted at the end of an analysis run because of the way MATLAB saves variables: For example, a user might process 12 image sets of nuclei which results in a set of 12 measurements ("TotalStainedArea") stored in handles.Measurements.Image. In addition, a processed image of nuclei from the last image set is left in handles.Pipeline.SegmentedNuclei. Now, if the user uses a different module which happens to have the same measurement output name

"TotalStainedArea" to analyze 4 image sets, the 4 measurements will overwrite the first 4 measurements of the previous analysis, but the remaining 8 measurements will still be present. So, the user will end up with 12 measurements from the 4 sets. Another potential problem is that if, in the second analysis run, the user runs only a module which depends on the output "SegmentedNuclei" but does not run a module that produces an image by that name, the module will run just fine: it will just repeatedly use the processed image of nuclei leftover from the last image set, which was left in handles.Pipeline.

*** IMAGE ANALYSIS ***

If you plan to use the same function in two different m-files (e.g. a module and a data tool, or two modules), it is helpful to write a CPsubfunction called by both m-files so that you have only one subfunction's code to maintain if any changes are necessary.

Images loaded into CellProfiler are in the 0 to 1 range for consistency across modules. When retrieving images into your module, you can check the images for proper range, size, color/gray, etc using the CPretrieveimage subfunction.

We have used many MATLAB functions from the image processing toolbox. Currently, CellProfiler does not require any other toolboxes for processing.

The 'drawnow' function allows figure windows to be updated and buttons to be pushed (like the pause, cancel, help, and view buttons). The 'drawnow' function is sprinkled throughout the code so there are plenty of breaks where the figure windows/buttons can be interacted with. This does theoretically slow the computation somewhat, so it might be reasonable to remove most of these lines when running jobs on a cluster where speed is important.

*** ERROR HANDLING ***

* In data tools & image tools:
```
      CPerrordlg(['Image processing was canceled in the ',ModuleName,'
              module because your entry ',ValueX,' was invalid.'])
      return
```

* In modules and CPsubfunctions (no need for "return"):
```
      error('Your error message here.')
```

* Note:
Always try to make the subfunctions as less likely to have errors as possible. Whenever you can, have error checks in the calling function before the subfunction gets called. Since CPsubfunctions use error('message'), you should try to nest any calls to them in a try/catch. Plus, this allows you to add more specific information to the error message (such as where in the calling function did the error occur). To do this, you can just throw an error whose message has your additional information together with lasterr (which retrieves the last error message). In data tools and image tools CPerrordlg('message') and

return is needed because they are usually called independently, and using error('message') would just stop execution, but would not prompt the user with the corresponding error message.

*** DISPLAYING RESULTS ***

Each module checks whether its figure is open before calculating images that are for display only. This is done by examining all the figure handles for one whose handle is equal to the assigned figure number for this algorithm. If the figure is not open, everything between the "if" and "end" is ignored (to speed execution), so do not do any important calculations there. Otherwise an error message will be produced if the user has closed the window but you have attempted to access data that was supposed to be produced by this part of the code. This is especially problematic when running on a cluster of computers with no displays. If you plan to save images which are normally produced for display only, the corresponding lines should be moved outside this if statement. Also, any additional uicontrol's (popupmenu's, pushbutton's) should be designed using the unit of pixels, since this is standard across platforms unlike other units such as inches and points.

STEP 1: Find the appropriate figure window. If it is closed, usually none of the remaining steps are performed.

```
  ThisModuleFigureNumber = handles.Current.(['FigureNumberForModule',CurrentModule]);
  if any(findobj == ThisModuleFigureNumber)
```

STEP 2: Activate the appropriate figure window so subsequent steps are performed inside this window:

```
  CPfigure(handles,'Image',ThisModuleFigureNumber);
```

For figures that contain any images, choose 'Image', otherwise choose 'Text'. 'Image' figures will have the RGB checkboxes which allow displaying individual channels and they will also have the InteractiveZoom and CellProfiler Image Tools menu items.

STEP 3: (only during starting image cycle) Make the figure the proper size:

```
  if handles.Current.SetBeingAnalyzed == handles.Current.StartingImageSet
    CPresizefigure('','NarrowText',ThisModuleFigureNumber)
  end
```

The figure is adjusted to fit the aspect ratio of the images, depending on how many rows and columns of images should be displayed. The choices are: OneByOne, TwoByOne, TwoByTwo, NarrowText. If a figure display is unnecessary for the module, skip STEP 2 and here use:

```
  if handles.Current.SetBeingAnalyzed == handles.Current.StartingImageSet
    close(ThisModuleFigureNumber)
  end
```

STEP 4: Display your image:

```
  ImageHandle = CPimagesc(Image,handles);
```

This CPimagesc displays the image and also embeds an image tool bar which will appear when you click on the displayed image. The handles are passed in so the user's preferences for font size and colormap are used.

*** DEBUGGING HINTS ***

```
* Use breakpoints in MATLAB to stop your code at certain points and
examine the intermediate results.

* To temporarily show an image during debugging, add lines like this to
your code, or type them at the command line of MATLAB:
      CPfigure
      CPimagesc(BlurredImage, [])
      title('BlurredImage')

* To temporarily save an intermediate image during debugging, try this:
      imwrite(BlurredImage, 'FileName.tif', 'FileFormat');
Note that you may have to alter the format of the image before
saving. If the image is not saved correctly, for example, try
adding the uint8 command:
      imwrite(uint8(BlurredImage), 'FileName.tif', 'FileFormat');

* To routinely save images produced by this module, see the help in
the SaveImages module.

* If you want to save images that are produced by other modules but that
are not given an official name in the settings boxes for that module,
alter the code for the module to save those images to the handles
structure and then use the Save Images module.
The code should look like this:
fieldname = ['SomeDescription(optional)',ImgOrObjNameFromSettingsBox];
handles.Pipeline.(fieldname) = ImageProducedBytheModule;
Example 1:
fieldname = ['Segmented', ObjectName];
handles.Pipeline.(fieldname) = SegmentedObjectImage;
Example 2:
fieldname = CroppedImageName;
handles.Pipeline.(fieldname) = CroppedImage;

For General help files:
We have one line of actual code in these files so that the help is
visible. We are not using CPhelpdlg because using helpdlg instead allows
the help to be accessed from the command line of MATLAB. The one line of
code in each help file (helpdlg) is never run from inside CP anyway.

*** RUNNING CELLPROFILER WITHOUT THE GRAPHICAL USER INTERFACE ***

In order to run CellProfiler modules without the GUI you must have the
following variables:

handles.Settings.ModuleNames (for all modules in pipeline)
handles.Settings.VariableValues (for all modules in pipeline)
handles.Current.CurrentModuleNumber (must be consistent with pipeline)
handles.Current.SetBeingAnalyzed (must be consistent with pipeline)
handles.Current.FigureNumberForModuleXX (for all modules in pipeline)
handles.Current.NumberOfImageSets (set by LoadImages, so if it is run
first, you do not need to set it)
handles.Current.DefaultOutputDirectory
handles.Current.DefaultImageDirectory
```

```
handles.Current.NumberOfModules
handles.Preferences.IntensityColorMap (only used for display purposes)
handles.Preferences.LabelColorMap (only used for display purposes)
handles.Preferences.FontSize (only used for display purposes)
```

You will also need to have the CPsubfunctions folder, since our Modules call CP subfunctions for many tasks. The CurrentModuleNumber needs to be set correctly for each module in the pipeline since this is how the variable values are called. In order to see what all of these variables look like, run a sample analysis and then go to File -> Tech Diagnosis. This will let you manipulate the handles variable in MATLAB.

CellProfiler Help: FastMode

Fast mode can be set in File > Set preferences.

If you uncheck the box you will run in diagnostic mode, where all the intermediate images and calculations for the most recent image cycle are saved in the output file, which drastically increases the output file size. Check the box if you would instead like to run in normal (fast) mode, producing smaller output files.

See also the SpeedUpCellProfiler module.

CellProfiler Help: MatlabCrash

```
Troubleshooting frequent crashes using CellProfiler Developer's version
on the Mac:

If you're running CellProfiler on MATLAB Service Pack 2 (R14SP2) with Mac
OS X 10.4, MATLAB often crashes with Java errors. The only fix is to
upgrade MATLAB to Service Pack 3(R14SP3). For more information about
this, please visit the Mathworks website at:
http://www.mathworks.com/support
```

CellProfiler Help: OutputFilename

```
Naming the output file:
Type in the text you want to use to name the output file, which is where
all of the information about the analysis as well as any measurements are
stored. 'OUT.mat' will be added automatically at the end of whatever you
type in the box. The file will be saved in the default output directory
unless you type a full path and file name into the output file name box.
The path must not have spaces or characters disallowed by your platform.

The program prevents you from entering a name which exists already (when
'OUT.mat' is appended). This prevents overwriting an output data file by
accident, but is also disallowed for the following reason: when a file is
'overwritten', instead of completely overwriting the output file,
MATLAB/CellProfiler just replaces some of the old data with the new data.
So, if you have an output file with 12 measurements and the new set of
data has only 4 measurements, saving the output file to the same name
would produce a file with 12 measurements: the new 4 followed by 8 old
measurements.
```

CellProfiler Help: PixelSize

The pixel size is the number of micrometers per pixel. This is based on the resolution and binning of the camera and the magnification of the objective lens. This number is used to convert measurements to micrometers instead of pixels if you would like the size measurements to be scaled for your images. If instead you want the measurements to be reported in pixels, enter '1' (1 pixel = 1 micrometer) and the measurements will be produced in units of pixels.

The default pixel size can be set in File > Set preferences. Upon startup, the default preferences are loaded or you can load preferences using File > Load Preferences. Either way, the preference for pixel size will be shown in the main window of CellProfiler. You can change the pixel size for the current session by typing it into the main window of CellProfiler. This value is stored in the settings if you File > Save Pipeline or if you create an output file and later File > Load Pipeline from that output file.

CellProfiler Help: Preferences

File > Set Preferences: The default preferences file (CellProfilerPreferences.mat) is loaded upon starting up CellProfiler and is located in the folder where the software is running (or the MATLAB root in CellProfiler Developer's version). If you do not have permission to write files in that location, it saves the file in the current folder (Developer's version), but then the preferences will only be used when CellProfiler is launched from that folder. If you do not have write permission in either location, save them as personal preferences and use File > Load Preferences each time you start CellProfiler.

CellProfiler Help: SkipErrors

```
Skip errors mode can be set in File > Set preferences.

This option will allow you to skip modules which have produced errors. If
a module fails, the pipeline will continue to run. To check if any
modules have failed, use Data Tools -> ExportData and be sure to export
the Image data. In the resulting Image file, there will be one
ModuleError field for each module. If any of these values are above 0,
that means the module failed at some point in the analysis.
```

CellProfiler Help: TechDiagnosis

```
Technical diagnosis mode is available using File > Tech Diagnosis.

This is only to be used in CellProfiler Developer's version.
It allows you to access the workspace of CellProfiler directly at the
command line of MATLAB, including looking into the handles structure.

Type "return" at the command line of MATLAB to exit this mode.
```

Module: Align

```
Help for the Align module:
Category: Image Processing

SHORT DESCRIPTION:
Aligns two or three images relative to each other. Particularly useful to
align microscopy images acquired from different color channels.
*************************************************************************

For two or three input images, this module determines the optimal
alignment among them.  This works whether the images are correlated or
anti-correlated (bright in one = bright in the other, or bright in one =
dim in the other). This is useful when the microscope is not perfectly
calibrated because, for example, proper alignment is necessary for
primary objects to be helpful to identify secondary objects. The images
are cropped appropriately according to this alignment, so the final
images will be smaller than the originals by a few pixels if alignment is
necessary.

Settings:

After entering the names of the images to be aligned as well as the
aligned image name(s), choose whether to display the image produced by
this module by selecting "yes" in the appropriate menu. Lastly, select
the method of alignment. There are two choices, one is based on mutual
information while the other is based on the cross correlation. When using
the cross correlation method, the second image should serve as a template
and be smaller than the first image selected.
```

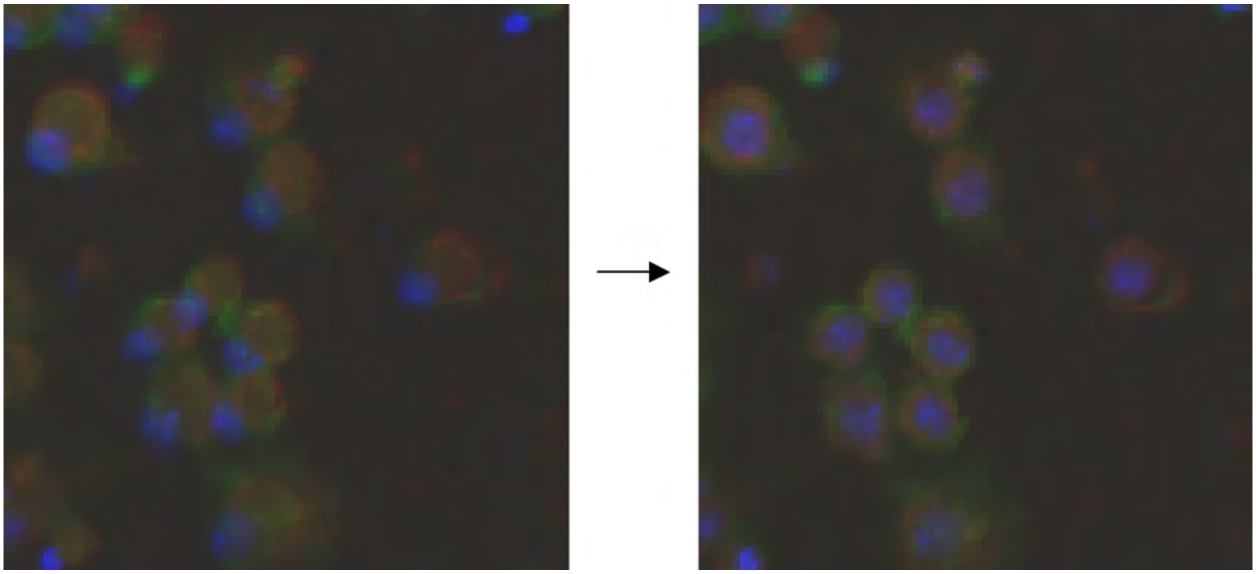

Module: ApplyThreshold

```
Help for the Apply Threshold module:
Category: Image Processing

SHORT DESCRIPTION:
Pixel intensity below or above a certain threshold is set to zero.
*************************************************************************

Settings:

When a pixel is thresholded, its intensity value is set to zero so that
it appears black.

If you wish to threshold dim pixels, change the value for which "Pixels
below this value will be set to zero". In this case, the remaining pixels
can retain their original intensity values or are shifted dimmer to
match the threshold used.

If you wish to threshold bright pixels, change the value for which
"Pixels above this value will be set to zero". In this case, you can
expand the thresholding around them by entering the number of pixels to
expand here: This setting is useful to adjust when you are attempting to
exclude bright artifactual objects: you can first set the threshold to
exclude these bright objects, but it may also be desirable to expand the
thresholded region around those bright objects by a certain distance so
as to avoid a 'halo' effect.
```

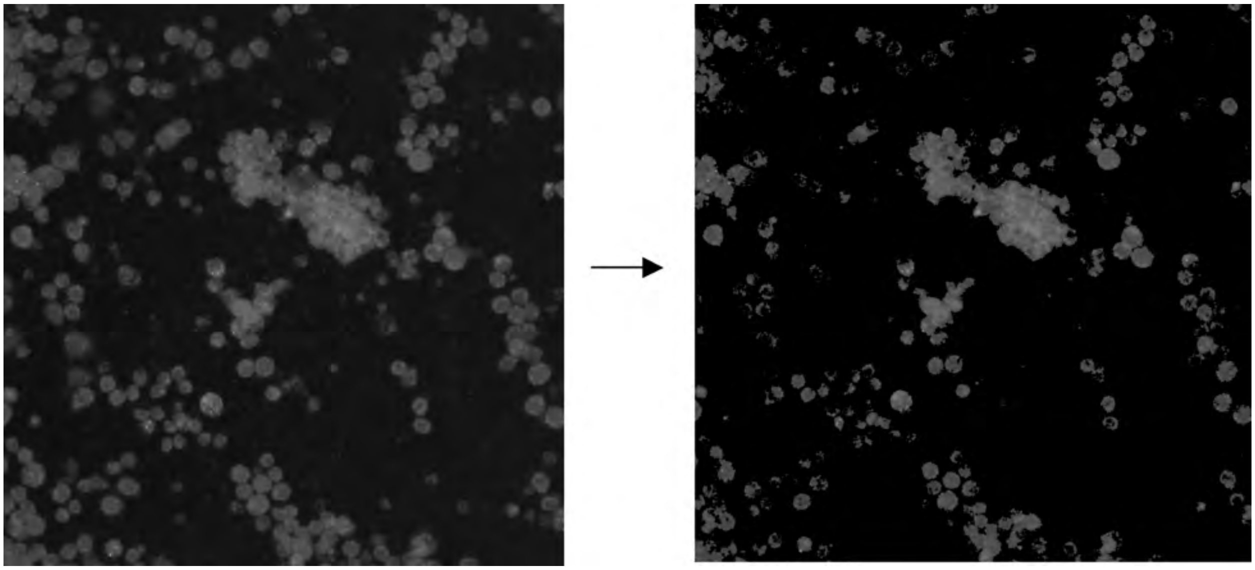

Module: Average

Help for the Average module:
Category: Image Processing

SHORT DESCRIPTION:
Averages images together (makes a projection).
****

This module averages a set of images by averaging the pixel intensities at each pixel position. When this module is used to average a Z-stack (3-D image stack), this process is known as making a projection.

Settings:

* What did you call the images to be averaged (made into a projection)?:
  Choose an image from among those loaded by a module or created by the pipeline, which will be averaged with the corresponding images of every image set.

* What do you want to call the averaged image?:
  Give a name to the resulting image, which could be used in subsequent modules. See the next setting for restrictions.

* Are the images you want to use to be loaded straight from a Load Images module, or are they being produced by the pipeline?:
  If you choose Load Images Module, the module will calculate the single, averaged image the first time through the pipeline (i.e. for cycle 1) by loading the image of the type specified above of every image set and averaging them together. It is then acceptable to use the resulting image later in the pipeline. Subsequent runs through the pipeline (i.e. for cycle 2 through the end) produce no new results. The averaged image calculated during the first cycle is still available to other modules during subsequent cycles.
  If you choose Pipeline, the module will calculate the single, averaged image during the last cycle of the pipeline. This is because it must wait for preceding modules in the pipeline to produce their results before it can calculate an averaged image. For example, you cannot calculate the average of all Cropped images until after the last image cycle completes and the last cropped image is produced. Note that in this mode, the resulting averaged image will not be available until the last cycle has been processed, so the averaged image it produces cannot be used in subsequent modules unless they are instructed to wait until the last cycle.

See also CorrectIllumination_Calculate.

Module: CalculateMath

```
Help for the Calculate Math module:
Category: Measurement

SHORT DESCRIPTION:
This module can take any measurements produced by previous modules and
can manipulate the numbers using basic arithmetic operations.
*************************************************************************

The arithmetic operations available in this module include addition,
subtraction, multiplication and division. The operation can be choosen
by adjusting the operations setting. The resulting data can also be
logged or raised to a power. This data can then be used in other
calculations and can be used in Classify Objects.

This module currently works on an object-by-object basis (it calculates
the requested operation for each object) but can also apply the operation
for measurements made for entire images (but only for measurements
produced by the Correlation module).

Feature Number:
The feature number specifies which features from the Measure module(s)
will be used for the operation. See each Measure module's help for the
numbered list of the features measured by that module.

See also CalculateRatios, all Measure modules.
```

Module: CalculateRatios

```
Help for the Calculate Ratios module:
Category: Measurement

SHORT DESCRIPTION:
Calculates the ratio between any measurements already measured (e.g.
Intensity of green staining in cytoplasm/Area of cells)
*************************************************************************

This module can take any measurements produced by previous modules and
calculate a ratio. Resulting ratios can also be used to calculate other
ratios and be used in Classify Objects.

This module can work on an object-by-object basis (calculating the ratio
for each object), on an image-by-image basis, or it can also calculate
ratios for object measurements by whole image measurements (to allow
normalization). Be careful with your denominator data. Any 0's found in
it will be changed to the average of the rest of the data. If all
denominator data is 0, all ratios will be set to 0 too.

The ratios will be stored under the numerator object's data. If the
numerator is an object, data will be under the name Ratio. If the
numerator is an image, data will be under the name SingleRatio or
MultipleRatio depending on whether the denominator is another image or an
object, respectively.

Feature Number:
The feature number specifies which features from the Measure module(s)
will be used for the ratio. See each Measure module's help for the
numbered list of the features measured by that module.

See also CalculateRatiosDataTool, all Measure modules.
```

Module: CalculateStatistics

Help for the Calculate Statistics module:
Category: Measurement

SHORT DESCRIPTION:
Calculates the V and Z' factors for measurements made from images.
****
Note: this module is beta-version and has not been thoroughly checked.

The V and Z' factors are statistical measures of assay quality and are calculated for each measurement that you have made in the pipeline. This allows you to choose which measures are most powerful for distinguishing positive and negative control samples. You must load a simple text file with one entry per cycle (using the Load Text module) that tells this module either which samples are positive and negative controls, or the concentrations of drugs.

The reference for Z' factor is: JH Zhang, TD Chung, et al. (1999) "A simple statistical parameter for use in evaluation and validation of high throughput screening assays." J Biomolecular Screening 4(2): 67-73.

The reference for V factor is: I Ravkin (2004): Poster #P12024 - Quality Measures for Imaging-based Cellular Assays. Society for Biomolecular Screening Annual Meeting Abstracts.

Module: ClassifyObjects

Help for the Classify Objects module:
Category: Object Processing

SHORT DESCRIPTION:
Classifies objects into different classes according to the value of a measurement you choose.
****

This module classifies objects into a number of different classes according to the value of a measurement (e.g. by size, intensity, shape). Choose the measurement feature to be used to classify your objects and specify what bins to use. This module requires that you run a measurement module previous to this module in the pipeline so that the measurement values can be used to classify the objects. If you are classifying by the ratio of two measurements, you must put a CalculateRatios module previous to this module in the pipeline.

Settings:

Feature Number:
The feature number specifies which feature from the Measure module will be used for classifying. See each Measure module's help for the numbered list of the features measured by that module.

If you are selecting Ratio, this is the order of ratio measurements that you calculated for the numerator. For instance, if you previously calculated the ratio of Area to Perimeter for nuclei, MajorAxisLength to MinorAxisLength for cells, and MeanIntensity to MaxIntensity for nuclei, the value for the Area to Perimeter for nuclei would be 1, the value for MajorAxisLength to MinorAxisLength for cells would be 2, and the value for MeanIntensity to MaxIntensity for nuclei would be 3.

See also ClassifyObjectsByTwoMeasurements, FilterByObjectMeasurement.

Module: ClassifyObjectsByTwoMeasurements

Help for the Classify Objects module:
Category: Object Processing

SHORT DESCRIPTION:
Classifies objects into different classes according to the value of two measurements of your choice.
****

This module classifies objects into four different classes according to the value of two measurements of your choice (e.g. size, intensity, shape). Choose the measurement features you want to use, and select a threshold for each set of data (measurements). The objects will then be separated in four classes: (1) objects whose first and second measurements are both below the specified thresholds, (2) objects whose first measurement is below the first threshold and whose second measurement is above the second threshold, (3) the opposite of class 2, and (4) objects whose first and second measurements are both above the specified thresholds. You can give names to the class/bins, or leave the default names of LowLow, LowHigh, HighLow, HighHigh. This module requires that you run measurement modules previous to this module in the pipeline so that the measurement values can be used to classify the objects. Currently, classifying by the ratio of two measurements is unavailable.

Settings:

Feature Number:
The feature number specifies which feature from the Measure module will be used for classifying. See each Measure module's help for the numbered list of the features measured by that module.

See also ClassifyObjects, FilterByObjectMeasurement.

Module: ColorToGray

```
Help for the Color To Gray module:
Category: Image Processing

SHORT DESCRIPTION:
Converts RGB (Red, Green, Blue) color images to grayscale. All channels
can be merged into one grayscale image (COMBINE option) or each channel
can be extracted into a separate grayscale image (SPLIT option).
*************************************************************************
Note: this module is especially helpful because all identify modules
require grayscale images.

Settings:

Split:
Takes a color image and splits the three channels (red, green, blue) into
three separate grayscale images.

Combine:
Takes a color image and converts it to grayscale by combining the three
channels (red, green, blue) together.

Adjustment factors: Leaving the adjustment factors set to 1 will balance
all three colors equally in the final image, which will use the same
range of intensities as the incoming image.  To weight colors relative to
each other, the adjustment factor can be increased (to increase the
weighting) or decreased (to decrease the weighting).

See also GrayToColor.
```

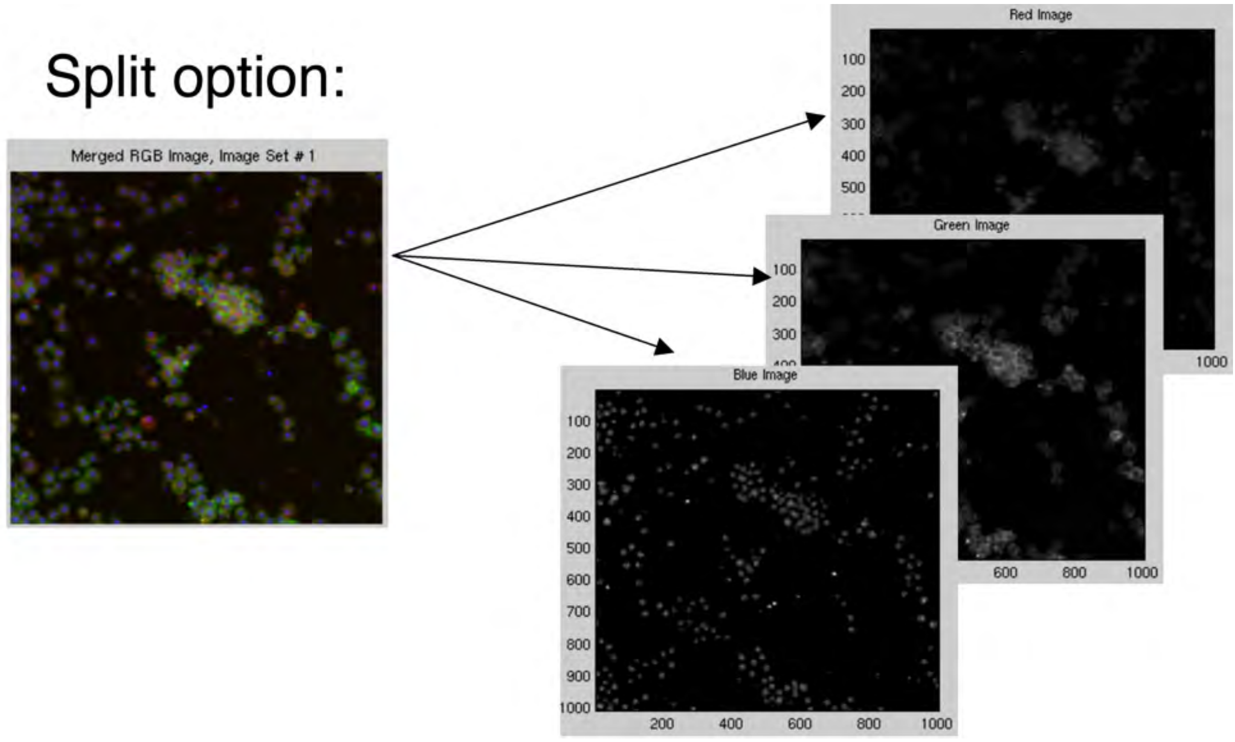

Module: Combine

```
Help for the Combine module:
Category: Image Processing

SHORT DESCRIPTION:
Takes 1 to 3 color or grayscale images and combines them into 1. Each image's
intensity can be adjusted independently.
*************************************************************************

This module takes color or grayscale images as inputs, and produces a new one
which results from the weighted average of the pixel intensities of the
input images. All the images to be combined must be all either grayscale
or color.  The average is found by taking the product of each weight
and the intensity of its corresponding image, adding the products, and
then dividing the result by the sum of the weights. By taking the
weighted average of the pixel intensities, the overall intensity of the
resulting image will remain the same as that of the inputs. If you want
to change the overall intensity, you should use the Rescale module.

Settings:

Choose the input images: You must select at least two images which you
would like to combine. They must all be the same size, since the average
will be taken pixel by pixel.

Weights: The weights will determine how each input image will affect the
combined one. The higher the weight of an image, the more it will be
reflected in the combined image. Because of the way the average is taken,
it only matters how these weights relate to each other (e.g. entering
weights 0.27, 0.3, and 0.3 is the same as entering weights 0.9, 1, and
1). Make sure all the weights have positive values.

See also GrayToColor, ColorToGray, and Rescale modules.
```

Module: ConvertToImage

```
Help for the Convert To Image module:
Category: Object Processing

SHORT DESCRIPTION:
Converts objects you have identified into an image so that it can be
saved with the Save Images module.
*************************************************************************

This module allows you to take previously identified objects and convert
them into an image, which can then be saved with the SaveImages modules.

Settings:

Binary (black & white), grayscale, or color: Choose how you would like
the objects to appear. Color allows you to choose a colormap which will
produce jumbled colors for your objects. Grayscale will give each object
a graylevel pixel intensity value corresponding to its number (also
called label), so it usually results in objects on the left side of the
image being very dark, and progressing towards white on the right side of
the image. You can choose "Color" with a "Gray" colormap to produce
jumbled gray objects.

Colormap:
Affect how the objects are colored. You can look up your default colormap
under File > Set Preferences. Look in matlab help online (try Google) to
see what the available colormaps look like. See also Help > HelpColormaps
in the main CellProfiler window.
```

Module: CorrectIlluminationApply

Help for the Correct Illumination Apply module:
Category: Image Processing

SHORT DESCRIPTION:
Applies an illumination function, created by CorrectIllumination_Calculate, to an image in order to correct for uneven illumination (uneven shading).
****

This module corrects for uneven illumination of each image. An illumination function image that represents the variation in illumination across the field of view is either made by a previous module or loaded by a previous module in the pipeline. This module then applies the illumination function to each image coming through the pipeline to produce the corrected image.

Settings:

Divide or Subtract:
This module either divides each image by the illumination function, or the illumination function is subtracted from each image. The choice depends on how the illumination function was calculated and on your physical model of how illumination variation affects the background of images relative to the objects in images. If the background is significant relative to the real signal coming from cells (a somewhat empirical decision), then the Subtract option may be preferable. If, in contrast, the signal to background ratio is quite high (the cells are stained strongly), then the Divide option is probably preferable. Typically, Subtract is used if the illumination function was calculated using the background option in the CORRECTILLUMINATION_CALCULATE module and divide is used if the illumination function was calculated using the regular option.

Rescaling:
If subtracting the illumination function, any pixels that end up negative are set to zero, so no rescaling of the corrected image is necessary. If dividing, the resulting corrected image may be in a very different range of intensity values relative to the original, depending on the values of the illumination function. If you are not rescaling, you should confirm that the illumination function is in a reasonable range (e.g. 1 to some number), so that the resulting image is in a reasonable range (0 to 1). Otherwise, you have two options to rescale the resulting image: either stretch the image so that the minimum is zero and the maximum is one, or match the maximum of the corrected image to the the maximum of the original. Either of these options has the potential to disturb the brightness of images relative to other images in the set, so caution should be used in interpreting intensity measurements from images that have been rescaled. See the help for the Rescale Intensity module for details.

See also CorrectIllumination_Calculate, RescaleIntensity.

Module: CorrectIlluminationCalculate

```
Help for the Correct Illumination Calculate module:
Category: Image Processing

SHORT DESCRIPTION:
Calculates an illumination function, used to correct uneven
illumination/lighting/shading or to reduce uneven background in images.
*************************************************************************

This module calculates an illumination function which can be saved to the
hard drive for later use (you should save in .mat format using the Save
Images module), or it can be immediately applied to images later in the
pipeline (using the CorrectIllumination_Apply module). This will correct
for uneven illumination of each image.

Illumination correction is challenging and we are writing a paper on it
which should help clarify it (TR Jones, AE Carpenter, P Golland, in
preparation). In the meantime, please be patient in trying to understand
this module.

Settings:

* Regular or Background intensities?

Regular intensities:
If you have objects that are evenly dispersed across your image(s) and
cover most of the image, then you can choose Regular intensities. Regular
intensities makes the illumination function based on the intensity at
each pixel of the image (or group of images if you are in All mode) and
is most often rescaled (see below) and applied by division using
CorrectIllumination_Apply. Note that if you are in Each mode or using a
small set of images with few objects, there will be regions in the
average image that contain no objects and smoothing by median filtering
is unlikely to work well.
Note: it does not make sense to choose (Regular + no smoothing + Each)
because the illumination function would be identical to the original
image and applying it will yield a blank image. You either need to smooth
each image or you need to use All images.

Background intensities:
If you think that the background (dim points) between objects show the
same pattern of illumination as your objects of interest, you can choose
Background intensities. Background intensities finds the minimum pixel
intensities in blocks across the image (or group of images if you are in
All mode) and is most often applied by subtraction using the
CorrectIllumination_Apply module.
Note: if you will be using the Subtract option in the
CorrectIllumination_Apply module, you almost certainly do NOT want to
Rescale! See below!!

* Each or All?
Enter Each to calculate an illumination function for each image
```

individually, or enter All to calculate the illumination function from all images at each pixel location. All is more robust, but depends on the assumption that the illumination patterns are consistent across all the images in the set and that the objects of interest are randomly positioned within each image. Applying illumination correction on each image individually may make intensity measures not directly comparable across different images.

* Pipeline or Load Images?
If you choose Load Images, the module will calculate the illumination correction function the first time through the pipeline by loading every image of the type specified in the Load Images module. It is then acceptable to use the resulting image later in the pipeline. If you choose Pipeline, the module will allow the pipeline to cycle through all of the cycles. With this option, the module does not need to follow a Load Images module; it is acceptable to make the single, averaged image from images resulting from other image processing steps in the pipeline. However, the resulting average image will not be available until the last cycle has been processed, so it cannot be used in subsequent modules unless they are instructed to wait until the last cycle.

* Dilation:
For some applications, the incoming images are binary and each object should be dilated with a gaussian filter in the final averaged (projection) image. This is for a sophisticated method of illumination correction where model objects are produced.

* Smoothing Method:
If requested, the resulting image is smoothed. See the help for the Smooth module for more details. If you are using Each mode, this is almost certainly necessary. If you have few objects in each image or a small image set, you may want to smooth. The goal is to smooth to the point where the illumination function resembles a believable pattern. That is, if it is a lamp illumination problem you are trying to correct, you would apply smoothing until you obtain a fairly smooth pattern without sharp bright or dim regions. Note that smoothing is a time-consuming process, and fitting a polynomial is fastest but does not allow a very tight fit as compared to the slower median filtering method.

* Approximate width of objects:
For certain smoothing methods, this will be used to calculate an adequate filter size. If you don't know the width of your objects, you can use the ShowOrHidePixelData image tool to find out or leave the word 'Automatic' to calculate a smoothing filter simply based on the size of the image.

Rescaling:
The illumination function can be rescaled so that the pixel intensities are all equal to or greater than one. This is recommended if you plan to use the division option in CorrectIllumination_Apply so that the corrected images are in the range 0 to 1. It is NOT recommended if you plan to use the Subtract option in CorrectIllumination_Apply! Note that as a result of the illumination function being rescaled from 1 to infinity, if there is substantial variation across the field of view, the

rescaling of each image might be dramatic, causing the corrected images to be very dark.

See also Average, CorrectIllumination_Apply, and Smooth modules.

Module: CorrectIlluminationNew

```
Help for the Correct Illumination New module:
Category: Image Processing

SHORT DESCRIPTION:
Beta
*************************************************************************

Beta

See also CorrectIllumination_Apply, Smooth
```

Module: CreateBatchFiles

```
Help for the Create Batch Files module:
Category: File Processing

SHORT DESCRIPTION:
Produces text files which allow individual batches of images to be
processed separately on a cluster of computers.
*************************************************************************

This module creates a set of files that can be submitted in parallel to a
cluster for faster processing. This module should be placed at the end of
an image processing pipeline.

Before using this module, you should read Help -> Getting Started ->
Batch Processing. That help file also will instruct you on how to
actually run the batch files that are created by this module.

Settings:
MATLAB or Compiled: If your cluster has MATLAB licenses for every node,
you can use either the full MATLAB source code (i.e. the Developer's
version) or the CPCluster MATLAB source code. If you do not have MATLAB
licenses for every node of your cluster, you can use the Compiled version
of CPCluster. These versions are available at www.cellprofiler.org. For
more information, please read Help -> Getting Started -> Batch
Processing.

Batch Size: This determines how many images will be analyzed in each set.
Under normal use, you do not want your batch size to be too large. If one
image in the batch fails for transient reasons, the entire batch will
have to start from the beginning. If you have a smaller batch size, the
job that failed will not take as long to re-run. Note: If you you do not
want to split a job into batches but want to send it to the cluster to be
processed all in one batch and free up your local computer for other
work, you can set the batch size to a very large number (more than the
total number of cycles) and this will create one large job which can be
submitted to the cluster.

Batch Prefix: This determines the prefix for all the batch files.

CellProfiler Path: Here you must specify the exact location of
CellProfiler files as seen by the cluster computers. If you want to use
the path that your local computer is currently using for CellProfiler,
leave a '.' in this box. Be sure that the cluster computers use the same
pathname and syntax.

Other Paths: You can either specify the exact paths as seen by the
cluster computers, or you can leave a period (.) to use the default image
and output folders. The last two settings allow you to use the default
image and output folders but switch the beginning path. For example, when
starting with a PC computer and going to a Linux machine, the path may be
the same except the first notation:
```

```
PC:    \\remoteserver1\cluster\project
Linux: /remoteserver2/cluster/project
```

In this case, for the local machine you would type "\\remoteserver1" and for the remote machine you would type "/remoteserver2". As of now, this is hardcoded to always end in Linux and Macintosh format using forward slashes (/).

Note:
* This module produces a Batch_data.mat file (the prefix Batch_ can be changed by the user). This contains the first image set's measurements plus information about the processing that each batch file needs access to in order to initialize the processing.
* In addition, this module produces one or more batch files of the form Batch_X_to_Y.m or Batch_X_to_Y.mat
- The prefix can be changed from Batch_ by the user.
- .m is used for the MATLAB version of CPCluster while .mat is used for the Compiled version of CPCluster.
- X is the first cycle to be processed in the particular batch file, and Y is the last.

See also MergeOutputFiles, GSBatchProcessing.

Module: CreateWebPage

```
Help for the Create Web Page module:
Category: Other

SHORT DESCRIPTION:
Creates the html for a webpage to display images (or their thumbnails, if
desired), including a link to a zipped file with all of the included
images.
*************************************************************************

This module will create an html file that will display the specified
images and also produce a zip-file of these images with a link. The
thumbnail images must be in the same directory as the original images.

Settings:
* Thumbnails: By default, the full-size images will be displayed on the
webpage itself. If you have made thumbnails (small versions of the
images), you can have these displayed on the webpage, and the full-size
images will be displayed when the user clicks on the thumbnails.

* Create webpage (HTML file) before or after processing all images?
If the full-size images and thumbnails (optional) already exist on the
hard drive and you are loading them with the Load Images module, you can
answer "Before" to this question. If, however, you are producing either
of these images during the pipeline and you therefore need to complete
all of the cycles before generating the webpage, choose "After".

* What do you want to call the resulting webpage file (include .htm or
.html as the extension)?
This file will be created in your default output directory. It can then
be copied to your web server. The primary difference between .htm and
.html is simply that .html can't be represented in a DOS/16 bit operating
system which uses the 8.3 file naming convention. Most servers (but not
all) that can handle 4 character file extensions can be set up to treat
.htm and .html files in exactly the same way, just as they can be set up
to treat .jpg and .jpeg files the same way.

* Will you have the webpage HTML file in the same folder or one level
above the images?
If the images are going to be in a subfolder, then the HTML file will be
one level above the images. If the HTML file and the images will all be
in the same folder, answer "Same as the images".

* Table border: If desired, there will be lines around each image,
creating a table. The thickness and color of these lines can be specified.

* Spacing between images: If this is set to greater than zero, there will
be an additional frame, the same color as the table border, around each
image. The spacing is the space between the frames that surrounds each
image.

* Image border width: This is the distance between each image and its
```

frame. If the spacing between images is zero, you will not see the frame itself, but the image border width will still affect the spacing between images.

Module: Crop

```
Help for the Crop module:
Category: Image Processing

SHORT DESCRIPTION:
Crops images into a rectangle, ellipse, an arbitrary shape provided by
the user, a shape identified by an identify module, or a shape used at a
previous step in the pipeline on another image.
*************************************************************************

Warnings about this module: The cropping module will remove rows and
columns that are completely blank. Also, keep in mind that cropping
changes the size of your images, which may have unexpected consequences.
For example, identifying objects in a cropped image and then trying to
measure their intensity in the *original* image will not work because the
two images are not the same size.

Settings:

Shape:
Rectangle - self-explanatory.
Ellipse - self-explanatory.
Other...
To crop based on an object identified in a previous module, type in the
name of that identified object instead of Rectangle or Ellipse. Please
see PlateFix for information on cropping based on previously identified
plates.
To crop into an arbitrary shape you define, use the LoadSingleImage
module to load a black and white image (that you have already prepared)
from a file. If you have created this image in an image program such as
Photoshop, this binary image should contain only the values 0 and 255,
with zeros (black) for the parts you want to remove and 255 (white) for
the parts you want to retain. Or, you may have previously generated a
binary image using this module (e.g. using the ellipse option) and saved
it using the SaveImages module (see Special note on saving images below).
In any case, the image must be the exact same starting size as your image
and should contain a contiguous block of white pixels, because keep in
mind that the cropping module will remove rows and columns that are
completely blank.
To crop into the same shape as was used previously in the pipeline to
crop another image, type in CroppingPreviousCroppedImageName, where
PreviousCroppedImageName is the image you produced with the previous Crop
module.

Coordinate or mouse: For ellipse, you will be asked to click five or more
points to define an ellipse around the part of the image you want to
analyze.  Keep in mind that the more points you click, the longer it will
take to calculate the ellipse shape. For rectangle, you can click as many
points as you like that are in the interior of the region you wish to
retain.

PlateFix: To be used only when cropping based on previously identified
```

objects. When attempting to crop based on a previously identified object (such as a yeast plate), sometimes the identified plate does not have precisely straight edges - there might be a tiny, almost unnoticeable 'appendage' sticking out of the plate. Without plate fix, the crop module would not crop the image tightly enough - it would include enough of the image to retain even the tiny appendage, so there would be a lot of blank space around the plate. This can cause problems with later modules (especially IlluminationCorrection). PlateFix takes the identified object and crops to exclude any minor appendages (technically, any horizontal or vertical line where the object covers less than 50% of the image). It also sets pixels around the edge of the object (for regions > 50% but less than 100%) that otherwise would be zero to the background pixel value of your image thus avoiding the problems with other modules. Important note >> PlateFix uses the coordinates entered in the boxes normally used for rectangle cropping (Top, Left) and (Bottom, Right) to tighten the edges around your identified plate. This is done because in the majority of plate identifications you do not want to include the sides of the plate. If you would like the entire plate to be shown, you should enter 1:end for both coordinates. If you would like to crop 80 pixels from each edge of the plate, you could enter 80:end-80 for (Top, Left) and (Bottom, Right).

Special note on saving images: See the help for the SaveImages module. Also, you can save the cropping shape that you have used (e.g. an ellipse you drew), so that in future analyses you can use the File option. To do this, you need to add the prefix "Cropping" to the name you called the cropped image (e.g. CroppingCropBlue) and this is the name of the image you will want to save using the SaveImages module. I think you will want to save it in mat format. You can also save the cropping shape, trimmed for any unused rows and columns at the edges. This image has the prefix "CropMask" plus the name you called the cropped image (e.g. CropMaskCropBlue). This image is used for downstream modules that use the CPgraythresh function. The Cropping and CropMask images are similar (both are binary and contain the cropping shape you used), but the Cropping image is the same size as the original images to be processed whereas the CropMask image is the same size as the final, cropped image.

Rectangular: enter the pixel coordinates for the left, top and right, bottom corners.

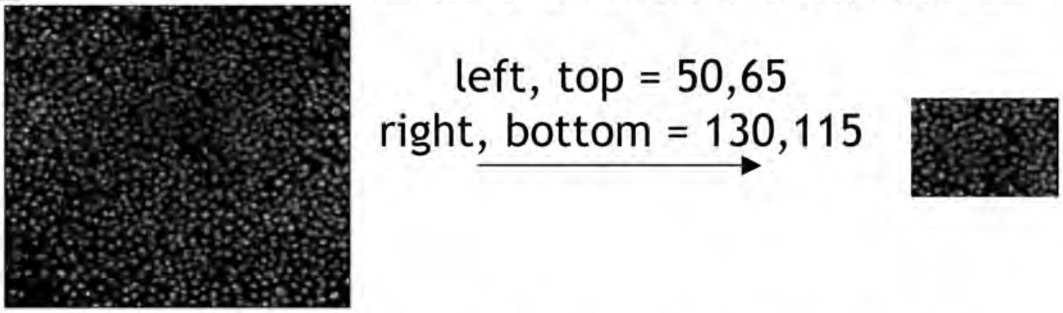

Any other shape: give CellProfiler™ a black and white image you have prepared which shows the cropping shape.

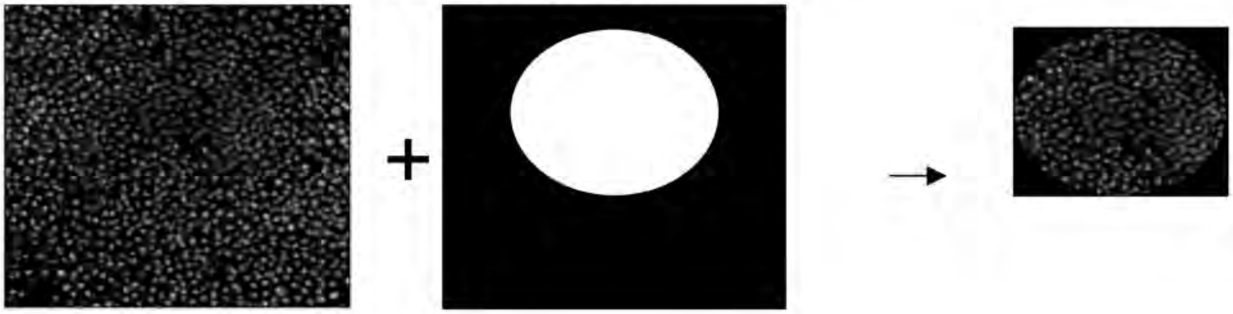

Module: DefineGrid

```
Help for the Define Grid module:
Category: Other

SHORT DESCRIPTION:
Produces a grid of desired specifications either manually or
automatically, based on previously identified objects. The grid can then
be used to make measurements (using Identify Objects in Grid) or to
display text information (using Display Grid Info) within each
compartment of the grid.
*************************************************************************

This module defines a grid that can be used by modules downstream.  If
you would like the grid to be defined automatically, an identify module
must be run prior to this module to identify the objects which will be
used to define the grid. The left-most, right-most, top-most, and
bottom-most object will be used to define the edges of the grid and the
rows and columns will be evenly spaced between these edges. Note that
automatic mode requires that the incoming objects are nicely defined -
for example, if there is an object at the edge of the images that is not
really an object that ought to be in the grid, a skewed grid will result.

Settings:
Most are self-explanatory.

You can choose to define a grid for EACH CYCLE, or ONCE for all
images. The grid can be defined using AUTOMATIC or MANUAL modes.

For MANUAL mode, several questions relate to the control spot. This
control spot can be specified by MOUSE or COORDINATES. For some
projects, you might have a control spot which is always present in every
image (e.g. the top left spot of the grid is always present). For
example, if the marker spot is within the grid itself at the top left
position, you could specify 0,0. As another example, if the control spot
is in the same row as the grid and is one spot to the left of the grid,
you could specify that the spot is -1,0 spot units away from the top left
spot.

See also IdentifyObjectsInGrid, DisplayGridInfo.
```

Module: DisplayDataOnImage

```
Help for the Display Data on Image module:
Category: Other

SHORT DESCRIPTION:
Produces an image with measured data on top of identified objects.
*************************************************************************

The resulting images with data on top can be saved using the Save Images
module.

Feature Number:
The feature number specifies which feature from the Measure module will
be used for display. See each Measure module's help for the numbered
list of the features measured by that module.

See also MeasureObjectAreaShape, MeasureImageAreaOccupied,
MeasureObjectIntensity, MeasureImageIntensity, MeasureTexture,
MeasureCorrelation, MeasureObjectNeighbors, and CalculateRatios modules.
```

Module: DisplayGridInfo

```
Help for the Display Grid Information module:
Category: Other

SHORT DESCRIPTION:
Displays text information on grid (i.e. gene names).
*************************************************************************

This module will display text information in a grid pattern. It requires
that you define a grid earlier in the pipeline using the DefineGrid
module and also load text information using the LoadText module. This
module allows you to load multiple sets of text data. The different sets
can be displayed in different colors. The text information must have the
same number of entries as there are grid locations (grid squares).

See also DefineGrid and LoadText.
```

Module: DisplayHistogram

```
Help for the Display Histogram module:
Category: Other

SHORT DESCRIPTION:
Produces a histogram of measurements.
*************************************************************************

The resulting histograms can be saved using the Save Images module.

Feature Number:
The feature number specifies which feature from the Measure module will
be used for the histogram. See each Measure module's help for the
numbered list of the features measured by that module.

Frequency counts:
Frequency counts refers to the threshold for the leftmost and rightmost
bins. The minimum value is the threshold at which any measurements less
than this value will be combined into the leftmost bin. The maximum value
is the threshold at which any measurements greater than or equal to this
value will be combined into the rightmosot bin.

Absolute vs. Percentage
Choose "Numbers" if you want the histogram bins to contain the actual
numbers of objects in the bin. Choose "Percents" if you want the
histogram bins to contain the percentage of objects in the bin.

See also DisplayImageHistogram, MeasureObjectAreaShape,
MeasureObjectIntensity, MeasureTexture, MeasureCorrelation,
MeasureObjectNeighbors, CalculateRatios.
```

Module: DisplayImageHistogram

```
Help for the Display Image Histogram module:
Category: Other

SHORT DESCRIPTION:
Produces a histogram of the intensity of pixels within an image.
*************************************************************************

This module creates a histogram that shows the pixel intensity of the
input image. The histogram can then be saved using the SaveImages module.

Settings:

How many histograms bins would you like to use?
Choose how many bins to use (i.e. in how many sets do you want the data
distributed).

Frequency counts:
Frequency counts refers to the threshold for the leftmost and rightmost
bins. The minimum value is the threshold at which any measurements less
than this value will be combined into the leftmost bin. The maximum value
is the threshold at which any measurements greater than or equal to this
value will be combined into the rightmosot bin.

See also DisplayHistogram, MeasureObjectAreaShape,
MeasureObjectIntensity, MeasureTexture, MeasureCorrelation,
MeasureObjectNeighbors, and CalculateRatios modules.
```

Module: DisplayMeasurement

```
Help for the Display Measurement module:
Category: Other

SHORT DESCRIPTION:
Plots measured data in several formats.
*************************************************************************

The DisplayMeasurement module allows data generated from the previous
modules to be displayed on a plot.  In the Settings, the type of the plot
can be specified.  The data can be displayed in a bar, line, or scatter
plot.  The user must choose the category of the data set to plot or, the
user may choose to plot a ratio of two data sets.  The scatterplot
requires additional information about the second set of measurements
used.
```

Module: Exclude

```
Help for the Exclude Objects module:
Category: Object Processing

SHORT DESCRIPTION:
Removes objects outside of specified region.
*************************************************************************

This image analysis module allows you to delete the objects and portions
of objects that are outside of a region you specify (e.g. nuclei outside
of a tissue region).  The objects and the region should both result from
any Identify module (Primary, Secondary, or Tertiary).

Retain or renumber:
Retaining objects' original numbers might be important if you intend to
correlate measurements made on the remaining objects with measurements
made on the original objects. Note that retaining original numbers will
produce gaps in the numbered list of objects (since some objects no
longer exist). This may cause errors with certain exporting tools or with
downstream modules that expect object numbers to not have gaps.
Renumbering, on the other hand, makes the output file more compact, the
processing quicker, and is also guaranteed to work with exporting and
data analysis tools.

Special note on saving images: Using the settings in this module, object
outlines can be passed along to the module Overlay Outlines and then
saved with the Save Images module. Objects themselves can be passed along
to the object processing module Convert To Image and then saved with the
Save Images module. This module produces several additional types of
objects with names that are automatically passed along with the following
naming structure: (1) The unedited segmented image, which includes
objects on the edge of the image and objects that are outside the size
range, can be saved using the name: UneditedSegmented + whatever you
called the objects (e.g. UneditedSegmentedNuclei). (2) The segmented
image which excludes objects smaller than your selected size range can be
saved using the name: SmallRemovedSegmented + whatever you called the
objects (e.g. SmallRemovedSegmented Nuclei).

See also FilterByObjectMeasurement, OverlayOutlines, ConvertToImage.
```

Module: ExpandOrShrink

```
Help for the Expand Or Shrink module:
Category: Object Processing

SHORT DESCRIPTION:
Expands or shrinks identified objects by a defined distance.
*************************************************************************

The module expands or shrinks objects by adding or removing border
pixels. The user can specify a certain number of border pixels to be
added or removed, or use 'Inf' to expand objects until they are almost
touching or to shrink objects down to a point. Objects are never lost
using this module (shrinking stops when an object becomes a single
pixel). An experimental feature is able to allow shrinking with secondary
objects - it adds partial dividing lines between objects which are
touching before the shrinking step so it is not perfect. It would be nice
to improve this code to draw complete dividing lines, but we have only
implemented a partial fix.

Special note on saving images: Using the settings in this module, object
outlines can be passed along to the module OverlayOutlines and then saved
with the SaveImages module. Objects themselves can be passed along to the
object processing module ConvertToImage and then saved with the
SaveImages module. This module produces several additional types of
objects with names that are automatically passed along with the following
naming structure: (1) The unedited segmented image, which includes
objects on the edge of the image and objects that are outside the size
range, can be saved using the name: UneditedSegmented + whatever you
called the objects (e.g. UneditedSegmentedNuclei). (2) The segmented
image which excludes objects smaller than your selected size range can be
saved using the name: SmallRemovedSegmented + whatever you called the
objects (e.g. SmallRemovedSegmented Nuclei).

See also IdentifyPrimAutomatic, IdentifyPrimManual, IdentifySecondary.
```

Shrink option:

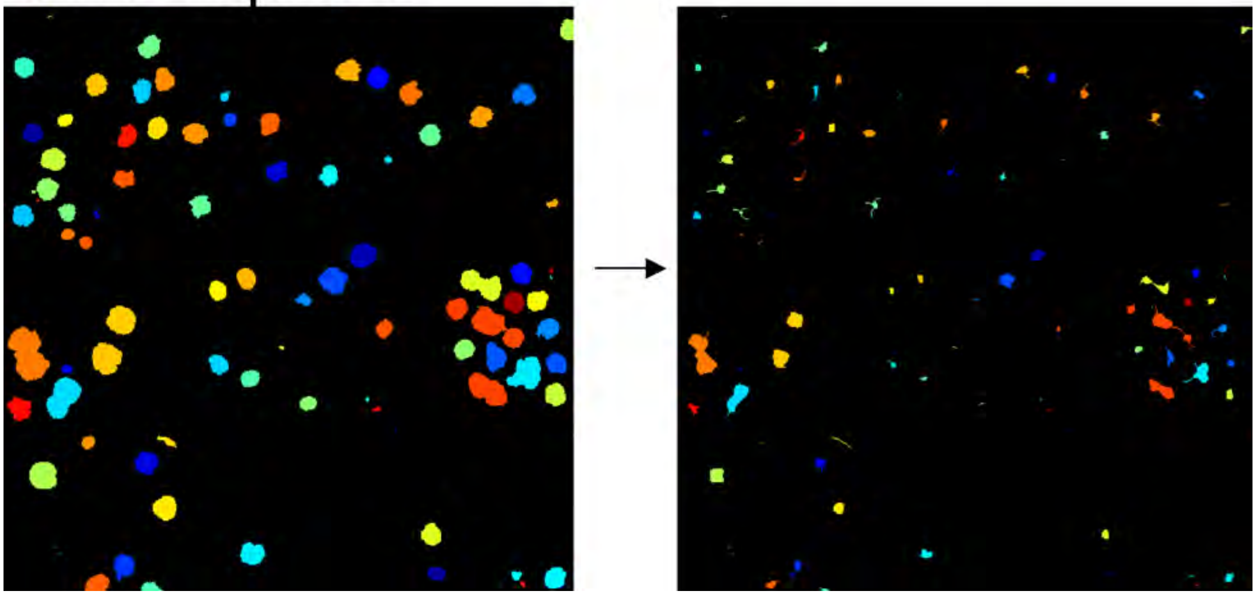

Module: ExportToDatabase

Help for the Export To Database module:
Category: File Processing

SHORT DESCRIPTION:
Exports data in database readable format, including an importing file with column names.
****

This module exports measurements to a SQL compatible format. It creates MySQL or Oracle scripts and associated data files which will create a database and import the data into it. This module must be run at the end of a pipeline, or second to last if you are using the CreateBatchFiles module. If you forget this module, you can also run the ExportDatabase data tool after processing is complete; its function is the same.

The database is set up with two primary tables. These tables are the Per_Image table and the Per_Object table (which may have a prefix if you specify). The Per_Image table consists of all the Image measurements and the Mean and Standard Deviation of the object measurements. There is one Per_Image row for every image. The Per_Object table contains all the measurements for individual objects. There is one row of object measurements per object identified. The two tables are connected with the primary key column ImageNumber. The Per_Object table has another primary key called ObjectNumber, which is unique per image.

The Oracle database has an extra table called Column_Names. This table is necessary because Oracle has the unfortunate limitation of not being able to handle column names longer than 32 characters. Since we must distinguish many different objects and measurements, our column names are very long. This required us to create a separate table which contains a short name and corresponding long name. The short name is simply "col" with an attached number, such as "col1" "col2" "col3" etc. The short name has a corresponding long name such as "Nuclei_AreaShape_Area". Each of the Per_Image and Per_Object columnnames are loaded as their "short name" but the long name can be determined from the Column_Names table.

Settings:

Database Type:
You can choose to export MySQL or Oracle database scripts. The exported data is the same for each type, but the setup files for MySQL and Oracle are different.

Database Name:
In MySQL, you can enter the name of a database to create or the name of an existing database. When using the script, if the database already exists, the database creation step will be skipped so the existing database will not be overwritten but new tables will be added. Do be careful, however, in choosing the Table Prefix. If you use an existing table name, you might unintentionally overwrite the data in that table.
In Oracle, when you log in you must choose a database to work with, so

there is no need to specify the database name in this module. This also means it is impossible to create/destroy a database with these CellProfiler scripts.

Table Prefix:
Here you can choose what to append to the table names Per_Image and Per_Object. If you choose "/", no prefix will be appended. If you choose a prefix, the tables will become PREFIX_Per_Image and PREFIX_Per_Object in the database. If you are using the same database for all of your experiments, the table prefix is necessary and will be the only way to distinguish different experiments. If you are creating a new database for every experiment, then it may be easier to keep the generic Per_Image and Per_Object table names. Be careful when choosing the table prefix, since you may unintentionally overwrite existing tables.

SQL File Prefix: All the CSV files will start with this prefix.

********************* How To Import MySQL *******************************
Step 1: Using a terminal, navigate to folder where the CSV output files and the SETUP script is located.

Step 2: Log into MySQL: "mysql -uUsername -pPassword -hHost"

Step 3: Run SETUP script: "\. DefaultDB_SETUP.SQL"

The SETUP file will do everything necessary to load the database.

********************* How To Import Oracle ******************************
Step 1: Using a terminal, navigate to folder where the CSV output files and the SETUP script is located.

Step 2: Log into SQLPlus: "sqlplus USERNAME/PASSWORD@DATABASESCRIPT"
You may need to ask your IT department the name of DATABASESCRIPT.

Step 3: Run SETUP script: "@DefaultDB_SETUP.SQL"

Step 4: Exit SQLPlus: "exit"

Step 5: Load data files (for columnnames, images, and objects):

```
sqlldr USERNAME/PASSWORD@DATABASESCRIPT control=DefaultDB_LOADCOLUMNS.ctl
sqlldr USERNAME/PASSWORD@DATABASESCRIPT control=DefaultDB_LOADIMAGE.ctl
sqlldr USERNAME/PASSWORD@DATABASESCRIPT control=DefaultDB_LOADOBJECT.ctl
```

Step 6: Log into SQLPlus: "sqlplus USERNAME/PASSWORD@DATABASESCRIPT"

Step 7: Run FINISH script: "@DefaultDB_FINISH.SQL"

Technical note: This module calls the CPconvertsql function to do the actual exporting, which is same function as called by the ExportDatabase data tool.

See also: CreateBatchFiles, ExportDatabase data tool.

Module: ExportToExcel

```
Help for the ExportToExcel module:
Category: File Processing

SHORT DESCRIPTION:
Exports measurements into a tab-delimited text file which can be opened
in Excel or other spreadsheet programs.
*************************************************************************
Note: this module is beta-version and has not been thoroughly checked.

The data will be converted to a tab-delimited text file which can be read
by Excel, another spreadsheet program, or a text editor. The file is
stored in the default output folder.

This module performs the same function as the data tool, Export Data.
Please refer to the help for ExportData.
```

Module: FilterByObjectMeasurement

Help for the Filter by Object Measurement module:
Category: Object Processing

SHORT DESCRIPTION:
Eliminates objects based on their measurements (e.g. area, shape, texture, intensity).
****

This module removes objects based on their measurements produced by another module (e.g. MeasureObjectAreaShape, MeasureObjectIntensity, MeasureTexture). All objects outside of the specified parameters will be discarded.

Feature Number:
The feature number specifies which feature from the Measure module will be used for filtering. See each Measure module's help for the numbered list of the features measured by that module.

Special note on saving images: Using the settings in this module, object outlines can be passed along to the module OverlayOutlines and then saved with the SaveImages module. Objects themselves can be passed along to the object processing module ConvertToImage and then saved with the SaveImages module. This module produces several additional types of objects with names that are automatically passed along with the following naming structure: (1) The unedited segmented image, which includes objects on the edge of the image and objects that are outside the size range, can be saved using the name: UneditedSegmented + whatever you called the objects (e.g. UneditedSegmentedNuclei). (2) The segmented image which excludes objects smaller than your selected size range can be saved using the name: SmallRemovedSegmented + whatever you called the objects (e.g. SmallRemovedSegmented Nuclei).

See also MeasureObjectAreaShape, MeasureObjectIntensity, MeasureTexture, MeasureCorrelation, CalculateRatios, and MeasureObjectNeighbors modules.

Module: FindEdges

```
Help for the Find Edges module:
Category: Image Processing

SHORT DESCRIPTION:
Identifies edges in an image, which can be used as the basis for object
identification or other downstream image processing.
*************************************************************************

This module finds the edges of objects in a grayscale image, usually
producing a binary (black and white) image where the edges are white and
the background is black. The ratio method can optionally produce a
grayscale image where the strongest edges are brighter and the smoothest
parts of the image are darker. It works best when the objects of interest
are black and the background is white.

Settings:

Threshold: Enter the desired threshold or have CellProfiler calculate one
automatically. The methods use different processes to calculate the
automatic threshold.

Threshold Adjustment Factor: This value will be multiplied by the
threshold (the numerical value you entered, or the automatically
calculated one if desired) used for edge detection.

Method: There are several methods that can be used to identify edges:
  Ratio Method - This method first applies two smoothing filters to the
                 image (sum of squares and square of sums), and then
                 takes the ratio of the two resulting images to determine
                 the edges. The filter size is then very important in
                 this method. The larger the filter size, the thicker the
                 edges will be. The recommended size is 8 pixels, or
                 roughly half the width of the objects you wish to edge.
                 This method is taken from CJ Cronin, JE Mendel, S
                 Mukhtar, Y-M Kim, RC Stirbl, J Bruck and PW Sternberg,
                 An automated system for measuring parameters of nematode
                 sinusoidal movement, BMC Genetics, 6:5, 2005 available
                 here: http://www.biomedcentral.com/1471-2156/6/5
  Sobel Method - finds edges using the Sobel approximation to the
                 derivative. It returns edges at those points where the
                 gradient of the image is maximum.
  Prewitt Method - finds edges using the Prewitt approximation to the
                 derivative. It returns edges at those points where the
                 gradient of the image is maximum.
  Roberts Method - finds edges using the Roberts approximation to the
                 derivative. It returns edges at those points where the
                 gradient of the image is maximum.
  LoG Method -   This method first applies a Laplacian of Gaussian filter
                 to the image and then finds zero crossings.
  Canny Method - The Canny method finds edges by looking for local maxima
                 of the gradient of the image. The gradient is calculated
```

using the derivative of a Gaussian filter. The method uses two thresholds, to detect strong and weak edges, and includes the weak edges in the output only if they are connected to strong edges. This method is therefore less likely than the others to be fooled by noise, and more likely to detect true weak edges.

Size of smoothing filter (for Ratio method only): A square of NxN will be used for the filter, where [illegible]
specify here. See method description above for further information.

Binary or Grayscale (for Ratio method only): The image produced by this module can be grayscale (varying shaed of gray) or binary (black and white). The choice depends on what you intend to use the resulting image for.

Edge Thinning (for Sobel and Roberts methods): If thinning is selected, edges found will be thinned out into a line (if possible). Specifying the 'nothinning' option can speed up the operation of the algorithm by skipping the additional edge thinning stage.

Direction (for Sobel and Prewitt methods): It gives you the option of identifying all edges, or just those that are predominantly horizontal or vertical.

Sigma (LoG and Canny): Standard deviation of the gaussian filter

Module: Flip

```
Help for the Flip module:
Category: Image Processing

SHORT DESCRIPTION:
Flips an image from top to bottom, left to right, or both.
*************************************************************************
```

Module: GrayToColor

```
Help for the Gray To Color module:
Category: Image Processing

SHORT DESCRIPTION:
Takes 1 to 3 images and assigns them to colors in a final red, green,
blue (RGB) image. Each color's brightness can be adjusted independently.
*************************************************************************

This module takes up to three grayscale images as inputs, and produces a
new color (RGB) image which results from assigning each of the input
images the colors red, green, and blue in the color image, respectively.
In addition, each color's intensity can be adjusted independently by
using adjustment factors (see below).

Settings:

Choose the input images: You must select at least one image which you
would like to use to create the color image. Also, all images must be the
same size, since they will combined pixel by pixel.

Adjustment factors: Leaving the adjustment factors set to 1 will balance
all three colors equally in the final image, and they will use the same
range of intensities as each individual incoming image. Using factors
less than 1 will decrease the intensity of that color in the final image,
and values greater than 1 will increase it. Setting the adjustment factor
to zero will cause that color to be entirely blank.

See also ColorToGray.
```

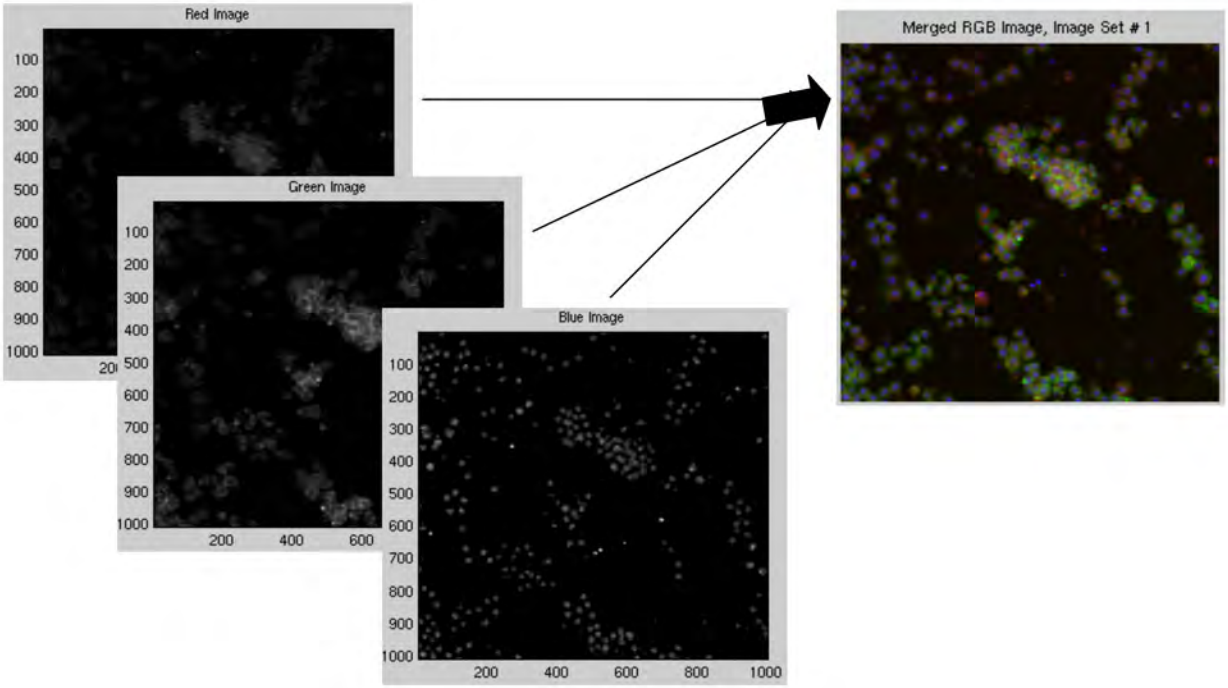

Module: IdentifyObjectsInGrid

Help for the Identify Objects In Grid module:
Category: Object Processing

SHORT DESCRIPTION:
Identifies objects within each section of a grid that has been defined by the DefineGrid module.
****

This module identifies objects that are in a grid pattern which allows you to measure the objects using measure modules. It requires that you create a grid in an earlier module using the DefineGrid module.

Settings:

For several of the automatic options, you will need to tell the module what you called previously identified objects. Typically, you roughly identify objects of interest in a previous Identify module, and the locations and/or shapes of these rough objects are refined in this module. Objects are also numbered according to the grid definitions. For the Natural Shape option, if an object does not exist within a grid compartment, an object consisting of one single pixel in the middle of the grid square will be created. Also, for the Natural Shape option, if a grid compartment contains two partial objects, they will be combined together as a single object.

If placing the objects within the grid is impossible for some reason (the grid compartments are too close together to fit the proper sized circles, for example) the grid will fail and processing will be canceled unless you choose to re-use the grid from the previous image cycle instead.

Special note on saving images: Using the settings in this module, object outlines can be passed along to the module OverlayOutlines and then saved with the SaveImages module. Objects themselves can be passed along to the object processing module ConvertToImage and then saved with the SaveImages module. This module produces several additional types of objects with names that are automatically passed along with the following naming structure: (1) The unedited segmented image, which includes objects on the edge of the image and objects that are outside the size range, can be saved using the name: UneditedSegmented + whatever you called the objects (e.g. UneditedSegmentedNuclei). (2) The segmented image which excludes objects smaller than your selected size range can be saved using the name: SmallRemovedSegmented + whatever you called the objects (e.g. SmallRemovedSegmented Nuclei).

See also DefineGrid.

Module: IdentifyPrimAutomatic

Help for the Identify Primary Automatic module:
Category: Object Processing

SHORT DESCRIPTION:
Identifies objects given only an image as input.
****

This module identifies primary objects (e.g. nuclei) in grayscale images that show bright objects on a dark background. The module has many options which vary in terms of speed and sophistication.

Requirements for the images to be fed into this module:
* If the objects are dark on a light background, they must first be inverted using the Invert Intensity module.
* If you are working with color images, they must first be converted to grayscale using the Color To Gray module.

Overview of the strategy ('Settings' below has more details):
  Properly identifying primary objects (nuclei) that are well-dispersed, non-confluent, and bright relative to the background is straightforward by applying a simple threshold to the image. This is fast but usually fails when nuclei are touching. In CellProfiler, several automatic thresholding methods are available, including global and adaptive, using Otsu's (Otsu, 1979) and our own version of a Mixture of Gaussians algorithm (O. Friman, unpublished). For most biological images, at least some nuclei are touching, so CellProfiler contains a novel modular three-step strategy based on previously published algorithms (Malpica et al., 1997; Meyer and Beucher, 1990; Ortiz de Solorzano et al., 1999; Wahlby, 2003; Wahlby et al., 2004). Choosing different options for each of these three steps allows CellProfiler to flexibly analyze a variety of different cell types. Here are the three steps:
  In step 1, CellProfiler determines whether an object is an individual nucleus or two or more clumped nuclei. This determination can be accomplished in two ways, depending on the cell type: When nuclei are bright in the middle and dimmer towards the edges (the most common case), identifying local maxima in the smoothed intensity image works well (Intensity option). When nuclei are quite round, identifying local maxima in the distance-transformed thresholded image (where each pixel gets a value equal to the distance to the nearest pixel below a certain threshold) works well (Shape option). For quick processing where cells are well-dispersed, you can choose to make no attempt to separate clumped objects.
  In step 2, the edges of nuclei are identified. For nuclei within the image that do not appear to touch, the edges are easily determined using thresholding. For nuclei that do appear to touch, there are two options for finding the edges of clumped nuclei. Where the dividing lines tend to be dimmer than the remainder of the nucleus (the most common case), the Intensity option works best (already identified nuclear markers are starting points for a watershed algorithm (Vincent and Soille, 1991) applied to the original image). When no dim dividing lines exist, the Distance option places the dividing line at a point between the two

nuclei determined by their shape (the distance-transformed thresholded image is used for the watershed algorithm). In other words, the dividing line is usually placed where indentations occur along the edge of the clumped nuclei.

In step 3, some identified nuclei are discarded or merged together if the user chooses. Incomplete nuclei touching the border of the image can be discarded. Objects smaller than a user-specified size range, which are likely to be fragments of real nuclei, can be discarded. Alternately, any of these small objects that touch a valid nucleus can be merged together based on a set of heuristic rules; for example similarity in intensity and statistics of the two objects. A separate module, FilterByObjectMeasurement, further refines the identified nuclei, if desired, by excluding objects that are a particular size, shape, intensity, or texture. This refining step could eventually be extended to include other quality-control filters, e.g. a second watershed on the distance transformed image to break up remaining clusters (Wahlby et al., 2004).

For more details, see the Settings section below and also the notation within the code itself (Developer's version).

Malpica, N., de Solorzano, C. O., Vaquero, J. J., Santos, A., Vallcorba, I., Garcia-Sagredo, J. M., and del Pozo, F. (1997). Applying watershed algorithms to the segmentation of clustered nuclei. Cytometry 28, 289-297.
Meyer, F., and Beucher, S. (1990). Morphological segmentation. J Visual Communication and Image Representation 1, 21-46.
Ortiz de Solorzano, C., Rodriguez, E. G., Jones, A., Pinkel, D., Gray, J. W., Sudar, D., and Lockett, S. J. (1999). Segmentation of confocal microscope images of cell nuclei in thick tissue sections. Journal of Microscopy-Oxford 193, 212-226.
Wahlby, C. (2003) Algorithms for applied digital image cytometry, Ph.D., Uppsala University, Uppsala.
Wahlby, C., Sintorn, I. M., Erlandsson, F., Borgefors, G., and Bengtsson, E. (2004). Combining intensity, edge and shape information for 2D and 3D segmentation of cell nuclei in tissue sections. J Microsc 215, 67-76.

Settings:

Typical diameter of objects, in pixel units (Min,Max):
This is a very important parameter which tells the module what you are looking for. Most options within this module use this estimate of the size range of the objects in order to distinguish them from noise in the image. For example, for some of the identification methods, the smoothing applied to the image is based on the minimum size of the objects. A comma should be placed between the minimum and the maximum diameters. The units here are pixels so that it is easy to zoom in on objects and determine typical diameters. To measure distances easily, use the CellProfiler Image Tool, 'ShowOrHidePixelData', in any open window. Once this tool is activated, you can draw a line across objects in your image and the length of the line will be shown in pixel units. Note that for non-round objects, the diameter here is actually the 'equivalent diameter', meaning the diameter of a circle with the same area as the object.

Discard objects outside the diameter range:
You can choose to discard objects outside the specified range of diameters. This allows you to exclude small objects (e.g. dust, noise, and debris) or large objects (e.g. clumps) if desired. See also the FilterByObjectMeasurement module to further discard objects based on some other measurement. During processing, the window for this module will show that objects outlined in green were acceptable, objects outlined in red were discarded based on their size, and objects outlined in yellow were discarded because they touch the border.

Try to merge 'too small' objects with nearby larger objects:
Use caution when choosing 'Yes' for this option! This is an experimental functionality that takes objects that were discarded because they were smaller than the specified Minimum diameter and tries to merge them with other surrounding objects. This is helpful in cases when an object was incorrectly split into two objects, one of which is actually just a tiny piece of the larger object. However, this could be dangerous if you have selected poor settings which produce many tiny objects - the module will take a very long time and you will not realize that it is because the tiny objects are being merged. It is therefore a good idea to run the module first without merging objects to make sure the settings are reasonably effective.

Discard objects touching the border of the image:
You can choose to discard objects that touch the border of the image. This is useful in cases when you do not want to make measurements of objects that are not fully within the field of view (because, for example, the area would not be accurate).

Select automatic thresholding method:

The threshold affects the stringency of the lines between the objects and the background. You can have the threshold automatically calculated using several methods, or you can enter an absolute number between 0 and 1 for the threshold (to see the pixel intensities for your images in the appropriate range of 0 to 1, use the CellProfiler Image Tool, 'ShowOrHidePixelData', in a window showing your image). There are advantages either way. An absolute number treats every image identically, but is not robust to slight changes in lighting/staining conditions between images. An automatically calculated threshold adapts to changes in lighting/staining conditions between images and is usually more robust/accurate, but it can occasionally produce a poor threshold for unusual/artifactual images. It also takes a small amount of time to calculate.

The threshold which is used for each image is recorded as a measurement in the output file, so if you find unusual measurements from one of your images, you might check whether the automatically calculated threshold was unusually high or low compared to the other images.

There are four methods for finding thresholds automatically, Otsu's method, the Mixture of Gaussian (MoG) method, the Background method, and the Ridler-Calvard method. The Otsu method uses our version of the Matlab function graythresh (the code is in the CellProfiler subfunction CPthreshold). Our modifications include taking into account the max and min values in the image and log-transforming the image prior to calculating the threshold. Otsu's method is probably better if you don't

know anything about the image, or if the percent of the image covered by objects varies substantially from image to image. But if you know the object coverage percentage and it does not vary much from image to image, the MoG can be better, especially if the coverage percentage is not near 50%. Note, however, that the MoG function is experimental and has not been thoroughly validated. The background function is very simple and is appropriate for images in which most of the image is background. It finds the mode of the histogram of the image, which is assumed to be the background of the image, and chooses a threshold at twice that value (which you can adjust with a Threshold Correction Factor, see below). This can be very helpful, for example, if your images vary in overall brightness but the objects of interest are always twice (or actually, any constant) as bright as the background of the image. The Ridler-Calvard method is simple and its results are often very similar to Otsu's. It chooses an initial threshold, and then iteratively calculates the next one by taking the mean of the average intensities of the background and foreground pixels determined by the first threshold, repeating this until the threshold converges.

You can also choose between global and adaptive thresholding, where global means that one threshold is used for the entire image and adaptive means that the threshold varies across the image. Adaptive is slower to calculate but provides more accurate edge determination which may help to separate clumps, especially if you are not using a clump-separation method (see below).

Threshold correction factor:
When the threshold is calculated automatically, it may consistently be too stringent or too lenient. You may need to enter an adjustment factor which you empirically determine is suitable for your images. The number 1 means no adjustment, 0 to 1 makes the threshold more lenient and greater than 1 (e.g. 1.3) makes the threshold more stringent. For example, the Otsu automatic thresholding inherently assumes that 50% of the image is covered by objects. If a larger percentage of the image is covered, the Otsu method will give a slightly biased threshold that may have to be corrected using a threshold correction factor.

Lower and upper bounds on threshold:
Can be used as a safety precaution when the threshold is calculated automatically. For example, if there are no objects in the field of view, the automatic threshold will be unreasonably low. In such cases, the lower bound you enter here will override the automatic threshold.

Approximate percentage of image covered by objects:
An estimate of how much of the image is covered with objects. This information is currently only used in the MoG (Mixture of Gaussian) thresholding but may be used for other thresholding methods in the future (see below).

Method to distinguish clumped objects:
Note: to choose between these methods, you can try test mode (see the last setting for this module).
* Intensity - For objects that tend to have only one peak of brightness per object (e.g. objects that are brighter towards their interiors), this option counts each intensity peak as a separate object. The objects can

be any shape, so they need not be round and uniform in size as would be required for a distance-based module. The module is more successful when the objects have a smooth texture. By default, the image is automatically blurred to attempt to achieve appropriate smoothness (see blur option), but overriding the default value can improve the outcome on lumpy-textured objects. Technical description: Object centers are defined as local intensity maxima.
* Shape - For cases when there are definite indentations separating objects. This works best for objects that are round. The intensity patterns in the original image are irrelevant - the image is converted to black and white (binary) and the shape is what determines whether clumped objects will be distinguished. Therefore, the cells need not be brighter towards the interior as is required for the Intensity option. The de-clumping results of this method are affected by the thresholding method you choose. Technical description: The binary thresholded image is distance-transformed and object centers are defined as peaks in this image.
* None (fastest option) - If objects are far apart and are very well separated, it may be unnecessary to attempt to separate clumped objects. Using the 'None' option, a simple threshold will be used to identify objects. This will override any declumping method chosen in the next question.

Method to draw dividing lines between clumped objects:
* Intensity - works best where the dividing lines between clumped objects are dim. Technical description: watershed on the intensity image.
* Distance - Dividing lines between clumped objects are based on the shape of the clump. For example, when a clump contains two objects, the dividing line will be placed where indentations occur between the two nuclei. The intensity patterns in the original image are irrelevant - the cells need not be dimmer along the lines between clumped objects. Technical description: watershed on the distance-transformed thresholded image.
* None (fastest option) - If objects are far apart and are very well separated, it may be unnecessary to attempt to separate clumped objects. Using the 'None' option, the thresholded image will be used to identify objects. This will override any declumping method chosen in the above question.

Size of smoothing filter, in pixel units:

(Only used when distinguishing between clumped objects) This setting, along with the suppress local maxima setting, affects whether objects close to each other are considered a single object or multiple objects. It does not affect the dividing lines between an object and the background. If you see too many objects merged that ought to be separate, the value should be lower. If you see too many objects split up that ought to be merged, the value should be higher.

The image is smoothed based on the specified minimum object diameter that you have entered, but you may want to override the automatically calculated value here. Reducing the texture of objects by increasing the smoothing increases the chance that each real, distinct object has only one peak of intensity but also increases the chance that two distinct objects will be recognized as only one object. Note that increasing the size of the smoothing filter increases the processing time exponentially.

Suppress local maxima within this distance (a positive integer, in pixel units):

(Only used when distinguishing between clumped objects) This setting, along with the size of the smoothing filter, affects whether objects close to each other are considered a single object or multiple objects. It does not affect the dividing lines between an object and the background. If you see too many objects merged that ought to be separate, the value should be lower. If you see too many objects split up that ought to be merged, the value should be higher.

Object markers are suppressed based on the specified minimum object diameter that you have entered, but you may want to override the automatically calculated value here. The maxima suppression distance should be set to be roughly equivalent to the minimum radius of a real object of interest. Basically, any distinct 'objects' which are found but are within two times this distance from each other will be assumed to be actually two lumpy parts of the same object, and they will be merged.

Speed up by using lower-resolution image to find local maxima?
(Only used when distinguishing between clumped objects) If you have entered a minimum object diameter of 10 or less, setting this option to Yes will have no effect.

Technical notes: The initial step of identifying local maxima is performed on the user-controlled heavily smoothed image, the foreground/background is done on a hard-coded slightly smoothed image, and the dividing lines between clumped objects (watershed) is done on the non-smoothed image.

Laplacian of Gaussian method:
This is a specialized method to find objects and will override the above settings in this module.

Special note on saving images: Using the settings in this module, object outlines can be passed along to the module OverlayOutlines and then saved with the SaveImages module. Objects themselves can be passed along to the object processing module ConvertToImage and then saved with the SaveImages module. This module produces several additional types of objects with names that are automatically passed along with the following naming structure: (1) The unedited segmented image, which includes objects on the edge of the image and objects that are outside the size range, can be saved using the name: UneditedSegmented + whatever you called the objects (e.g. UneditedSegmentedNuclei). (2) The segmented image which excludes objects smaller than your selected size range can be saved using the name: SmallRemovedSegmented + whatever you called the objects (e.g. SmallRemovedSegmented Nuclei).

See also IdentifyPrimManual, IdentifySecondary.

Module: IdentifyPrimManual

```
Help for the Identify Primary Manual module:
Category: Object Processing

SHORT DESCRIPTION:
Identifies an object based on manual intervention (clicking) by the user.
*************************************************************************

This module allows the user to identify objects by manually outlining
them. This is done by using the mouse to click multiple points around
the object. Multiple objects can be outlined using this module.

Special note on saving images: Using the settings in this module, object
outlines can be passed along to the module OverlayOutlines and then saved
with the SaveImages module. Objects themselves can be passed along to the
object processing module ConvertToImage and then saved with the
SaveImages module. This module produces several additional types of
objects with names that are automatically passed along with the following
naming structure: (1) The unedited segmented image, which includes
objects on the edge of the image and objects that are outside the size
range, can be saved using the name: UneditedSegmented + whatever you
called the objects (e.g. UneditedSegmentedNuclei). (2) The segmented
image which excludes objects smaller than your selected size range can be
saved using the name: SmallRemovedSegmented + whatever you called the
objects (e.g. SmallRemovedSegmented Nuclei).

See also IdentifyPrimAutomatic.
```

Module: IdentifySecondary

```
Help for the Identify Secondary module:
Category: Object Processing

SHORT DESCRIPTION:
Identifies objects (e.g. cell edges) using "seed" objects identified by
an Identify Primary module (e.g. nuclei).
*************************************************************************

This module identifies secondary objects (e.g. cell edges) based on two
inputs: (1) a previous module's identification of primary objects (e.g.
nuclei) and (2) an image stained for the secondary objects (not required
for the Distance - N option). Each primary object is assumed to be completely
within a secondary object (e.g. nuclei are completely within cells
stained for actin).

It accomplishes two tasks:
(a) finding the dividing lines between secondary objects which touch each
other. Three methods are available: Propagation, Watershed (an older
version of Propagation), and Distance.
(b) finding the dividing lines between the secondary objects and the
background of the image. This is done by thresholding the image stained
for secondary objects, except when using Distance - N.

Settings:

Methods to identify secondary objects:
* Propagation - For task (a), this method will find dividing lines
between clumped objects where the image stained for secondary objects
shows a change in staining (i.e. either a dimmer or a brighter line).
Smoother lines work better, but unlike the watershed method, small gaps
are tolerated. This method is considered an improvement on the
traditional watershed method. The dividing lines between objects are
determined by a combination of the distance to the nearest primary object
and intensity gradients. This algorithm uses local image similarity to
guide the location of boundaries between cells. Boundaries are
preferentially placed where the image's local appearance changes
perpendicularly to the boundary. Reference: TR Jones, AE Carpenter, P
Golland (2005) Voronoi-Based Segmentation of Cells on Image Manifolds,
ICCV Workshop on Computer Vision for Biomedical Image Applications, pp.
535-543. For task (b), thresholding is used.

* Watershed - For task (a), this method will find dividing lines between
objects by looking for dim lines between objects. For task (b),
thresholding is used. Reference: Vincent, Luc, and Pierre Soille,
"Watersheds in Digital Spaces: An Efficient Algorithm Based on Immersion
Simulations," IEEE Transactions of Pattern Analysis and Machine
Intelligence, Vol. 13, No. 6, June 1991, pp. 583-598.

* Distance - This method is bit unusual because the edges of the primary
objects are expanded a specified distance to create the secondary
objects. For example, if nuclei are labeled but there is no stain to help
```

locate cell edges, the nuclei can simply be expanded in order to estimate the cell's location. This is often called the 'doughnut' or 'annulus' or 'ring' approach for identifying the cytoplasmic compartment. Using the Distance - N method, the image of the secondary staining is not used at all, and these expanded objects are the final secondary objects. Using the Distance - B method, thresholding is used to eliminate background regions from the secondary objects. This allows the extent of the secondary objects to be limited to a certain distance away from the edge of the primary objects.

Select automatic thresholding method or enter an absolute threshold:
The threshold affects the stringency of the lines between the objects and the background. You can have the threshold automatically calculated using several methods, or you can enter an absolute number between 0 and 1 for the threshold (to see the pixel intensities for your images in the appropriate range of 0 to 1, use the CellProfiler Image Tool, 'ShowOrHidePixelData', in a window showing your image). There are advantages either way. An absolute number treats every image identically, but is not robust to slight changes in lighting/staining conditions between images. An automatically calculated threshold adapts to changes in lighting/staining conditions between images and is usually more robust/accurate, but it can occasionally produce a poor threshold for unusual/artifactual images. It also takes a small amount of time to calculate.

The threshold which is used for each image is recorded as a measurement in the output file, so if you find unusual measurements from one of your images, you might check whether the automatically calculated threshold was unusually high or low compared to the other images.

There are four methods for finding thresholds automatically, Otsu's method, the Mixture of Gaussian (MoG) method, the Background method, and the Ridler-Calvard method. The Otsu method uses our version of the Matlab function graythresh (the code is in the CellProfiler subfunction CPthreshold). Our modifications include taking into account the max and min values in the image and log-transforming the image prior to calculating the threshold. Otsu's method is probably better if you don't know anything about the image, or if the percent of the image covered by objects varies substantially from image to image. But if you know the object coverage percentage and it does not vary much from image to image, the MoG can be better, especially if the coverage percentage is not near 50%. Note, however, that the MoG function is experimental and has not been thoroughly validated. The background function is very simple and is appropriate for images in which most of the image is background. It finds the mode of the histogram of the image, which is assumed to be the background of the image, and chooses a threshold at twice that value (which you can adjust with a Threshold Correction Factor, see below). This can be very helpful, for example, if your images vary in overall brightness but the objects of interest are always twice (or actually, any constant) as bright as the background of the image. The Ridler-Calvard method is simple and its results are often very similar to Otsu's. It chooses and initial threshold, and then iteratively calculates the next one by taking the mean of the average intensities of the background and foreground pixels determined by the first threshold, repeating this until the threshold converges.

You can also choose between global and adaptive thresholding, where

global means that one threshold is used for the entire image and adaptive means that the threshold varies across the image. Adaptive is slower to calculate but provides more accurate edge determination.

Threshold correction factor:
When the threshold is calculated automatically, it may consistently be too stringent or too lenient. You may need to enter an adjustment factor which you empirically determine is suitable for your images. The number 1 means no adjustment, 0 to 1 makes the threshold more lenient and greater than 1 (e.g. 1.3) makes the threshold more stringent. For example, the Otsu automatic thresholding inherently assumes that 50% of the image is covered by objects. If a larger percentage of the image is covered, the Otsu method will give a slightly biased threshold that may have to be corrected using a threshold correction factor.

Lower and upper bounds on threshold:
Can be used as a safety precaution when the threshold is calculated automatically. For example, if there are no objects in the field of view, the automatic threshold will be unreasonably low. In such cases, the lower bound you enter here will override the automatic threshold.

Approximate percentage of image covered by objects:
An estimate of how much of the image is covered with objects. This information is currently only used in the MoG (Mixture of Gaussian) thresholding but may be used for other thresholding methods in the future (see below).

Regularization factor (for propagation method only):
This method takes two factors into account when deciding where to draw the dividing line between two touching secondary objects: the distance to the nearest primary object, and the intensity of the secondary object image. The regularization factor controls the balance between these two considerations: A value of zero means that the distance to the nearest primary object is ignored and the decision is made entirely on the intensity gradient between the two competing primary objects. Larger values weight the distance between the two values more and more heavily. The regularization factor can be infinitely large, but around 10 or so, the intensity image is almost completely ignored and the dividing line will simply be halfway between the two competing primary objects.

Note: Primary identify modules produce two (hidden) output images that are used by this module. The Segmented image contains the final, edited primary objects (i.e. objects at the border and those that are too small or large have been excluded). The SmallRemovedSegmented image is the same except that the objects at the border and the large objects have been included. These extra objects are used to perform the identification of secondary object outlines, since they are probably real objects (even if we don't want to measure them). Small objects are not used at this stage because they are more likely to be artifactual, and so they therefore should not "claim" any secondary object pixels.

TECHNICAL DESCRIPTION OF THE PROPAGATION OPTION:
Propagate labels from LABELS_IN to LABELS_OUT, steered by IMAGE and limited to MASK. MASK should be a logical array. LAMBDA is a

regularization parameter, larger being closer to Euclidean distance in the image plane, and zero being entirely controlled by IMAGE. Propagation of labels is by shortest path to a nonzero label in LABELS_IN. Distance is the sum of absolute differences in the image in a 3x3 neighborhood, combined with LAMBDA via sqrt(differences^2 + LAMBDA^2). Note that there is no separation between adjacent areas with different labels (as there would be using, e.g., watershed). Such boundaries must be added in a postprocess. IdentifySecPropagateSubfunction is the subfunction implemented in C and MEX to perform the propagate algorithm.

See also Identify primary modules.

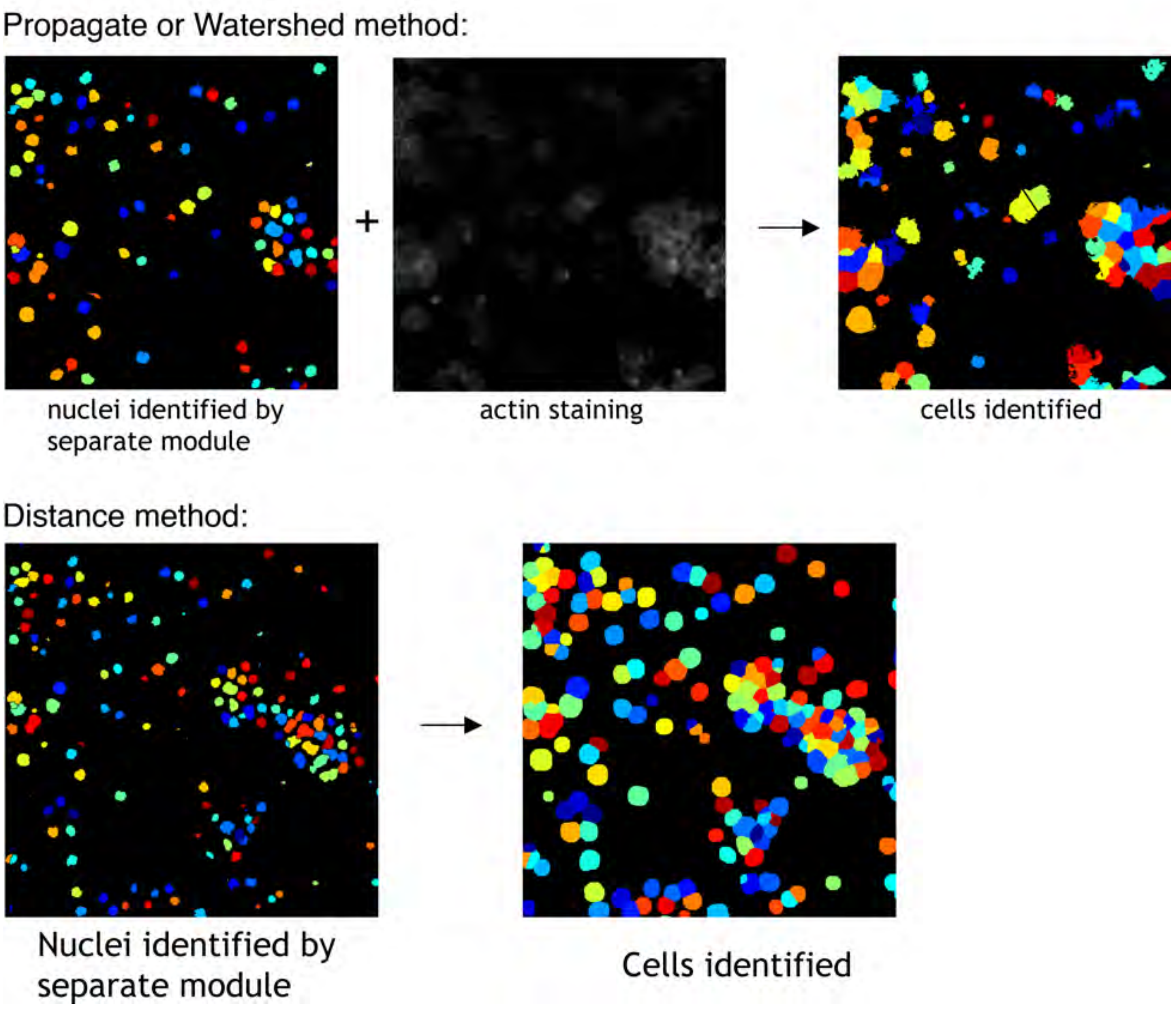

Module: IdentifyTertiarySubregion

```
Help for the Identify Tertiary Subregion module:
Category: Object Processing

SHORT DESCRIPTION:
Identifies tertiary obects (e.g. cytoplasm) by removing the primary
objects (e.g. nuclei) from secondary objects (e.g. cells) leaving a
ring shape.
*************************************************************************

This module will take the smaller identified objects and remove from them
the larger identified objects. For example, "subtracting" the nuclei from
the cells will leave just the cytoplasm, the properties of which can then
be measured by Measure modules. The larger objects should therefore be
equal in size or larger than the smaller objects and must completely
contain the smaller objects.  Both inputs should be objects produced by
identify modules, not images.

Note: creating subregions using this module can result in objects that
are not contiguous, which does not cause problems when running the
Measure Intensity and Texture modules, but does cause problems when
running the Measure Area Shape module because calculations of the
perimeter, aspect ratio, solidity, etc. cannot be made for noncontiguous
objects.

See also Identify Primary and Identify Secondary modules.
```

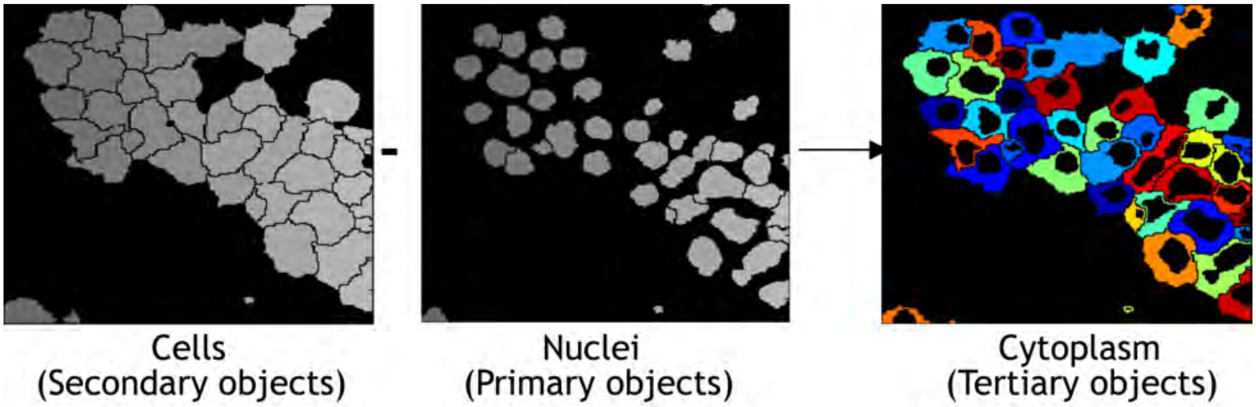

Module: InvertIntensity

Help for the Invert Intensity module:
Category: Image Processing

SHORT DESCRIPTION:
Converts the intensities of a grayscale image so that black becomes white.
****

The module does not work on color images. It takes a grayscale image and
inverts it (makes a negative) so that black is white and vice versa.

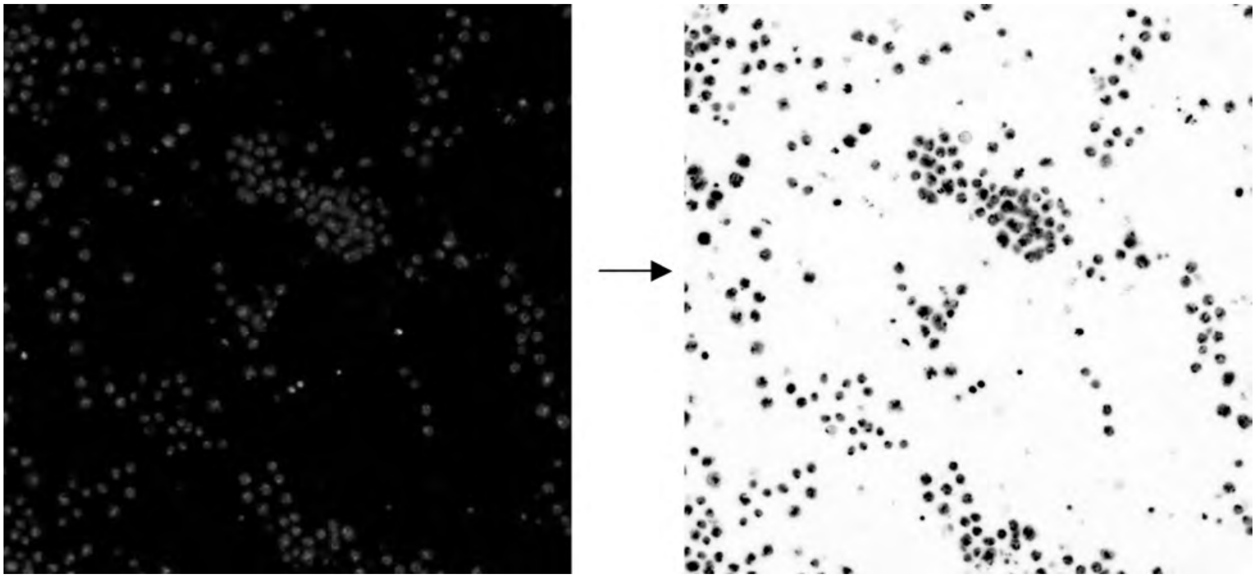

Module: LoadImages

Help for the Load Images module:
Category: File Processing

SHORT DESCRIPTION:
Allows you to specify which images or movies are to be loaded and in which order. Groups of images will be loaded per cycle of CellProfiler processing.
****

Tells CellProfiler where to retrieve images and gives each image a meaningful name for the other modules to access. When used in combination with a SaveImages module, you can load images in one file format and save in another file format, making CellProfiler work as a file format converter.

If more than four images per cycle must be loaded, more than one LoadImages module can be run sequentially. Running more than one of these modules also allows images to be retrieved from different folders. Hint: if you want to load all images in a directory, you can enter the file extension as the text for which to search.

Relative pathnames can be used. For example, in regular expressions text mode, on the Mac platform you could leave the folder where images are to be loaded as '.' to choose the default image folder, and then enter ../DAPI[123456789].tif as the name of the files you would like to load in order to load images from the directory one above the default image directory. Or, you could type .../AnotherSubfolder (note the three periods: the first is interpreted as a standin for the default image folder) as the folder from which images are to be loaded and enter the filename as .tif to load an image from a different subfolder of the parent of the default image folder.

Note: You can test a pipeline's settings on a single image cycle by setting the Load Images module appropriately. For example, if loading by order, you can set the number of images per set to equal the total number of images in the folder (even if it is thousands) so that only the first cycle will be analyzed. Or, if loading by text, you can make the identifying text specific enough that it will recognize only one group of images in the folder. Once the settings look good for a few test images, you can change the Load Images module to recognize all images in your folder.

Settings:

How do you want to load these files?
- Order is used when images (or movies) are present in a repeating order, like DAPI, FITC, Red, DAPI, FITC, Red, and so on, where images are selected based on how many images are in each group and what position within each group a particular color is located (e.g. three images per group, DAPI is always first).

- Text is used to load images (or movies) that have a particular piece of text in the name. You have the option of matching text exactly, or using regular expressions to match text. The files containing the text that are in image format will be loaded.

- When regular expressions is selected, patterns are specified using combinations of metacharacters and literal characters. There are a few classes of metacharacters, partially listed below. More extensive explanation can be found at:
  http://www.mathworks.com/access/helpdesk/help/techdoc/matlab_prog/
  f0-42649.html

The following metacharacters match exactly one character from its respective set of characters:

```
  Metacharacter   Meaning
 ---------------  --------------------------------
             .    Any character
            []    Any character contained within the brackets
           [^]    Any character not contained within the brackets
            \w    A word character [a-z_A-Z0-9]
            \W    Not a word character [^a-z_A-Z0-9]
            \d    A digit [0-9]
            \D    Not a digit [^0-9]
            \s    Whitespace [ \t\r\n\f\v]
            \S    Not whitespace [^ \t\r\n\f\v]
```

The following metacharacters are used to logically group subexpressions or to specify context for a position in the match. These metacharacters do not match any characters in the string:

```
  Metacharacter   Meaning
 ---------------  --------------------------------
           ()     Group subexpression
            |     Match subexpression before or after the |
            ^     Match expression at the start of string
            $     Match expression at the end of string
           \<     Match expression at the start of a word
           \>     Match expression at the end of a word
```

The following metacharacters specify the number of times the previous metacharacter or grouped subexpression may be matched:

```
  Metacharacter   Meaning
 ---------------  --------------------------------
            *     Match zero or more occurrences
            +     Match one or more occurrences
            ?     Match zero or one occurrence
        {n,m}     Match between n and m occurrences
```

Characters that are not special metacharacters are all treated literally in a match. To match a character that is a special metacharacter, escape that character with a '\'. For example '.' matches any character, so to match a '.' specifically, use '\.' in your pattern.

Examples:

* [trm]ail matches ’tail’ or ’rail’ or ’mail’
* [0-9] matches any digit between 0 to 9
* [^Q-S] matches any character other than ’Q’ or ’R’ or ’S’
* [[]A-Z] matches any upper case alphabet along with square brackets
* [ag-i-9] matches characters ’a’ or ’g’ or ’h’ or ’i’ or ’-’ or ’9’
* [a-p]* matches ’’ or ’a’ or ’aab’ or ’p’ etc.
* [a-p]+ matches ’a’ or ’abc’ or ’p’ etc.
* [^0-9] matches any string that is not a number
* ^[0-9]*$ matches any string that is a natural number or ’’
* ^-[0-9]+$|^\+?[0-9]+$ matches any integer

Analyze all subfolders within the selected folder?
You may have subfolders within the folder that is being searched, but if you are in TEXT mode, the names of the folders themselves must not contain the text you are searching for or an error will result.

Notes about loading images:

CellProfiler can open and read .ZVI files. .ZVI files are Zeiss files that are generated by the microscope imaging software Axiovision. These images are stored in 12-bit depth. Currently, CellProfiler cannot read stacked or color ZVI images.

CellProfiler can open and read .DIB files. These files are stored with 12-bit depth using a 16-bit file format.

Notes about loading movies:
Movies can be avi-formatted movies (must be uncompressed avi format on UNIX and Mac platforms) or stk-format movies (stacks of tif images produced by MetaMorph or NIHImage/ImageJ; The ability to read stk files is due to code by: Francois Nedelec, EMBL, Copyright 1999-2003). Once the files are identified, this module extracts each frame of each movie as a separate image, and gives these images a meaningful name for the other modules to access.

Suggestions for third party software to uncompress AVI files and convert MOV files:

WINDOWS...
To convert movies to uncompressed avi format, you can use a free software product called RAD Video Tools, which is available from:
  http://www.radgametools.com/down/Bink/RADTools.exe

To convert a compressed AVI file or a MOV file into an uncompressed AVI:
  1. Open RAD Video Tools
  2. Select the file you want to convert
  3. Click the "Convert a file" button
  4. On the next screen, type the desired output file name, and click the "Convert" button. Everything else can be left as default.
  5. A window will pop up that asks you for the Video Compression to use. Choose "Full Frames (Uncompressed)", and click OK.

```
MAC OSX...
The iMovie program which comes with Mac OSX can be used to convert movies
to uncompressed avi format as follows:

1. File > New Project
2. File > Import (select the movie)
3. File > Share
 Choose the QuickTime tab
 Compress movie for Expert Settings, click Share
 Name the file, choose Export: Movie to Avi
 Click Options...
 Click Settings...
 Compression = None
 Depth = Millions of Colors (NOT "+")
 Quality = best
 Frames per second = doesn't matter.
 OK, OK, Save

4. To check/troubleshoot the conversion, you can use the following
commands in Matlab:
>> MovieInfo = aviinfo('My Great Movie3.avi')

MovieInfo =
              Filename: 'My Great Movie3.avi'
              FileSize: 481292920
           FileModDate: '25-Mar-2005 09:59:56'
             NumFrames: 422
       FramesPerSecond: 20
                 Width: 720
                Height: 528
             ImageType: 'truecolor'
      VideoCompression: 'none'
               Quality: 4.2950e+07
    NumColormapEntries: 0

The following error means that the Depth was improper (either you tried
to save in grayscale or the wrong bit depth color):
>> movie = aviread('My Great Movie2.avi');
??? Error using ==> aviread
Bitmap data must be 8-bit Index images or 24-bit TrueColor images
------------------------------------------------------------------

See also LoadSingleImage.
```

Module: LoadSingleImage

```
Help for the Load Single Image module:
Category: File Processing

SHORT DESCRIPTION:
Loads a single image, which will be used for all image cycles.
*************************************************************************
Note: for most purposes, you will probably want to use the Load Images
module, not this one.

Tells CellProfiler where to retrieve a single image and gives the image a
meaningful name for the other modules to access.  The module only
functions the first time through the pipeline, and thereafter the image
is accessible to all subsequent cycles being processed. This is
particularly useful for loading an image like an Illumination correction
image to be used by the CorrectIllumination_Apply module. Note: Actually,
you can load four 'single' images using this module.

Relative pathnames can be used. For example, on the Mac platform you
could leave the folder where images are to be loaded as '.' to choose the
default image folder, and then enter ../Imagetobeloaded.tif as the name
of the file you would like to load in order to load the image from the
directory one above the default image directory. Or, you could type
.../AnotherSubfolder (note the three periods: the first is interpreted as
a standin for the default image folder) as the folder from which images
are to be loaded and enter the filename as Imagetobeloaded.tif to load an
image from a different subfolder of the parent of the default image
folder.

If more than four single images must be loaded, more than one Load Single
Image module can be run sequentially. Running more than one of these
modules also allows images to be retrieved from different folders.

LoadImages can now open and read .ZVI files.  .ZVI files are Zeiss files
that are generated by the microscope imaging software, Axiovision.  These
images are stored with 12-bit precision.  Currently, this will not work
with stacked or color images.

See also LoadImages.
```

Module: LoadText

```
Help for the Load Text module:
Category: File Processing

SHORT DESCRIPTION:
Loads text information corresponding to images. This data (e.g. gene
names or sample numbers) can be displayed on a grid or exported with the
measurements to help track samples.
*********************************************************************

Use this tool to load in text information. This is useful for two
reasons:
1. Some modules, like DisplayGridInfo place text information onto images.
In this case, the number of text entries that you load with this module
must be identical to the number of grid locations.
2. If the number of text entries that you load with this module is
identical to the number of cycles you are processing, the text
information you load will be placed in the output files alongside the
measurements that are made. Therefore, the information will be exported
with the measurements when you use the ExportData data tool, helping you
to keep track of your samples. If you forget this module, you can also
run the AddData data tool after processing is complete; its function is
the same for this purpose.

The text information to be loaded must be in a separate text file with
the following syntax:

DESCRIPTION <description>
<Text info 1>
<Text info 2>
<Text info 3>
             .
             .

<description> is a description of the text information stored in the
file. It can contain spaces or unusual characters.

For example:

DESCRIPTION Gene names
Gene X
Gene Y
Gene Z

Be sure that the file is saved in plain text format (.txt), not Rich Text
Format (.rtf).

While not thoroughly tested, most likely you can load numerical data too.

See also DisplayGridInfo module and AddData data tool.
```

Module: MaskImage

```
Help for the Mask Image module:
Category: Image Processing

SHORT DESCRIPTION:
Masks image and saves it for future use.
*************************************************************************

This module masks an image and saves it in the handles structure for
future use. The masked image is based on the original image and the
object selected. No display will be shown.

See also IdentifyPrimAutomatic, IdentifyPrimManual.
```

Module: MeasureCorrelation

```
Help for the Measure Correlation module:
Category: Measurement

SHORT DESCRIPTION:
Measures the correlation between intensities in different images (e.g.
different color channels) on a pixel by pixel basis, within identified
objects or across an entire image.
*************************************************************************

Given two or more images, calculates the correlation between the
pixel intensities. The correlation can be measured for the entire
images, or individual correlation measurements can be made within each
individual object. For example:
                                       Image overall:    In Nuclei:
OrigBlue  /OrigGreen      Correlation:    0.49955        -0.07395
OrigBlue  /OrigRed        Correlation:    0.59886        -0.02752
OrigGreen /OrigRed        Correlation:    0.83605         0.68489

See also MeasureObjectIntensity, MeasureImageIntensity.
```

Module: MeasureImageAreaOccupied

Help for the Measure Image Area Occupied module:
Category: Measurement

SHORT DESCRIPTION:
Measures total area covered by stain in an image.
****

This module simply measures the total area covered by stain in an image, using a threshold to determine stain vs background.

How it works:
This module applies a threshold to the incoming image so that any pixels brighter than the specified value are assigned the value 1 (white) and the remaining pixels are assigned the value zero (black), producing a binary image. The number of white pixels are then counted. This provides a measurement of the area occupied by the staining.

Settings:

* Select automatic thresholding method:
   The threshold affects the stringency of the lines between the objects and the background. You can have the threshold automatically calculated using several methods, or you can enter an absolute number between 0 and 1 for the threshold (to see the pixel intensities for your images in the appropriate range of 0 to 1, use the CellProfiler Image Tool, 'Show Or Hide Pixel Data', in a window showing your image). There are advantages either way. An absolute number treats every image identically, but is not robust to slight changes in lighting/staining conditions between images. An automatically calculated threshold adapts to changes in lighting/staining conditions between images and is usually more robust/accurate, but it can occasionally produce a poor threshold for unusual/artifactual images. It also takes a short time to calculate.
   The threshold which is used for each image is recorded as a measurement in the output file, so if you find unusual measurements from one of your images, you might check whether the automatically calculated threshold was unusually high or low compared to the other images.
   There are four methods for finding thresholds automatically, Otsu's method, the Mixture of Gaussian (MoG) method, the Background method, and the Ridler-Calvard method. The Otsu method uses our version of the Matlab function graythresh (the code is in the CellProfiler subfunction CPthreshold). Our modifications include taking into account the max and min values in the image and log-transforming the image prior to calculating the threshold. Otsu's method is probably better if you don't know anything about the image, or if the percent of the image covered by objects varies substantially from image to image. But if you know the object coverage percentage and it does not vary much from image to image, the MoG can be better, especially if the coverage percentage is not near 50%. Note, however, that the MoG function is experimental and has not been thoroughly validated. The background function is very simple and is

appropriate for images in which most of the image is background. It finds the mode of the histogram of the image, which is assumed to be the background of the image, and chooses a threshold at twice that value (which you can adjust with a Threshold Correction Factor, see below). This can be very helpful, for example, if your images vary in overall brightness but the objects of interest are always twice (or actually, any constant) as bright as the background of the image. The Ridler-Calvard method is simple and its results are often very similar to Otsu's. It chooses and initial threshold, and then iteratively calculates the next one by taking the mean of the average intensities of the background and foreground pixels determined by the first threshold, repeating this until the threshold converges.

* Threshold correction factor:
When the threshold is calculated automatically, it may consistently be too stringent or too lenient. You may need to enter an adjustment factor which you empirically determine is suitable for your images. The number 1 means no adjustment, 0 to 1 makes the threshold more lenient and greater than 1 (e.g. 1.3) makes the threshold more stringent. For example, the Otsu automatic thresholding inherently assumes that 50% of the image is covered by objects. If a larger percentage of the image is covered, the Otsu method will give a slightly biased threshold that may have to be corrected using a threshold correction factor.

* Lower and upper bounds on threshold:
Can be used as a safety precaution when the threshold is calculated automatically. For example, if there are no objects in the field of view, the automatic threshold will be unreasonably low. In such cases, the lower bound you enter here will override the automatic threshold.

* Approximate percentage of image covered by objects:
An estimate of how much of the image is covered with objects. This information is currently only used in the MoG (Mixture of Gaussian) thresholding but may be used for other thresholding methods in the future.

See also IdentifyPrimAutomatic, IdentifyPrimManual, and MeasureObjectAreaShape modules.

Module: MeasureImageIntensity

```
Help for the Measure Image Intensity module:
Category: Measurement

SHORT DESCRIPTION:
Measures the total image intensity by summing every pixel's intensity,
but can discard some pixel values if desired.
*************************************************************************

This module will sum all pixel values to measure the total image
intensity. The user can also choose to ignore pixels below or above a
particular intensity level.

Settings:

You may tell the module to ignore pixels above or below a pixel intensity
value that you specify, in the range 0 to 1 (use the CellProfiler image
tool 'ShowOrHidePixelData' to see the pixel intensities for your images
in the appropriate range of 0 to 1). Leaving these values at 0 and 1
means that every pixel intensity will be included in the measurement.
This setting is useful to adjust when you are attempting to exclude
bright artifactual objects: you can first set the threshold to exclude
these bright objects, but it may also be desirable to expand the
thresholded region around those bright objects by a certain distance so
as to avoid a 'halo' effect.

See also MeasureObjectIntensity.
```

Module: MeasureImageSaturationBlur

```
Help for the Measure Image Saturation and Blur module:
Category: Measurement

SHORT DESCRIPTION:
Measures the percentage of pixels in the image that are saturated and
measures blur (poor focus).
*************************************************************************

The percentage of pixels that are saturated is calculated and stored as a
measurement in the output file. 'Saturated' means that the pixel's
intensity value is equal to the maximum possible intensity value for that
image type.

The module can also measure blur by calculating a focus score (higher =
better focus). This calculation takes much longer than the saturation
checking, so it is optional. We are calculating the focus using the
normalized variance. We used this algorithm because it was ranked best in
this paper:
Sun, Y., Duthaler, S., Nelson, B. "Autofocusing in Computer Microscopy:
   Selecting the optimals focus algorithm." Microscopy Research and
   Technique 65:139-149 (2004)

The calculation of the focus score is as follows:
[m,n] = size(Image);
MeanImageValue = mean(Image(:));
SquaredNormalizedImage = (Image-MeanImageValue).^2;
FocusScore{ImageNumber} = ...
   sum(SquaredNormalizedImage(:))/(m*n*MeanImageValue);

Example Output:

Percent of pixels that are Saturated:
OrigBlue:   0.086173
OrigGreen:  0
OrigRed:    0

Focus Score:
OrigBlue:   0.47135
OrigGreen:  0.03440
OrigRed:    0.04652
```

Module: MeasureImageSlope

```
Help for the Measure Image Slope module:
Category: Measurement

SHORT DESCRIPTION:
This module measures the image slope as described by Ilya Ravkin.
*************************************************************************

Subsample Size:
Subsampling of the image for background removal given as fraction

Structuring Element Size:
Radius of structuring element (in subsampled image)

References for Granular Spectrum:
J.Serra, Image Analysis and Mathematical Morphology, Vol. 1. Academic
Press, London, 1989 Maragos,P. Pattern spectrum and multiscale shape
representation, IEEE Transactions on Pattern Analysis and Machine
Intelligence, 11, N 7, pp. 701-716, 1989

L.Vincent "Granulometries and Opening Trees", Fundamenta Informaticae,
41, No. 1-2, pp. 57-90, IOS Press, 2000.

L.Vincent "Morphological Area Opening and Closing for Grayscale Images",
Proc. NATO Shape in Picture Workshop, Driebergen, The Netherlands, pp.
197-208, 1992.

I.Ravkin, V.Temov Bit representation techniques and image processing,
Applied Informatics, v.14, pp. 41-90, Finances and Statistics, Moskow,
1988 (in Russian)
```

Module: MeasureObjectAreaShape

```
Help for the Measure Object Area Shape module:
Category: Measurement

SHORT DESCRIPTION:
Measures several area and shape features of identified objects.
*************************************************************************

Given an image with objects identified (e.g. nuclei or cells), this
module extracts area and shape features of each object. Note that these
features are only reliable for objects that are completely inside the
image borders, so you may wish to exclude objects touching the edge of
the image in Identify modules.

Basic shape features:     Feature Number:
Area                    |       1
Eccentricity            |       2
Solidity                |       3
Extent                  |       4
EulerNumber             |       5
Perimeter               |       6
FormFactor              |       7
MajorAxisLength         |       8
MinorAxisLength         |       9
Orientation             |      10

Zernike shape features:
    'Zernike_0_0'       |      11
    'Zernike_1_1'       |      12
    'Zernike_2_0'       |      13
    'Zernike_2_2'       |      14
    'Zernike_3_1'       |      15
    'Zernike_3_3'       |      16
    'Zernike_4_0'       |      17
    'Zernike_4_2'       |      18
    'Zernike_4_4'       |      19
    'Zernike_5_1'       |      20
    'Zernike_5_3'       |      21
    'Zernike_5_5'       |      22
    'Zernike_6_0'       |      23
    'Zernike_6_2'       |      24
    'Zernike_6_4'       |      25
    'Zernike_6_6'       |      26
    'Zernike_7_1'       |      27
    'Zernike_7_3'       |      28
    'Zernike_7_5'       |      29
    'Zernike_7_7'       |      30
    'Zernike_8_0'       |      31
    'Zernike_8_2'       |      32
    'Zernike_8_4'       |      33
    'Zernike_8_6'       |      34
    'Zernike_8_8'       |      35
```

```
    'Zernike_9_1'       |       36
    'Zernike_9_3'       |       37
    'Zernike_9_5'       |       38
    'Zernike_9_7'       |       39
    'Zernike_9_9'       |       40
```

Zernike shape features measure shape by describing a binary object (or more precisely, a patch with background and an object in the center) in a basis of Zernike polynomials, using the coefficients as features (Boland et al., 1998). Currently, Zernike polynomials from order 0 to order 9 are calculated, giving in total 30 measurements. While there is no limit to the order which can be calculated (and indeed users could add more by adjusting the code), the higher order polynomials carry less information.

Details about how measurements are calculated:
This module retrieves objects in label matrix format and measures them. The label matrix image should be "compacted": that is, each number should correspond to an object, with no numbers skipped. So, if some objects were discarded from the label matrix image, the image should be converted to binary and re-made into a label matrix image before feeding into this module.

The following measurements are extracted using the Matlab regionprops.m function:
*Area - Computed from the the actual number of pixels in the region.
*Eccentricity - Also known as elongation or elongatedness. For an ellipse that has the same second-moments as the object, the eccentricity is the ratio of the between-foci distance and the major axis length. The value is between 0 (a circle) and 1 (a line segment).
*Solidity - Also known as convexity. The proportion of the pixels in the convex hull that are also in the object. Computed as Area/ConvexArea.
*Extent - The proportion of the pixels in the bounding box that are also in the region. Computed as the Area divided by the area of the bounding box.
*EulerNumber - Equal to the number of objects in the image minus the number of holes in those objects. For modules built to date, the number of objects in the image is always 1.
*MajorAxisLength - The length (in pixels) of the major axis of the ellipse that has the same normalized second central moments as the region.
*MinorAxisLength - The length (in pixels) of the minor axis of the ellipse that has the same normalized second central moments as the region.
*Perimeter - the total number of pixels around the boundary of each region in the image.

In addition, the following feature is calculated:

FormFactor = 4*pi*Area/Perimeter^2, equals 1 for a perfectly circular object

See also MeasureImageAreaOccupied.

Module: MeasureObjectIntensity

```
Help for the Measure Object Intensity module:
Category: Measurement

SHORT DESCRIPTION:
Measures several intensity features for identified objects.
*************************************************************************

Given an image with objects identified (e.g. nuclei or cells), this
module extracts intensity features for each object based on a
corresponding grayscale image. Measurements are recorded for each object.

Measurement:             Feature Number:
IntegratedIntensity     |       1
MeanIntensity           |       2
StdIntensity            |       3
MinIntensity            |       4
MaxIntensity            |       5
IntegratedIntensityEdge |       6
MeanIntensityEdge       |       7
StdIntensityEdge        |       8
MinIntensityEdge        |       9
MaxIntensityEdge        |      10
MassDisplacement        |      11

How it works:
Retrieves objects in label matrix format and a corresponding original
grayscale image and makes measurements of the objects. The label matrix
image should be "compacted": that is, each number should correspond to an
object, with no numbers skipped. So, if some objects were discarded from
the label matrix image, the image should be converted to binary and
re-made into a label matrix image before feeding it to this module.

Intensity Measurement descriptions:

* IntegratedIntensity - The sum of the pixel intensities within an
object.
* MeanIntensity - The average pixel intensity within an object.
* StdIntensity - The standard deviation of the pixel intensities within
an object.
* MaxIntensity - The maximal pixel intensity within an object.
* MinIntensity - The minimal pixel intensity within an object.
* IntegratedIntensityEdge - The sum of the edge pixel intensities of an
object.
* MeanIntensityEdge - The average edge pixel intensity of an object.
* StdIntensityEdge - The standard deviation of the edge pixel intensities
of an object.
* MaxIntensityEdge - The maximal edge pixel intensity of an object.
* MinIntensityEdge - The minimal edge pixel intensity of an object.
* MassDisplacement - The distance between the centers of gravity in the
gray-level representation of the object and the binary representation of
the object.
```

See also MeasureImageIntensity.

Module: MeasureObjectNeighbors

Help for the Measure Object Neighbors module:
Category: Measurement

SHORT DESCRIPTION:
Calculates how many neighbors each object has.
****

Given an image with objects identified (e.g. nuclei or cells), this module determines how many neighbors each object has. The user selects the distance within which objects should be considered neighbors. The module can measure the number of neighbors each object has if every object were expanded up until the point where it hits another object. To use this option, enter 0 (the number zero) for the pixel distance.

How it works:
Retrieves objects in label matrix format. The objects are expanded by the number of pixels the user specifies, and then the module counts up how many other objects the object is overlapping.

Interpreting the module output:
In the color image output of the module, there is a color spectrum used to determine which objects have neighbors, and how many. According to the indices on the spectrum, the background is -1, objects with no neighbors are 0, and objects with neighbors are greater than 0, with the increasing index corresponding to more neighbors.

Saving the objects:
* You can save the objects colored by number of neighbors to the handles structure to be used in other modules. Here, the scalar value 1 is added to every pixel so that the background is zero and the objects range from 1 up to the highest number of neighbors, plus one. This makes the objects compatible with the Convert To Image module.

Saving the image:
* You can save the grayscale image of objects to the handles structure so it can be saved to the hard drive. Here, the background is -1, and the objects range from 0 (if it has no neighbors) up to the highest number of neighbors. The -1 value makes it incompatible with the Convert To Image module which expects a label matrix starting at zero.

Module: MeasureTexture

```
Help for the Measure Texture module:
Category: Measurement

SHORT DESCRIPTION:
Measures several texture features for identified objects or for entire
images.
*************************************************************************

Given an image with objects identified (e.g. nuclei or cells), this
module extracts texture features for each object based on a corresponding
grayscale image. Measurements are recorded for each object. If "Image" is
chosen, the texture of the image overall is measured.

How it works:
Retrieves objects in label matrix format and a corresponding original
grayscale image and makes measurements of the objects. The label matrix
image should be "compacted": that is, each number should correspond to an
object, with no numbers skipped. So, if some objects were discarded from
the label matrix image, the image should be converted to binary and
re-made into a label matrix image before feeding into this module.

The scale of texture measured is chosen by the user, in pixel units. A
higher number for the scale of texture measures larger patterns of
texture whereas smaller numbers measure more localized patterns of
texture. It is best to measure texture on a scale smaller than your
objects sizes, so be sure that the value entered for scale of texture is
smaller than most of your objects. For very small objects (smaller than
the scale of texture you are measuring), the texture cannot be measured
and will result in a value of NaN (Not a Number) in the output file.

Note that texture measurements are affected by the overall intensity of
the object (or image). For example, if Image1 = Image2 + 0.2, then the
texture measurements should be the same for Image1 and Image2. However,
if the images are scaled differently, for example Image1 = 0.9*Image2,
then this will be reflected in the texture measurements, and they will be
different. For example, in the extreme case of Image1 = 0*Image2 it is
obvious that the texture measurements must be different. To make the
measurements useful (both intensity, texture, etc.), it must be ensured
that the images are scaled similarly. In other words, if differences in
intensity are seen between two images or objects, the differences in
texture cannot be trusted as being completely independent of the
intensity difference.

Measurement:             Feature Number:
AngularSecondMoment     |       1
Contrast                |       2
Correlation             |       3
Variance                |       4
InverseDifferenceMoment |       5
SumAverage              |       6
SumVariance             |       7
```

```
SumEntropy            |      8
Entropy               |      9
DifferenceVariance    |     10
DifferenceEntropy     |     11
InformationMeasure    |     12
InformationMeasure2   |     13
GaborX                |     14
GaborY                |     15
```

Texture Measurement descriptions:

Haralick Features:
Haralick texture features are derived from the co-occurrence matrix, which contains information about how image intensities in pixels with a certain position in relation to each other occur together. For example, how often does a pixel with intensity 0.12 have a neighbor 2 pixels to the right with intensity 0.15? The current implementation in CellProfiler uses a shift of 1 pixel to the right for calculating the co-occurence matrix. A different set of measurements is obtained for larger shifts, measuring texture on a larger scale. The original reference for the Haralick features is Haralick et al. (1973) Textural Features for Image Classification. IEEE Transaction on Systems Man, Cybernetics, SMC-3(6):610-621, where 14 features are described:
H1. Angular Second Moment
H2. Contrast
H3. Correlation
H4. Sum of Squares: Variation
H5. Inverse Difference Moment
H6. Sum Average
H7. Sum Variance
H8. Sum Entropy
H9. Entropy
H10. Difference Variance
H11. Difference Entropy
H12. Information Measure of Correlation 1
H13. Information Measure of Correlation 2
H14. Max correlation coefficient

*H14 is disabled because it is computationally demanding.

Gabor "wavelet" features:
These features are similar to wavelet features, and they are obtained by applying so-called Gabor filters to the image. The Gabor filters measure the frequency content in different orientations. They are very similar to wavelets, and in the current context they work exactly as wavelets, but they are not wavelets by a strict mathematical definition. As currently implemented, the frequency content of the object is measured along the x- and y-axis (i.e. in two different orientations). The original reference is Gabor, D. (1946). "Theory of communication" Journal of the Institute of Electrical Engineers, 93:429-441.

Module: Morph

Help for the Morph module:
Category: Image Processing

SHORT DESCRIPTION:
Beta version: provides access to built in Matlab morphological functions.
****

Beta version: provides access to built in Matlab morphological functions.

Settings:

Beta

Module: OverlayOutlines

```
Help for the Overlay Outlines module:
Category: Image Processing

SHORT DESCRIPTION:
Places outlines produced by an identify module over a desired image.
*************************************************************************

Outlines (in a special format produced by an identify module) can be
placed on any desired image (grayscale or color) and then this resulting
image can be saved using the SaveImages module.

Settings:
Would you like to set the intensity (brightness) of the outlines to be
the same as the brightest point in the image, or the maximum possible
value for this image format?
If your image is quite dim, then putting bright white lines onto it may
not be useful. It may be preferable to make the outlines equal to the
maximal brightness already occurring in the image.

See also identify modules.
```

Module: PlaceAdjacent

```
Help for the Place Adjacent module:
Category: Image Processing

SHORT DESCRIPTION:
Places up to six images next to each other, either horizontally or
vertically, to produce a single image.
*************************************************************************

To place together many images, you can use this module multiple times in
one pipeline.

See also Tile.
```

Module: Relate

```
Help for the Relate module:
Category: Object Processing

SHORT DESCRIPTION:
Assigns relationships: All objects (e.g. speckles) within a parent object
(e.g. nucleus) become its children.
*************************************************************************

Allows counting the number of objects within each parent object. An
object will be considered a child even if the edge is the only part
touching a parent object. If an object is touching two parent objects,
the objects parent will be the higher numbered parent.
```

Module: RenameOrRenumberFiles

```
Help for the Rename Or Renumber Files module:
Category: File Processing

SHORT DESCRIPTION:
Renames or renumbers files on the hard drive.
*************************************************************************

File renaming utility that deletes or adds text anywhere within image
file names. It is especially useful as a file renumbering utility that
converts numbers within image file names to solve improper ordering of
files on Unix/Mac OSX systems.

The settings ask the user to indicate how many characters to retain at
the beginning and end of each filename. These are the characters that
will remain unchanged. The user may choose to add text or numbers between
the characters that are to be retained.

Be very careful since you will be renaming (= overwriting) your files!!
You will have the opportunity to confirm the name change for the first
cycle only. The folder containing the files must not contain subfolders
or the subfolders and their contents will also be renamed. It is worth
doing a practice run with copies of images first.

Examples:

Renumber:
DrosDAPI_1.tif    -> DrosDAPI_001.tif
DrosDAPI_10.tif   -> DrosDAPI_010.tif
DrosDAPI_100.tif  -> DrosDAPI_100.tif
(to accomplish this, retain 4 characters at the end, retain 9 characters
at the beginning, and use 3 numerical digits between).

Rename:
1DrosophilaDAPI_1.tif    -> 1DrosDP_1.tif
2DrosophilaDAPI_10.tif   -> 2DrosDP_10.tif
3DrosophilaDAPI_100.tif  -> 3DrosDP_100.tif
(to accomplish this, retain 4 characters at the end, retain 5 characters
at the beginning, enter "DP" as text to place between, and leave
numerical digits as is).
```

Module: RescaleIntensity

```
Help for the Rescale Intensity module:
Category: Image Processing

SHORT DESCRIPTION:
Changes intensity range of an image to desired specifications.
*************************************************************************

The intensity of the incoming images are rescaled by one of several
methods. This is especially helpful for converting 12-bit images saved in
16-bit format to the correct range (see method E).

Settings:

Rescaling method:
(S) Stretch the image so that the minimum is zero and the maximum is
one.
(E) Enter the minimum and maximum values of the original image and the
desired resulting image. Pixels are scaled from their user-specified
original range to a new user-specified range.  If the user enters "AE"
(Automatic for Each), then the highest and lowest pixel values will be
Automatically computed for each image by taking the maximum and minimum
pixel values in each image.  If the user enters "AA" (Automatic for All),
then the highest and lowest pixel values will be Automatically computed
by taking the maximum and minimum pixel values in all the images in the
set. Pixels in the original image that are above or below the original
range are pinned to the high/low values of that range before being
scaled. To convert 12-bit images saved in 16-bit format to the correct
range, use the settings 0, 0.0625, 0, 1.  The value 0.0625 is equivalent
to 2^12 divided by 2^16, so it will convert a 16 bit image containing
only 12 bits of data to the proper range.
(G) rescale the image so that all pixels are equal to or Greater
than one.
(M) Match the maximum of one image to the maximum of another.
(C) Convert to 8 bit: Images in CellProfiler are normally stored as
numerical class double in the range of 0 to 1. This option converts these
images to class uint8, meaning an 8 bit integer in the range of 0 to 255.
This is useful to reduce the amount of memory required to store the
image. Warning: Most CellProfiler modules require the incoming image to
be in the standard 0 to 1 range, so this conversion may cause downstream
modules to behave unexpectedly.

See also SubtractBackground.
```

Module: Resize

```
Help for the Resize module:
Category: Image Processing

SHORT DESCRIPTION:
Resizes images.
*************************************************************************

Images are resized (smaller or larger) based on the user's inputs. You
can resize an image by applying a resizing factor or by specifying a
pixel size for the resized image. You can also select which interpolation
method to use. This module uses the MATLAB built-in function imresize.
```

Module: Restart

```
Help for the Restart module:
Category: File Processing

SHORT DESCRIPTION:
Restarts image analysis which had failed or was canceled, using the
partially completed output file.
*************************************************************************

Restarts an analysis run where it left off. Put Restart into a new
pipeline with no other modules. Click Analyze images. When the dialog
"Choose a settings or output file" appears, select the output file of the
incomplete run. Click OK and the pipeline will load from the output file
and analysis will continue where it left off during the partially
completed run.
```

Module: Rotate

Help for the Rotate module:
Category: Image Processing

SHORT DESCRIPTION:
Rotates images either automatically or by user's mouse input.
****

Settings:

Rotation method:
*Coordinates - you can provide the X,Y pixel locations of two points in the image which should be aligned horizontally or vertically.
*Mouse - you can click on points in the image which should be aligned horizontally or vertically.
*Angle - you can provide the numerical angle by which the image should be rotated.

Would you like to crop away the rotated edges?
When an image is rotated, there will be black space at the corners/edges unless you choose to crop away the incomplete rows and columns of the image. This cropping will produce an image that is not the exact same size as the original, which may affect downstream modules.

See also Flip, Crop.

Module: SaveImages

Help for the Save Images module:
Category: File Processing

SHORT DESCRIPTION:
Saves any image produced during the image analysis, in any image format.
****

Because CellProfiler usually performs many image analysis steps on many groups of images, it does *not* save any of the resulting images to the hard drive unless you use the SaveImages module to do so. Any of the processed images created by CellProfiler during the analysis can be saved using this module.

You can choose from among 18 image formats to save your files in. This allows you to use the module as a file format converter, by loading files in their original format and then saving them in an alternate format.

Please note that this module works for the cases we have tried, but it has not been extensively tested, particularly for how it handles color images, non-8 bit images, images coming from subdirectories, multiple incoming movie files, or filenames made by numerical increments.

Settings:

Update file names within CellProfiler:
This allows downstream modules (e.g. CreateWebPage) to look up the newly saved files on the hard drive. Normally, whatever files are present on the hard drive when CellProfiler processing begins (and when the LoadImages module processes its first cycle) are the only files that are accessible within CellProfiler. This setting allows the newly saved files to be accessible to downstream modules. This setting might yield unusual consequences if you are using the SaveImages module to save an image directly as loaded (e.g. using the SaveImages module to convert file formats), because it will, in some places in the output file, overwrite the file names of the loaded files with the file names of the the saved files. Because this function is rarely needed and may introduce complications, the default answer is "No".

Special notes for saving in movie format (avi):
The movie will be saved after the last cycle is processed. You have the option to also save the movie periodically during image processing, so that the partial movie will be available in case image processing is canceled partway through. Saving movies in avi format is quite slow, so you can enter a number to save the movie after every Nth cycle. For example, entering a 1 will save the movie after every cycle. When working with very large movies, you may also want to save the CellProfiler output file every Nth cycle to save time, because the entire movie is stored in the output file (this may only be the case if you are working in diagnostic mode, see Set Preferences). See the SpeedUpCellProfiler module. If you are processing multiple movies, especially movies in subdirectories, you should save after every cycle (and also, be aware

that this module has not been thoroughly tested under those conditions). Note also that the movie data is stored in the handles.Pipeline.Movie structure of the output file, so you can retrieve the movie data there in case image processing is aborted. At the time this module was written, MATLAB was only capable of saving in uncompressed avi format (at least on the UNIX platform), which is time and space-consuming. You should convert the results to a compressed movie format, like .mov using third-party software. For suggested third-party software, see the help for the LoadImages module.

See also LoadImages, SpeedUpCellProfiler.

Module: SendEmail

```
Help for the Send Email module:
Category: Other

SHORT DESCRIPTION:
Sends emails to a specified address at desired stages of the processing.
*************************************************************************

This module emails the user-specified recipients about the current
progress of the image processing as well as the expected time remaining
until completion. The user can specify how often emails are sent out
(for example, after the first cycle, after the last cycle, after every X
cycles, after N cycles).
Note: This module should be placed at the point in the pipeline when you
want the emails to be sent.
```

Module: Smooth

```
Help for the Smooth module:
Category: Image Processing

SHORT DESCRIPTION:
Smooths (blurs) images.
*************************************************************************

Sorry, this module's documentation is out of date. It will be documented
soon.

Settings:

Smoothing Method:
Note that smoothing is a time-consuming process, and fitting a polynomial
is fastest but does not allow a very tight fit as compared to the slower
median filtering method. Width of artifacts over ~50 take substantial
amounts of time to process.

Special note on saving images: If you want to save the smoothed image to
use it for later analysis, you should save the smoothed image in '.mat'
format to prevent degradation of the data.

Technical note on the median filtering method: the artifact width is
divided by two to obtain the radius of a disk-shaped structuring element
which is used for filtering. No longer done this way.

See also CorrectIllumination_Apply, CorrectIllumination_Calculate.
```

Module: SpeedUpCellProfiler

Help for the Speed Up CellProfiler module:
Category: Other

SHORT DESCRIPTION:
Speeds up CellProfiler processing and conserves memory.
****

Speeds up CellProfiler processing and conserves memory by reducing the frequency of saving partial output files and/or clearing the memory.

Settings:

* Output files should be saved every Nth cycle?
To save the output file after every cycle, as usual, leave this set to 1. Entering a larger integer allows faster image processing by refraining from saving the output file after every cycle is processed. Instead, the output file is saved after every Nth cycle (and always after the first and last cycles). For large output files, this can result in substantial time savings. The only disadvantage is that if processing is canceled prematurely, the output file will contain only data up to the last cycle that was a multiple of N, even if several cycles have been processed since then. Another hint: be sure you are not in Diagnostic mode (see File > Set Preferences) to avoid saving very large output files with intermediate images, because this slows down CellProfiler as well.

* Do you want to clear the memory?
If yes, everything in temporary memory will be removed except for the images you specify. Therefore, only the images you specify will be accessible to modules downstream in the pipeline. This module can therefore be used to clear space in the memory.
Note: currently, this option will remove everything in the memory, which may not be compatible with some modules, which often store non-image information in memory to be re-used during every cycle.

* Do you want to pack the memory?
If yes, CellProfiler will perform memory garbage collection. All needed variables will be saved on the disk, the memory will be completely cleared, and then the saved variables will be reloaded for use.

Module: SplitOrSpliceMovie

```
Help for the Split Or Splice Movie module:
Category: File Processing

SHORT DESCRIPTION:
Creates one large movie from several small movies, or creates several
small movies from one large movie.
*************************************************************************

This module is only compatible with AVI format movies.

Settings:

Where are the existing avi-formatted movies?
Typing a period (.) will use the default image folder. Relative folder
locations will work also (e.g.   ../SIBLINGFOLDER)

Where do you want to put the resulting files?
Typing a period (.) will use the default output folder. Relative folder
locations will work also (e.g.   ../SIBLINGFOLDER)

For SPLICE, what is the common text in your movie file names?
The files to be spliced should all be located within a single folder. You
can choose a subset of movies in the folder to splice by specifying
common text in their names. To splice all movies in the folder, you can
just enter the file extension (e.g. '.avi').

For SPLIT, you can split only one movie at a time, and the full file name
should be entered here.

For SPLIT, how many frames per movie do you want?
The way CellProfiler reads movie files is that it reads each movie frame
by frame. It will open the first frame and run through the pipeline then
open the next and do the same. This is done until there are no more
frames. Indicating the number of frames can be seen as also indicating
the number cycles that a pipeline will be run.

Note: This module is run by itself in a pipeline; there is no need to use
a LoadImages or SaveImages module.
```

Module: Subtract

```
Help for the Subtract module:
Category: Image Processing

SHORT DESCRIPTION:
Subtracts the intensities of one image from another.
*************************************************************************

Settings:
Subtracting may substantially change the range of pixel intensities in
the resulting image, so each image can be multiplied by a factor prior to
subtracting. This factor can be a positive number.

Do you want negative values in the image to be set to zero?
Values outside the range of 0 to 1 might not be handled well by other
modules. Here, you have the option of setting negative values to 0.
For other options (e.g. setting values over 1 to equal 1), see the
Rescale Intensity module.

See also SubtractBackground, RescaleIntensity.
```

Module: SubtractBackground

```
Help for the Subtract Background module:
Category: Image Processing

SHORT DESCRIPTION:
Calculates the minimum pixel intensity value for the entire set of images
and subtracts this value from every pixel in every image.
*************************************************************************

Note that this is not an illumination correction module. It subtracts a
single value from every pixel across the image.

The intensity due to camera or illumination or antibody background
(intensity where no cells are sitting) can in good conscience be
subtracted from the images, but it must be subtracted from every pixel,
not just the pixels where cells actually are sitting.  This is because we
assume that this staining is additive with real staining. This module
calculates the lowest possible pixel intensity across the entire image
set and subtracts this background value from every pixel in every image.
This module is identical to the Apply Threshold module (in shift mode),
except in the SubtractBackground module, the threshold is automatically
calculated as the 10th lowest pixel value. This will not push any values
below zero (therefore, we aren't losing any information). It moves the
baseline up and looks prettier (improves signal to noise) without any
'ethical' concerns.

If images have already been quantified and you want to apply the concept
of this module without reprocessing your images, then multiply the
background threshold calculated by this module during the first image
cycle by the number of pixels in the image to get the number that should
be subtracted from the intensity measurements.

If you want to run this module only to calculate the proper threshold to
use, simply run the module as usual and use the button on the Status
window to stop processing after the first image cycle.

How it works:
Sort each image's pixel values and pick the 10th lowest pixel value as
the minimum. Typical images have a million pixels. The lowest pixel value
is chosen because it might be zero if it is a stuck pixel. It is quite
certain that there will not be 10 stuck pixels so this should be safe.
Then, take the minimum of these values from all the images. This scalar
value should be subtracted from every pixel in the image. CellProfiler is
not calculating a different value for each pixel position in the image
because in a small image set, that position may always be occupied by
real staining.

See also ApplyThreshold.
```

Module: Tile

```
Help for the Tile module:
Category: Image Processing

SHORT DESCRIPTION:
Creates one large, tiled image from all images of a certain type.
*************************************************************************

Allows many images to be viewed simultaneously, in a grid layout you
specify (e.g. in the actual layout in which the images were collected).

If you want to view a large number of images, you will generate an
extremely large file (roughly the size of all the images' sizes added
together) which, even if it could be created, could not be opened by any
image software anyway. There are several ways to allow a larger image to
be produced, given memory limitations: (1) Decrease the resolution of
each image tile by entering a fraction where requested. Then, in the
window which pops open after Tile finishes, you can use the 'Get high res
image' button to retrieve the original high resolution image. (Sorry,
this button is not yet functional). (2) Use the SpeedUpCellProfiler
module just before this module to clear out images that are stored in
memory. Place this module just prior to the Tile module (and maybe also
afterwards) and ask it to retain only those images which are needed for
downstream modules. (3) Rescale the images to 8 bit format by putting in
the RescaleIntensity module just prior to the Tile module. Normally
images are stored in memory as class "double" which takes about 10 times
the space of class "uint8" which is 8 bits. You will lose resolution in
terms of the number of different graylevels - this will be limited to 256
- but you will not lose spatial resolution.

The file name (automatic) and sample info (optional) can be displayed on
each image using buttons in the final figure window.

See also PlaceAdjacent.
```

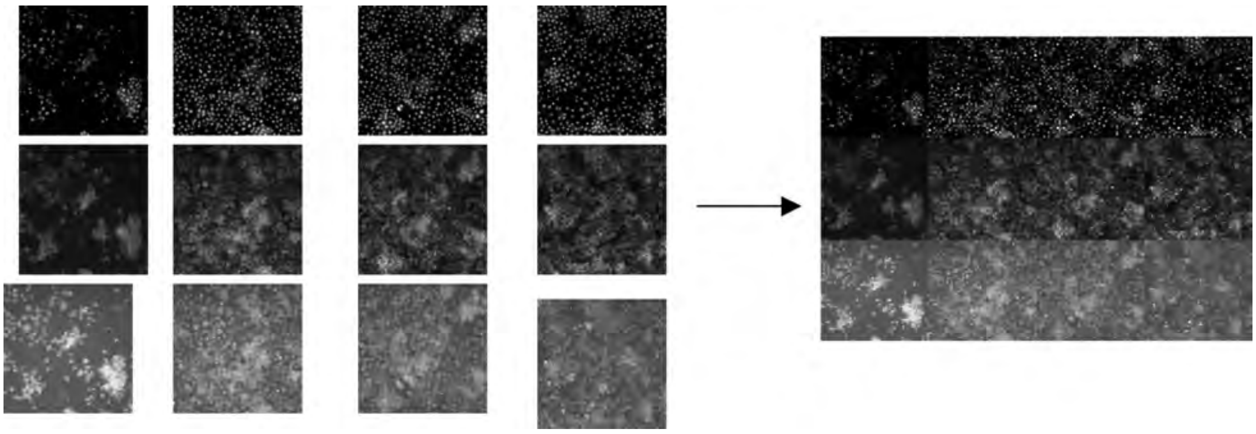

ImageTool: ImageToolWindow

Help for the Image Tool Window:
Category: Image Tools

SHORT DESCRIPTION:
The Image Tool Window opens when you click on any image and allows opening the image in a new window, displaying a pixel intensity histogram, measuring length in the image, changing the figure colormap, and saving the image.
****

The Image Tool Window contains these functions:

Open in new window - Opens the image in its own, fresh window.

Histogram - Shows a pixel intensity histogram for the image.

Measure Length - This tool creates a line in the image. By moving the ends of the line, you can measure distances in the image. Right-clicking the line reveals several options, including deleting the line. You can place multiple length-measuring lines on an image. Note that sometimes this line may interfere when saving the underlying image.

Change Colormap - Opens a window that allows you to change the colormap of the selected figure. You can select the default colormap (which you can set under File > Set Preferences) or any other predetermined colormap. Note that the colormap selected will apply to all non-RGB images in the entire figure, and not only to the image selected. The Apply To All button will change the colormap in all module display windows and any other windows that contain images. If you are running the developer's version of CellProfiler, you can also open a colormap editor, which enables you to create personalized colormaps. It will modify the colormap of the last active figure, so be careful if you open it, click another figure and go back to it, because you might be changing the colormap of a figure you did not intend to change. See also Help > General Help > Colormaps.

Save to Matlab workspace - If you are using Matlab Developer's version, this tool saves the image to the Matlab workspace with the variable name "Image". Be careful not to overwrite existing variables in your workspace using this tool.

Save to hard drive - Allows you to save the image to the hard drive. You can specify the file name, the directory where it will be saved, and a few other options. See the help for the Save Images module.

Technical details:
The CPimagetool function opens or updates the Image Tool window when the user clicks on an image produced by a module. The tool is embedded by the CPimagesc function which is used to display almost all images in CellProfiler.

ImageTool: InteractiveZoom

```
Help for the Interactive Zoom tool:
Category: Image Tools

SHORT DESCRIPTION:
Allows interactive zooming over the image.
*************************************************************************

This tool allows you zoom into an image by moving the cursor over it. The
image will automatically be zoomed to the location of the cursor. You can
control the zoom percentage with mouse clicks, which by default will be
set to 50% of the image in the axis. The tool will also open a black text
box showing the current position of the mouse pointer.

Mouse click commands:
* Left-clicking will zoom in further.
* Right-clicking will zoom out.
* Shift-clicking (or simultaneously clicking the left and right mouse
  buttons) at any point will display the original (un-zoomed) image, as
  will moving the cursor outside of the current axis. The zoom percentage
  is restored when the mouse is moved inside the axis.
* Double-clicking (either right or left button) zooms out to the original
  image, and resets the zoom percentage (i.e. it will not be restored as
  when shift-clicking).

The InteractiveZoom will work with all images in a figure, but only one
at a time. If you want to zoom into an image while currently zooming into
another, you will have to click on it to activate it, and you will lose
the zoom you had in the first image.

Note: there is a known bug that has no fix yet. When there are multiple
images in a figure, be careful not to double-click and move the pointer
fast enough such that one click lands on one image and the other lands in
another. This will create an error, although it will seldom happen.

To exit the InteractiveZoom, click the 'x' in the zooming pixel location
panel, or click on InteractiveZoom again in the menu (it will toggle
on/off each time you select it).
```

ImageTool: OpenNewImageFile

```
Help for the Open New Image File tool:
Category: Image Tools

SHORT DESCRIPTION:
Opens an image file in a new window.
*************************************************************************

Use this tool to open an image and display it. Images are loaded into
CellProfiler in the range of 0 to 1 so that modules behave consistently.
The display is contrast stretched so that the brightest pixel in the
image is white and the darkest is black for easier viewing.
```

ImageTool: ShowHelpForThisMenu

```
Help for the Show Toolbox Help function:
Category: Image Tools


SHORT DESCRIPTION:
Shows Help menu for various Image Toolboxes.
**********************************************************

Shows Help menu for various Image Toolboxes.
```

ImageTool: ShowOrHidePixelData

```
Help for the Show or Hide Pixel Data tool:
Category: Image Tools

SHORT DESCRIPTION:
Shows X,Y pixel location and intensity information in the figure window.
*********************************************************************

This tool shows the pixel intensity at each X,Y location as you hover
over points within an image. The pixels are displayed via a small box at
the lower left corner of the figure window. If the image is color (RGB),
three intensity values are shown: Red, Green, and Blue.

Currently, it can also measure lengths if you click the mouse at a
starting point and hold the button down while dragging, although this
could also be done with the Measure Length tool, accessible by clicking
on the image of interest and choosing Measure Length from the resulting
Image Tool window.

To exit the tool, click the 'x' in the pixel intensity information panel.
```

DataTool: AddData

Help for the Add Data tool:
Category: Data Tools

SHORT DESCRIPTION:
Allows adding information for each image cycle to an output file.
****
Note: this tool is beta-version and has not been thoroughly checked.

Use this tool if you would like to add text information about each image (e.g. Gene names or sample numbers) to the output file alongside the measurements that have been made. Then, the text information will be exported with the measurements when you use the ExportData data tool, helping you to keep track of your samples. You can also run the LoadText module in your pipeline so this step happens automatically during processing; its function is the same. Once the data is added to the output file, you can view the text file within the output file by using the ViewData data tool and selecting "Image". To delete the text file from the output file, use the ClearData data tool.

Note that the number of text entries that you load with this module must be identical to the number of cycles you are processing in order for exporting to work properly.

The information to be added must be in a separate text file with the following syntax:

DESCRIPTION <description>
<Text info for image cycle #1>
<Text info for image cycle #2>
<Text info for image cycle #3>
.
.

<description> is a description of the text information stored in the file. It can contain spaces or unusual characters.

For example:

DESCRIPTION Gene names
Gene X
Gene Y
Gene Z

While not thoroughly tested, most likely you can load numerical data too.

See also the LoadText module, ViewData and ClearData data tools.

DataTool: CalculateRatiosDataTool

Help for the Calculate Ratios data tool:
Category: Data Tools

SHORT DESCRIPTION:
Calculates the product, ratio, sum, or difference between any measurements already measured (e.g. Intensity of green staining in cytoplasm/Area of cells)
****

This data tool can take any measurements in a CellProfiler output file and multiply, divide, add, or subtract them. Resulting measurements can also be saved and used to calculate other measurements.

The data tool currently works on an object-by-object basis (it calculates the ratio for each object). If you need to calculate image-by-image ratios or ratios for object measurements by whole image measurements (to allow normalization), use the CalculateRatios module until this data tool is updated to handle such calculations. Be careful with your denominator data. Any 0's found in it may corrupt your output, especially when dividing measurements.

The new measurements will be stored under the first object's data, under the name Ratio.

See also CalculateRatios and all Measure modules.

DataTool: ClearData

Help for the Clear Data tool:
Category: Data Tools

SHORT DESCRIPTION:
Removes information/measurements from an output file.
****
Note: this tool is beta-version and has not been thoroughly checked.

This tool lets the user remove a measurement or data field from a CellProfiler output file. The same measurement can be removed from several files.

After executing this option, CelProfiler will ask the user to specify the output file(s) from which to remove data from. The user will then specify which data to clear. In most cases, the data to be cleared will be data providing information about an object.

DataTool: ConvertBatchFiles

```
Help for the Convert Batch Files tool:
Category: Data Tools

SHORT DESCRIPTION:
Converts output files produced by the Create Batch Files module into
typical CellProfiler output files.
*************************************************************************
Note: this tool is beta-version and has not been thoroughly checked.

CellProfiler data tools do not function on the batch output files created
by the Create Batch Files module because they are incomplete. They are
incomplete because each batch output file contains only the measurements
for one batch of images.

In order to access these measurements, they must be exported (using the
ExportDatabase data tool or ExportToDatabase module), or merged together
(using the MergeOutputFiles DataTool), or converted to regular
CellProfiler output files using this data tool. This data tool will save
new files with 'Converted' as a prefix in the filename.

Important: note that the image cycles will be renumbered, starting with
2. For example, your batch output file 'Batch_102_to_201_OUT.mat' will be
converted to 'ConvertedBatch_102_to_201_OUT.mat', but when you access the
data within (e.g. using ViewData), image cycle #102 will now be image
cycle #2. Image cycle #1 will be the original image cycle #1. Image cycle
#1 is present in all the batch files, and is removed so that the
converted batch file will contain only the remainder of the image cycles.

Technical details: this data tool removes empty entries in the
handles.Measurements structure of the output file(s) you specify.
```

DataTool: DataLayout

```
Help for the Data Layout tool:
Category: Data Tools

SHORT DESCRIPTION:
Shows mean measurements for each image in a specified spatial layout.
*************************************************************************
Note: this tool is beta-version and has not been thoroughly checked.

When images are collected in a particular spatial layout, it is
sometimes useful to view measurements collected from the images in the
same spatial layout to look for patterns (e.g. edge effects). The mean
measurement for each image is shown in the plot that is produced.
```

DataTool: ExportData

```
Help for the Export Data tool:
Category: Data Tools

SHORT DESCRIPTION:
Exports measurements into a tab-delimited text file which can be opened
in Excel or other spreadsheet programs.
*************************************************************************

Once image analysis is complete, use this data tool to select the output
file to extract the measurements and other information about the
analysis. The data will be converted to a tab-delimited text file which
can be read by Excel, another spreadsheet program, or a text editor. You
can add the ExportToExcel module to your pipeline if you want to
automatically export data.

See also ExportDatabase data tool, ExportToDatabase module, ExportToExcel
module.
```

DataTool: ExportDatabase

```
Help for the Export Database tool:
Category: Data Tools

SHORT DESCRIPTION:
Exports data in database readable format, including an importing file
with column names.
*************************************************************************

NOTE:
This tool is not functional right now - use the ExportToDatabase module
within your pipeline instead. Sorry for the inconvenience!!

This data tool exports measurements to a SQL compatible format. It creates
MySQL or Oracle scripts and associated data files which will create a
database and import the data into it. You can also run the ExportToDatabase
module in your pipeline so this step happens automatically during
processing; its function is the same.

See the help for the ExportToDatabase module for information on the
settings for this data tool and how to use it.

Current known limitations and things to consider:

- No check is performed that the selected files are compatible, i.e.
  were produced with the same pipeline of modules.

- The tool only works with standard CellProfiler output files, not
  batch output files. Use the ConvertBatchFiles data tool to convert if
  necessary.

- Image sets are numbered according to the order they are written by
  this tool. This numbering may not be consistent with the order they
  were processed, e.g. on the cluster. This can be fixed by adding an
  extra feature field in handles.Measurements.Image
```

DataTool: ExportLocations

```
Help for the Export Locations tool:
Category: Data Tools

SHORT DESCRIPTION:
Exports center locations of objects. Specialty function for creating a
locations list for microscopy image acquisition of gridded spots.
*************************************************************************

Useful for creating a locations list for microscope.
```

DataTool: GenerateHistogramMovie

```
Help for the Generate Histogram Movie tool:
Category: Data Tools

SHORT DESCRIPTION:
Creates a movie of the histogram of any measurement. This will be done
after specifying which output file the measurements exist in and where to
write the resulting .avi file.
*************************************************************************
Note: this tool is beta-version and has not been thoroughly checked.
```

DataTool: Histogram

```
Help for the Histogram tool:
Category: Data Tools

SHORT DESCRIPTION:
Displays a histogram of individual object measurements.
*************************************************************************
Note: this tool is beta-version and has not been thoroughly checked.

The object measurements can be displayed in histogram format using this
tool.  As prompted, select the output file containing the measurements,
then choose the measurement parameter to be displayed, and the sample
information label. It may take some time to then process the data.

SETTINGS:

* Which images' measurements to display or export - To display data from
only one image, enter that image's number as both the first and last
sample)

* The number of bins to be used

* Whether you want the histogram bins to contain the actual numbers of
objects in the bin or the percentage of objects in the bin

* How to determine the threshold values for the leftmost and rightmost
bins - on the Measurement axis (e.g. Area of Nuclei). For the leftmost
bin, any measurements less than the threshold will be combined in the
leftmost bin. For the rightmost bin, any measurements greater than or
equal to the thresholdd will be combined in the rightmost bin. Choosing
"Min/Max value found" will instruct CellProfiler to determine the
threshold values. Choosing "Other" will allow you to enter your custom
threshold values.

* Whether you want to calculate histogram data only for objects meeting a
threshold in a measurement - If you choose other than "None", you can
specify the type of threshold to use, and the threshold value.

* Whether you want to combine all the objects' data to be displayed in a
single (cumulative) histogram or in separate histograms

* Whether the X axis will be the "Measurements" axis (e.g. Area of
Nuclei) or the "Number of objects in bin" axis. The default for the X
axis is "Measurements". By choosing "Number of objects in bin", you are
essentially flipping the axes. Flipping is possible for both bar and
line graphs, but not area graphs because there is no function that will
work. If you attempt to flip an area graph, you will get a warning
message, and the display will be a normal unflipped area graph.

* For multiple histograms, whether you want the "Number of objects" axis
to be absolute (the same for all histograms) or relative (scaled to fit
the maximum value for that sample)
```

* Whether you want the axis to be log scale

* The style of the graph: bar, line, area, or heatmap

* The color that the inital plot should be

* Whether you want to display the histograms (Impractical when exporting large amounts of data).

* Whether you want to export the data - tab-delimited format, which can be opened in Excel. When entering the filename, use the extension ".xls" so it can be opened easily in Excel.

* Whether you want each row in the exported histogram or heatmap to contain an image or a bin

NOTES:

Measurement axis labels for histograms: Typically, the measurement axis labels will be too crowded. This default state is shown because you might want to know the exact values that were used for the histogram bins. The actual numbers can be viewed by clicking the 'This window' button under 'Change plots' and looking at the numbers listed under 'Labels'. To change the measurement axis labels, you can click 'Fewer' in the main histogram window, or you can click a button under 'Change plots' and either change the font size on the 'Style' tab, or check the boxes marked 'Auto' for 'Ticks' and 'Labels' on the 'X (or Y) axis' tab. Be sure to check both boxes, or the labels will not be accurate. To revert to the original labels, click 'Restore' in the main histogram window, but beware that this function does not work when more than one histogram window is open at once, because the most recently produced histogram's labels will be used for everything.

Change plots/change bars buttons: These buttons allow you to change properties of the plots or the bars within the plots for either every plot in the window ('This window'), the current plot only ('Current'), or every plot in every open window ('All windows'). This includes colors, axis limits and other properties.

Other notes about histograms: (1) Data outside the range you specified to calculate histogram bins are added together and displayed in the first and last bars of the histogram. (2) Only the display can be changed in this window, including axis limits. The histogram bins themselves cannot be changed here because the data must be recalculated. (3) If a change you make using the 'Change display' buttons does not seem to take effect in all of the desired windows, try pressing enter several times within that box, or look in the bottom of the Property Editor window that opens when you first press one of those buttons. There may be a message describing why. For example, you may need to deselect 'Auto' before changing the limits of the axes. (4) The labels for each bar specify the low bound for that bin. In other words, each bar includes data equal to

or greater than the label, but less than the label on the bar to its right.

See also PlotMeasurement data tool and DisplayHistogram and DisplayImageHistogram modules.

DataTool: MeasurementCalculator

```
Help for the Measurement Calculator tool:
Category: Data Tools

SHORT DESCRIPTION:
Multiplies or divides measurements in output files.
*************************************************************************
Note: this tool is beta-version and has not been thoroughly checked.

This tool allows you to multiply or divide data taken from CellProfiler
output files. You can choose the two measurements you wish to use, and
choose whether to multiply or divide them either objectwise, by the image
mean, or by the image median. You can give a name to your new measurement
and save it for later use.

See also CalculateRatiosDataTool, CalculateRatios.
```

DataTool: MergeOutputFiles

Help for the Merge Output Files data tool:
Category: Data Tools

SHORT DESCRIPTION:
Merges together output files produced by the Create Batch Files module into one regular CellProfiler output file.
****
Note: this module is beta-version and has not been thoroughly checked.

After a batch run has completed (using batch files created by the Create Batch Files module), the individual output files contain results from a subset of images and can be merged into a single output file. This module assumes anything matching the pattern of Prefix[0-9]*_to_[0-9]*.mat is a batch output file. The combined output is written to the output filename you specify. Once merged, this output file should be compatible with data tools.

Sometimes output files can be quite large, so before attempting merging, be sure that the total size of the merged output file is of a reasonable size to be opened on your computer (based on the amount of memory available on your computer). It may be preferable instead to import data from individual output files directly into a database - see the ExportDatabase data tool or the ExportToDatabase module.

Technical notes: The handles.Measurements field of the resulting output file will contain all of the merged measurement data, but handles.Pipeline is a snapshot of the pipeline after the first cycle completes.

See also: CreateBatchFiles, ExportDatabase data tool, ExportToDatabase module.

DataTool: PlotMeasurement

```
Help for the Plot Measurement tool:
Category: Data Tools

SHORT DESCRIPTION:
Plots measured data in bar charts, line charts, or scatterplots.
*************************************************************************
Note: this tool is beta-version and has not been thoroughly checked.

Bar charts, line charts and one dimensional scatter plots show the mean
and standard deviation of a measurement. Two dimensional scatter plots
allow plotting one measurement against another. As prompted, select a
CellProfiler output file containing the measurements, choose the
measurement parameter to be displayed, and choose the display type.

See also Histogram data tool.
```

DataTool: ShowDataOnImage

```
Help for the Show Data on Image tool:
Category: Data Tools

SHORT DESCRIPTION:
Produces an image with measured data on top of identified objects.
*************************************************************************
Note: this tool is beta-version and has not been thoroughly checked.

This tool allows you to extract measurements from an output file and
overlay any measurements that you have made on any image, very much like
the DisplayDataOnImage module. For example, you could look at the DNA
content (e.g. IntegratedIntensityOrigBlue) of each cell on an image of
nuclei. Or, you could look at cell area on an image of nuclei.

First, you are asked to select the measurement you want to be displayed
on the image. Next, if your output file has measurements from many
cycles, you are asked to select which sample/cycle number to view. Then,
you are asked to select an image to display the measurements over. You
can choose from among the list of images saved in the output file you
chose (which are generally the original images loaded by a LoadImages or
LoadSingleImage module), or you can browse for an image manually (e.g. a
cropped image that was created during the pipeline and saved on the disk
by a SaveImages module). You must try to select the image from which the
measurements were taken because the tool will try to display each
measurement over the corresponding object, so if the image is not the
right one, the data will make no sense. Once all these settings are
chosen, extraction ensues and eventually the image will be shown with the
measurements on top.

You can then use the InteractiveZoom under the CellProfiler Image Tools
menu to zoom in on this image. If the text is overlapping and not easily
visible, you can change the number of decimal places shown with the
'Significant digits' button, or you can change the font size with the
'Text Properties' button. You can also change the font style, color, and
other properties with this button. If you want to go back to the original
label settings, click the 'Restore labels' button. Alternatively, you can
hide and show the labels by clicking the 'Hide labels' and 'Show labels'
buttons, respectively.

The resulting figure can be saved in MATLAB format (.fig) or exported in
a traditional image file format.

See also DisplayDataOnImage.
```

DataTool: ViewData

```
Help for the View Data tool:
Category: Data Tools

SHORT DESCRIPTION:
Displays data or measurements from a CellProfiler output file. This is
displayed after the user specifies which output file and which
measurements to extract data from.
*************************************************************************
Note: this tool is beta-version and has not been thoroughly checked.

This tool views any text data or measurements that have been stored in a
CellProfiler output file. It can be useful to check that any text data
added with the AddData tool is associated with the correct image sets.
```
