# Supplementary material for: CellProfiler: image analysis software for identifying and quantifying cell phenotypes
Source: Genome Biol. 2006 Oct 31;7(10):R100. doi: 10.1186/gb-2006-7-10-r100 (PMC1794559; doi:10.1186/gb-2006-7-10-r100)
Supplement: Additional data file 2 — CellProfiler pipelines for experiments shown in this paper, listing the modules in the order used [file gb-2006-7-10-r100-S2.pdf]

A Drosophila full morphology analysis

LoadImages (to load each image)
LoadSingleImage (to load illum. functions)
CorrectIllumination_Apply (green channel)
CorrectIllumination_Apply (red channel)
CorrectIllumination_Apply (blue channel)
IdentifyPrimAutomatic (nuclei)
IdentifySecondary (cell edges)
IdentifyTertiarySubregion (cytoplasm)
MeasureCorrelation (blue, green, red in
nuclei, cells, cytoplasm, image)
MeasureObjectAreaShape (nuclei, cells)
MeasureObjectIntensity (blue in nuclei, cells, cytoplasm)
MeasureObjectIntensity (green in nuclei, cells, cytoplasm)
MeasureObjectIntensity (red in nuclei, cells, cytoplasm)
MeasureTexture (scale 1 texture - blue)
MeasureTexture (scale 1 texture - green)
MeasureTexture (scale 1 texture - red)
MeasureTexture (scale 3 texture - blue)
MeasureTexture (scale 3 texture - green)
MeasureTexture (scale 3 texture - red)
MeasureImageSaturationBlur (blue, green, red)
MeasureImageIntensity (blue)
MeasureImageIntensity (green)
MeasureImageIntensity (red)
ExportToDatabase
CreateBatchFiles

B Human full morphology analysis

Same as A, plus:
CalculateRatios
(Area nucleus/
Area cytoplasm)
MeasureObjectNeighbors
(nuclei)
MeasureObjectNeighbors
(cells)

C Human cytoplasm-nucleus translocation analysis

LoadImages (to load each image)
LoadSingleImage (to load illum. functions)
LoadText (to load dose info)
CorrectIllumination_Apply (green channel)
CorrectIllumination_Apply (blue channel)
IdentifyPrimAutomatic (nuclei)
IdentifyPrimAutomatic (cells, by simple thresholding)
IdentifySecondary (cell edges, propagate method)
IdentifySecondary (cell edges, distance method)
IdentifyTertiarySubregion (cytoplasm based on propagate cells)
IdentifyTertiarySubregion (cytoplasm based on distance cells)
MeasureCorrelation (blue, green in nuclei, cells, cytoplasm, image)
MeasureObjectIntensity (blue in nuclei, cells, cytoplasm)
MeasureObjectIntensity (green in nuclei, cells, cytoplasm)
MeasureObjectAreaShape (nuclei, cells, cytoplasm)
MeasureTexture (scale 1 texture - blue)
MeasureTexture (scale 1 texture - green)
CalculateRatios (Integrated green intensity in nuclei/cytoplasm
CalculateRatios (Mean intensity green in cytoplasm/nuclei
ClassifyObjects (Ratio 1 above 0.5)
ClassifyObjects (Extent of propagate-cytoplasm above 0.35)
ClassifyObjects (Ratio 2 above 0.5)
CalculateStatistics
SpeedUpCellProfiler

D Human speckle analysis

LoadImages (to load each image)
RescaleIntensity (blue channel)
RescaleIntensity (green channel)
RescaleIntensity (red channel)
IdentifyPrimAutomatic (nuclei)
IdentifyPrimAutomatic (speckles)
IdentifySecondary (cell edges)
IdentifyTertiarySubregion (cytoplasm)
Relate (speckles to nuclei)
MeasureObjectIntensity (red in nuclei,
cells, and cytoplasm)
MeasureObjectIntensity (blue in nuclei)
MeasureTexture (scale 3 texture - red)
OverlayOutlines
SaveImages (rescaled green)
SaveImages (outlined speckles)

E Illumination correction analysis

LoadImages (to load each image)
CorrectIllumination_Calculate (red channel)
SaveImages (red illumination function)
CorrectIllumination_Calculate (green channel)
SaveImages (green illumination function)
CorrectIllumination_Calculate (blue channel)
SaveImages (blue illumination function)
CreateBatchFiles

Additional Data File 2: CellProfiler pipelines, showing the modules in the order used, for experiments shown in this paper. The pipelines themselves, with their settings, are available at www.cellprofiler.org [31].
