# Supplementary material for: CellProfiler: image analysis software for identifying and quantifying cell phenotypes
Source: Genome Biol. 2006 Oct 31;7(10):R100. doi: 10.1186/gb-2006-7-10-r100 (PMC1794559; doi:10.1186/gb-2006-7-10-r100)
Supplement: Additional data file 3 — CellProfiler modules by category, with their descriptions [file gb-2006-7-10-r100-S3.pdf]

Additional Data File 3: CellProfiler modules by category, with their descriptions.

File Processing Modules	Description
CreateBatchFiles	Produces text files which allow individual batches of images to be processed separately on a cluster of computers.
ExportToExcel	Exports measurements into a tab-delimited text file which can be opened in Excel or other spreadsheet programs.
ExportToDatabase	Exports data in database readable format, including an importing file with column names.
LoadImages	Allows you to specify which images or movies are to be loaded and in which order. Groups of images will be loaded per cycle of CellProfiler processing.
LoadSingleImage	Loads a single image, which will be used for all image cycles.
LoadText	Loads text information corresponding to images. This data (e.g. gene names or sample numbers) can be displayed on a grid or exported with the measurements to help track samples.
RenameOrRenumberFiles	Renames or renumbers files on the hard drive.
Restart	Restarts image analysis which had failed or was canceled, using the partially completed output file.
SaveImages	Saves any image produced during the image analysis, in any image format.
SplitOrSpliceMovie	Creates one large movie from several small movies, or creates several small movies from one large movie.
**Image Processing Modules**	**Description**
Align	Aligns two or three images relative to each other. Particularly useful to align microscopy images acquired from different color channels.
ApplyThreshold	Pixels below (or above) a certain threshold are set to zero. The remaining pixels retain their original value or (optional) are shifted to match the threshold used.
Average	Averages images together (makes a projection).
ColorToGray	Converts RGB (Red, Green, Blue) color images to grayscale. All channels can be merged into one grayscale image or each channel can extracted into a separate grayscale image.
CorrectIllumination_Apply	Applies an illumination function, created by CorrectIllumination_Calculate, to an image in order to correct for uneven illumination (uneven shading).
CorrectIllumination_Calculat	Calculates an illumination function, used to correct uneven illumination/lighting/shading or to reduce uneven background in images.
Crop	Crops images into a rectangle, ellipse, an arbitrary shape provided by the user, a shape identified by an identify module, or a shape used at a previous step in the pipeline on another image.
Flip	Flips an image from top to bottom, left to right, or both.
GrayToColor	Takes 1 to 3 images and assigns them to colors in a final red, green, blue (RGB) image. Each color's brightness can be adjusted independently.

InvertIntensity	Converts the intensities of a grayscale image so that black becomes white.
OverlayOutlines	Places outlines produced by an identify module over a desired image.
PlaceAdjacent	Places up to six images next to each other, either horizontally or vertically, to produce a single image.
RescaleIntensity	Changes intensity range of an image to desired specifications.
Resize	Resizes images.
Rotate	Rotates images either automatically or based on the user clicking.
Smooth	Smooths (blurs) images.
Subtract	Subtracts the intensities of one image from another.
SubtractBackground	Calculates the minimum pixel intensity value for the entire set of images and subtracts this value from every pixel in every image.
Tile	Creates one large, tiled image from all images of a certain type.
**Object Processing Modules**	**Description**
ClassifyObjects	Classifies objects into different classes according to the value of a measurement you choose.
ConvertToImage	Converts objects you have identified into an image so that it can be saved with the Save Images module.
Exclude	Removes objects outside of specified region.
ExpandOrShrink	Expands or shrinks identified objects by a defined distance.
FilterByObject Measurement	Eliminates objects based on their measurements (e.g. area, shape, texture, intensity).
IdentifyObjectsInGrid	Identifies objects within each section of a grid that has been defined by the Define Grid module.
IdentifyPrim Automatic	Identifies objects given only an image as input.
IdentifyPrim Manual	Identifies an object based on manual intervention (clicking) by the user.
IdentifySecondary	Identifies objects (e.g. cell edges) using "seed" objects identified by an Identify Primary module (e.g. nuclei).
IdentifyTertiary Subregion	Identifies tertiary obects (e.g. cytoplasm) by removing the primary objects (e.g. nuclei) from secondary objects (e.g. cells) leaving a ring shape.
Relate	Assigns relationships: All objects (e.g. speckles) within a parent object (e.g. nucleus) become its children.
**Measurement Modules**	**Description**
CalculateRatios	Calculates the ratio between any measurements already measured (e.g. Intensity of green staining in cytoplasm/Area of cells)
CalculateStatistics	Calculates the V and Z' factors for measurements made from images.
MeasureCorrelation	Measures the correlation between intensities in different images (e.g. different color channels) on a pixel by pixel basis, within identified objects or across an entire image.
MeasureImageArea Occupied	Measures total area covered by stain in an image.
MeasureImage Intensity	The user can choose to ignore pixels below or above a particular intensity level.

MeasureImage SaturationBlur	Measures the percentage of pixels in the image that are saturated and measures blur (poor focus).
MeasureObject AreaShape	Measures several area and shape features of identified objects.
MeasureObject Intensity	Measures several intensity features for identified objects.
MeasureObject Neighbors	Calculates how many neighbors each object has.
MeasureTexture	Measures several texture features for identified objects or for entire images.
**Other Modules**	**Description**
CreateWebPage	Creates the html for a webpage to display images (or their thumbnails, if desired), including a link to a zipped file with all of the included images.
DefineGrid	Produces a grid of desired specifications either manually or automatically, based on previously identified objects. The grid can then be used to make measurements (using Identify Objects in Grid) or to display text information (using Display Grid Info) within each compartment of the grid.
DisplayDataOnImage	Produces an image with measured data on top of identified objects.
DisplayGridInfo	Displays text info on grid (i.e. gene names).
DisplayHistogram	Produces a histogram of measurements.
DisplayImage Histogram	Produces a histogram of the intensity of pixels within an image.
DisplayMeasurement	Plots measured data in bar charts, line charts, or scatter plots.
SendEmail	Sends emails to a specified address at desired stages of the processing.
SpeedUpCellProfiler	Prevents saving partial output files after every image cycle and/or clears the memory.
