# Supplementary material for: CellProfiler: image analysis software for identifying and quantifying cell phenotypes
Source: Genome Biol. 2006 Oct 31;7(10):R100. doi: 10.1186/gb-2006-7-10-r100 (PMC1794559; doi:10.1186/gb-2006-7-10-r100)
Supplement: Additional data file 4 — Measurements made by CellProfiler modules [file gb-2006-7-10-r100-S4.pdf]

Additional Data File 4: Measurements made by CellProfiler modules

Feature name	Feature description
**Location features in the Identify modules**	
CenterX	The X location of the object
CenterY	The Y location of the object
Threshold	The threshold used in processing the image, if applicable.
Object Count	Number of objects found in the image.
**Basic area and shape features in the MeasureObjectAreaShape module**	
Area	Computed from the actual number of pixels in the region.
Eccentricity	Also known as elongation or elongatedness. For an ellipse that has the same second-moments as the object, the eccentricity is the ratio of the between-foci distance and the major axis length. The value is between 0 (a circle) and 1 (a line segment).
Solidity	Also known as convexity. The proportion of the pixels in the convex hull that are also in the object. Computed as Area/ConvexArea.
Extent	The proportion of the pixels in the bounding box that are also in the region. Computed as the Area divided by the area of the non-rotated bounding box.
Euler number	Equal to the number of 'objects' in the image minus the number of holes in those objects. For modules built to date, the number of 'objects' in the image is always 1.
Perimeter	The number of pixels around the boundary of the image.
Form factor	= 4*pi*Area/(Perimeter^2)<br>= 1 for a perfectly circular object
Major Axis Length	The length (in pixels) of the major axis of the ellipse that has the same normalized second central moments as the region.
Minor Axis Length	The length (in pixels) of the minor axis of the ellipse that has the same normalized second central moments as the region.
**Zernike shape features in the MeasureObjectAreaShape module**	
30 Zernike polynomials, from order 0 to order 9	See manual/CellProfiler help for description
**Features in the MeasureObjectIntensity module**	
Integrated Intensity	The sum of the pixel intensities within an object
Mean Intensity	The average pixel intensity within an object
Std Intensity	The standard deviation of the pixel intensities within an object
Max Intensity	The maximal pixel intensity within an object
Min Intensity	The minimal pixel intensity within an object
Integrated Intensity Edge	The sum of the edge pixel intensities of an object
Mean Intensity Edge	The average edge pixel intensity of an object
Std Intensity Edge	The standard deviation of the edge pixel intensities of an object
Max Intensity Edge	The maximal edge pixel intensity of an object
Min Intensity Edge	The minimal edge pixel intensity of an object

Mass Displacement	The distance between the centers of gravity in the gray-level representation of the object and the binary representation of the object
**Features in the MeasureTexture module (can also measure the entire image)**	
Angular Second Moment	Haralick's measure H1
Contrast	Haralick's measure H2
Correlation	Haralick's measure H3
Sum of Squares: Variation	Haralick's measure H4
Inverse Difference Moment	Haralick's measure H5
Sum Average	Haralick's measure H6
Sum Variance	Haralick's measure H7
Sum Entropy	Haralick's measure H8
Entropy	Haralick's measure H9
Difference Variance	Haralick's measure H10
Difference Entropy	Haralick's measure H11
Information Measure of Correlation 1	Haralick's measure H12
Information Measure of Correlation 2	Haralick's measure H13
H14. Max correlation coefficient	Haralick's measure H14 Note: disabled by default because it is computationally demanding.
Gabor X	Gabor "wavelet" feature - See manual/CellProfiler help for description
Gabor Y	Gabor "wavelet" feature - See manual/CellProfiler help for description
**Correlation features in the MeasureCorrelation module**	
Correlation	Correlation coefficients for the pixel intensities are calculated for each pair of images specified, and can be calculated for the entire image overall, or within each individual object.
**Features in the MeasureNeighbors module**	
Number of Neighbors	Number of touching neighbors for each object
Identity of Neighbors	Lists the label (identification number) of the neighbors that each object has.
**Features in the MeasureImageIntensity module**	
Total Intensity	Adds up the pixel intensities across the entire image, subject to some user specifications
Mean intensity	Averages the pixel intensities across the entire image, subject to some user specifications
Total area	Records how much area of the image was actually used for the above calculations (after certain regions were discarded according to the user's specifications)
**Features in the MeasureImageAreaOccupied module**	
Image Area Occupied	Measures how much area is occupied by staining in an image, after applying a threshold

Image Area Occupied Threshold	The threshold used in processing the image
**Features in the MeasureImageSaturationBlur module**	
Blur Score	Normalized variance, the best measure of blur (poor focus) reported in (Sun et al., 2004)
Percent Saturated	The percentage of pixels in the image that are saturated (at the maximum possible intensity value for the image). Useful for quality control.
**Features in other modules**	
Filenames and Pathnames	LoadImages and LoadSingleImage modules
Image X Align and Image Y Align	Align module: the number of pixels the images were shifted in each direction in order to align them.
User-defined ratios	CalculateRatios module
V factor and Z' factor	CalculateStatistics module: statistical measures of assay quality including V factor (Ravkin, 2004) and Z' factor (Zhang et al., 1999).
User-defined classifications	ClassifyObjects module: the fraction or absolute number of objects in each classification 'bin'
Grid-related data	DefineGrid module
User-loaded data	LoadText module
Rotation	Rotate module: the angle of rotation of the image
IntensityToShift	SubtractBackground module: the background pixel intensity value to subtract from all images.
