# Supplementary material for: CellProfiler: image analysis software for identifying and quantifying cell phenotypes
Source: Genome Biol. 2006 Oct 31;7(10):R100. doi: 10.1186/gb-2006-7-10-r100 (PMC1794559; doi:10.1186/gb-2006-7-10-r100)
Supplement: Additional data file 5 — Data and image tools in CellProfiler, with their descriptions [file gb-2006-7-10-r100-S5.pdf]

Additional Data File 5: Data and image tools in CellProfiler, with their descriptions.

Data Tools	Description
AddData	Adds text information for each image to an output file (e.g. gene name, sample information).
CalculateRatiosDataTool	Calculates the ratio between any measurements already measured (e.g. Intensity of green staining in cytoplasm/Area of cells)
ClearData	Removes information/measurements from an output file.
ConvertBatchFiles	Converts output files produced by the Create Batch Files module into regular CellProfiler output files.
DataLayout	Shows mean measurements for each image in a specified spatial layout.
ExportData	Exports measurements into a tab-delimited text file which can be opened in Excel or other spreadsheet programs.
ExportDatabase	Exports measurements in database format.
ExportLocations	Exports center locations of objects. Specialty function for creating a locations list for microscopy image acquisition of gridded spots.
GenerateHistogramMovie	Creates a movie of the histogram of any measurement.
Histogram	Displays a histogram of individual object measurements.
MeasurementCalculator	Multiplies or divides measurements in output files.
MergeOutputFiles	Merges together output files produced by the Create Batch Files module into one regular CellProfiler output file.
PlotMeasurement	Plots measured data in bar charts, line charts, or scatterplots.
ShowDataOnImage	Produces an image with measured data on top of identified objects.
ViewData	Displays data or measurements from a CellProfiler output file.
**Image tools**	**Description**
ImageToolWindow	The Image Tool Window opens when you click on any image and allows opening the image in a new window, displaying a pixel intensity histogram, measuring length in the image, and saving the image.
InteractiveZoom	Allows interactive zooming over the image.
OpenNewImageFile	Opens an image file in a new window.
ShowOrHidePixelData	Shows X,Y pixel location and intensity information in the Fig window.
