# Supplementary material for: CellProfiler: image analysis software for identifying and quantifying cell phenotypes
Source: Genome Biol. 2006 Oct 31;7(10):R100. doi: 10.1186/gb-2006-7-10-r100 (PMC1794559; doi:10.1186/gb-2006-7-10-r100)
Supplement: Additional data file 6 — Example from CellProfiler analysis of DNA content (cell cycle) in Drosophila Kc167 cells [file gb-2006-7-10-r100-S6.pdf]

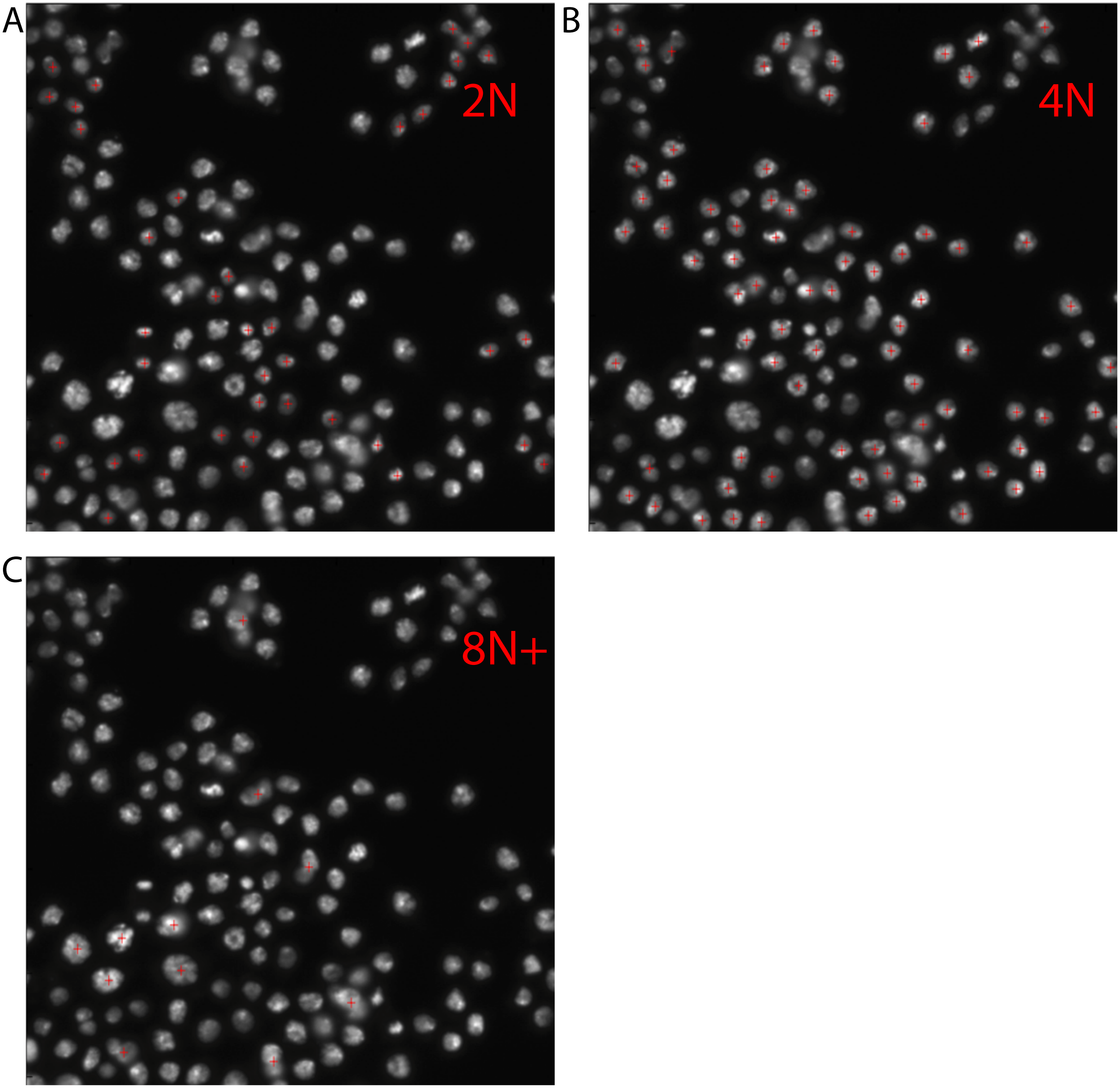

Additional Data File 6: Example from CellProfiler analysis of DNA content (cell cycle) in Drosophila Kc167 cells. The image shown is the top left quadrant of the first of a random image from a large image set. Based on DNA content (integrated intensity of DNA staining within the nucleus), cells within each of the following categories are marked with red plus signs: (A) 2N DNA content, (B) 4N DNA content, (C) 8N+ DNA content. Scale bar = 10 um.
