# Supplementary material for: CellProfiler: image analysis software for identifying and quantifying cell phenotypes
Source: Genome Biol. 2006 Oct 31;7(10):R100. doi: 10.1186/gb-2006-7-10-r100 (PMC1794559; doi:10.1186/gb-2006-7-10-r100)
Supplement: Additional data file 7 — Histograms of shape and texture features for wild-type cells [file gb-2006-7-10-r100-S7.pdf]

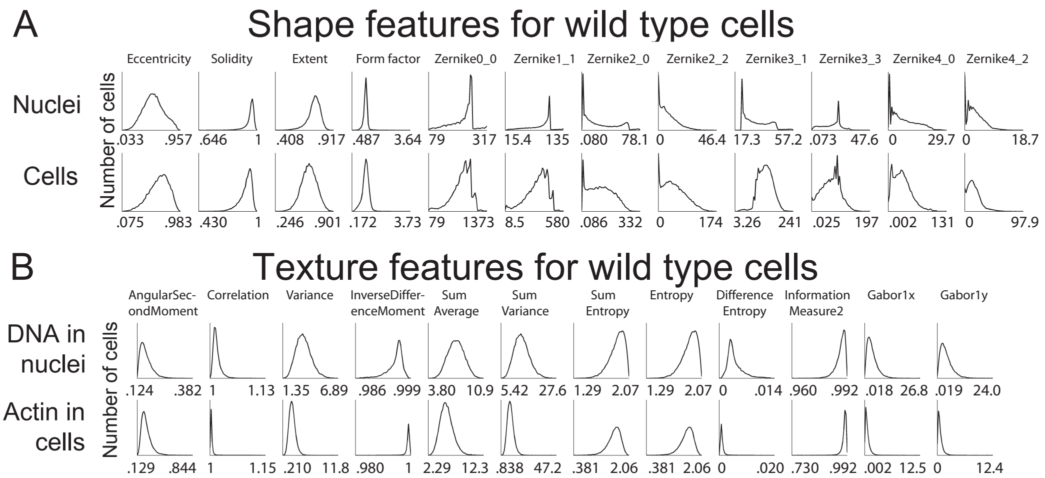

Additional Data File 7: Histograms of shape and texture features for wild type cells. A. Some shape features are shown as histograms for wild type Drosophila Kc167 nuclei and cells, from 1750 images. B. Some actin texture and DNA texture features are shown as histograms for wild type Drosophila Kc167 cells, from 1750 images (stained with Hoechst and phalloidin).
