# Supplementary material for: CellProfiler: image analysis software for identifying and quantifying cell phenotypes
Source: Genome Biol. 2006 Oct 31;7(10):R100. doi: 10.1186/gb-2006-7-10-r100 (PMC1794559; doi:10.1186/gb-2006-7-10-r100)
Supplement: Additional data file 8 — Measures for the cytoplasm-nucleus translocation assay (Figure 4) for which the Z' factor is above 0.5 [file gb-2006-7-10-r100-S8.pdf]

Additional Data File 8: Measures for the cytoplasm-nucleus translocation assay (Fig 4) for which the Z' factor is above 0.5.

		Wortmannin	LY294002
**Measure**	Z'-factor	V-factor	V-factor
Thresholded DistCytoplasm/Nuclei Intensity	0.91	0.86	0.84
Correlation Green and Blue in DistanceCells	0.8	0.84	0.8
Correlation Green and Blue in Nuclei	0.76	0.84	0.8
Correlation Green and Blue in ThresholdedCells	0.73	0.74	0.69
Correlation Green and Blue in PropCells	0.71	0.8	0.76
Mean Intensity Green in DistCytoplasm/Nuclei	0.7	0.78	0.72
Integrated Intensity Green in Nuclei/DistCytoplasm	0.7	0.7	0.61
